# Supplementary material for: Phase 1b trial of anti-EGFR antibody JMT101 and Osimertinib in EGFR exon 20 insertion-positive non-small-cell lung cancer
Source: Nat Commun. 2023 Jun 12;14:3468. doi: 10.1038/s41467-023-39139-4 (PMC10261012; doi:10.1038/s41467-023-39139-4)
Supplement: Supplementary file 1 — Supplementary Information [file 41467_2023_39139_MOESM1_ESM.pdf]

Supplementary Information

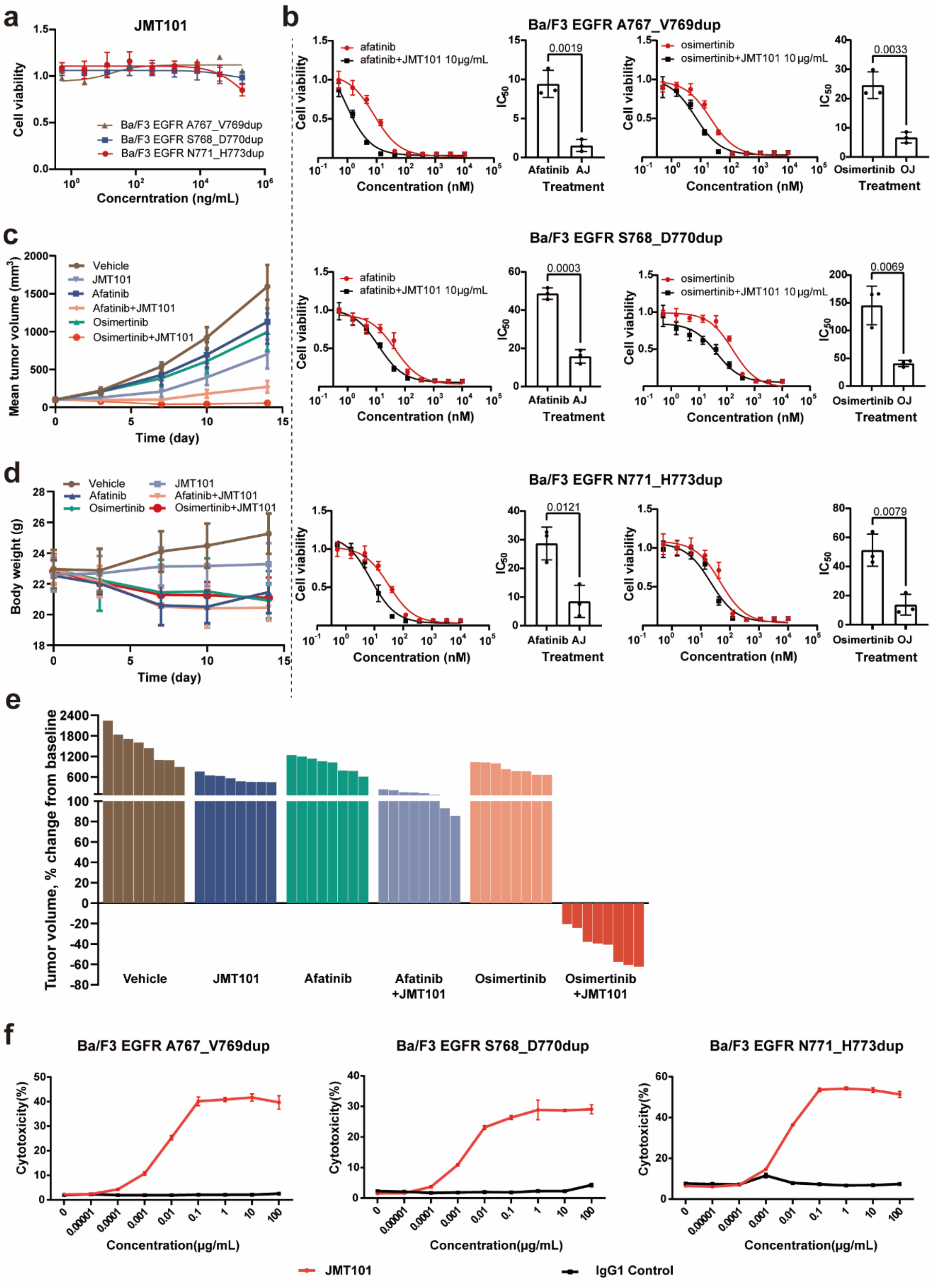

Supplementary Figure 1. Antitumor activity of JMT101 plus afatinib or osimertinib in Ba/F3 cells expressing EGFR 20ins and in xenograft models.

a. Dose-response curves of JMT101 monotherapy in Ba/F3 cells expressing EGFR insASV, insSVD and insNPH; **b.** Dose-response curves of afatinib versus JMT101 + afatinib, osimertinib versus JMT101 + osimertinib in Ba/F3 cells expressing EGFR insASV, insSVD and insNPH; Data are analyzed by two-sided Student's t test; **c.** Mean changes in tumor volume from baseline in BALB/c nude mice subcutaneously injected with Ba/F3 cells expressing EGFR insASV receiving vehicle po. qd; JMT101 50 mg/kg iv. biw; Afatinib 15 mg/kg po. qd; Afatinib 15 mg/kg po. qd + JMT101 50 mg/kg iv. biw; Osimertinib 25 mg/kg po. qd; Osimertinib 25 mg/kg po. qd + JMT101 50 mg/kg iv. biw; **d.** Weight changes from baseline in BALB/c nude mice models in the above treatment groups; **e.** Percentage changes in tumor volume in BALB/c nude mice models in the above treatment groups; **f.** JMT101-mediated ADCC activity in Ba/F3 cells expressing EGFR insASV, insSVD and insNPH using NK cells as effector cells (E:T ratio = 4: 1). Statistical analyses were performed between JMT101 and IgG1 control of the same concentration with a one-sided Student's t test (results are shown in the source data). Data are presented as averages ± standard deviations of three independent experiments. Source data are provided as a source data file.

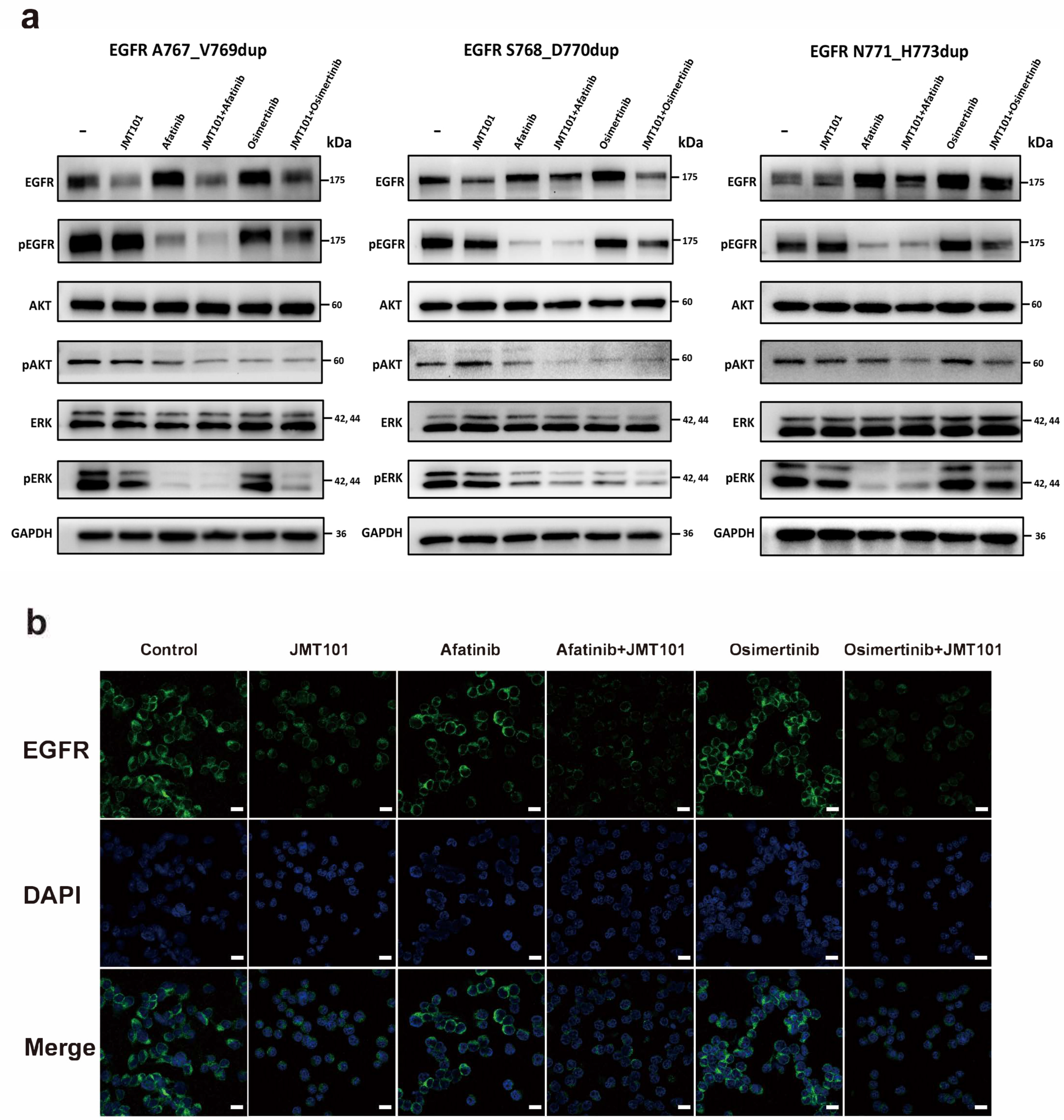

Supplementary Figure 2. JMT101 plus afatinib or osimertinib lead to a more thorough EGFR blockade in Ba/F3 cells expressing EGFR 20ins.

a. Ba/F3 cells expressing the indicated EGFR 20ins were treated with 10ug/mL JMT101 or 100nmol/L EGFR TKI or 100nmol/L EGFR TKI plus 10ug/mL JMT101 for 6 hours; Immunoblot analysis was performed after indicated treatment; Each experiment were repeated independently three times; b. IF staining for EGFR (green) in a panel of Ba/F3 cells expressing EGFR insASV treated with 10ug/mL JMT101 or 100nmol/L EGFR TKIs or 100nmol/L EGFR TKIs plus 10ug/mL JMT101 for 24 hours. Scale bar = 10um. Source data are provided as a source data file.

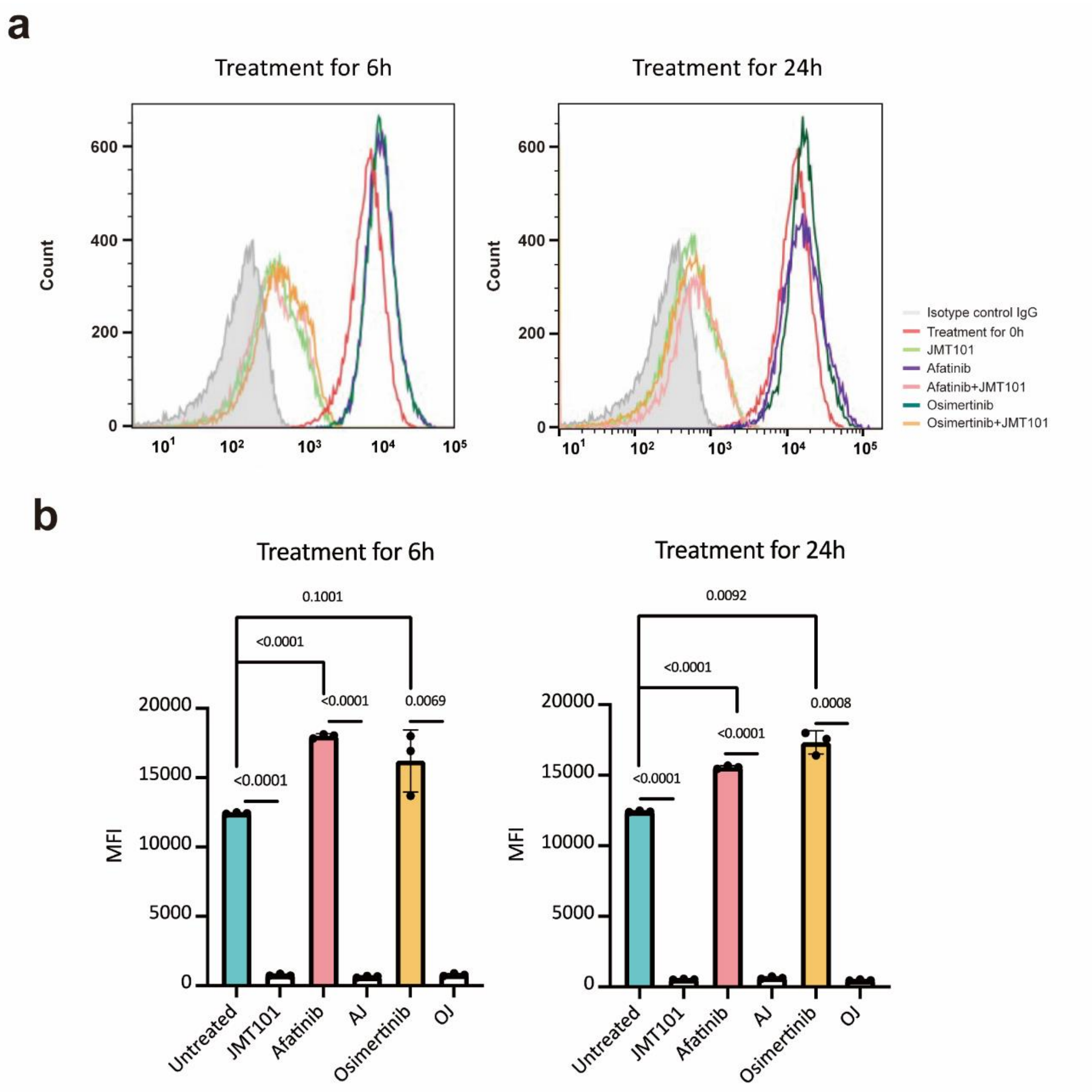

Ba/F3 EGFR A767_V769dup

group	6h MFI	24h MFI
Treatment for 0h	12434	12434
JMT101	771.7	541
Afatinib	17997	15569
Afatinib+JMT101	649	649.7
Osimertinib	16203	17325
Osimertinib+JMT101	789.7	485

Supplementary Figure 3. JMT101 plus afatinib or osimertinib induced EGFR internalization and downregulation in Ba/F3 cells expressing EGFR A767_V769dup.

a. EGFR expression on the plasma membrane was detected in Ba/F3 cells expressing EGFR insASV through flow cytometry. After treatment with 10ug/mL JMT101 or 100nmol/L EGFR TKIs or 100nmol/L EGFR TKIs plus 10ug/mL JMT101 for 6 hours or 24 hours, FITC-EGFR expression on the plasma membrane was detected. **b.** Summary of the mean MFI (median fluorescence intensity) of each group. Data in 3b were presented as mean ± S.D., n = 3 biologically independent samples. Error bars reflect mean ± standard deviation. Statistical analysis was performed using the two-sided Student's t test. Source data are provided as a source data file.

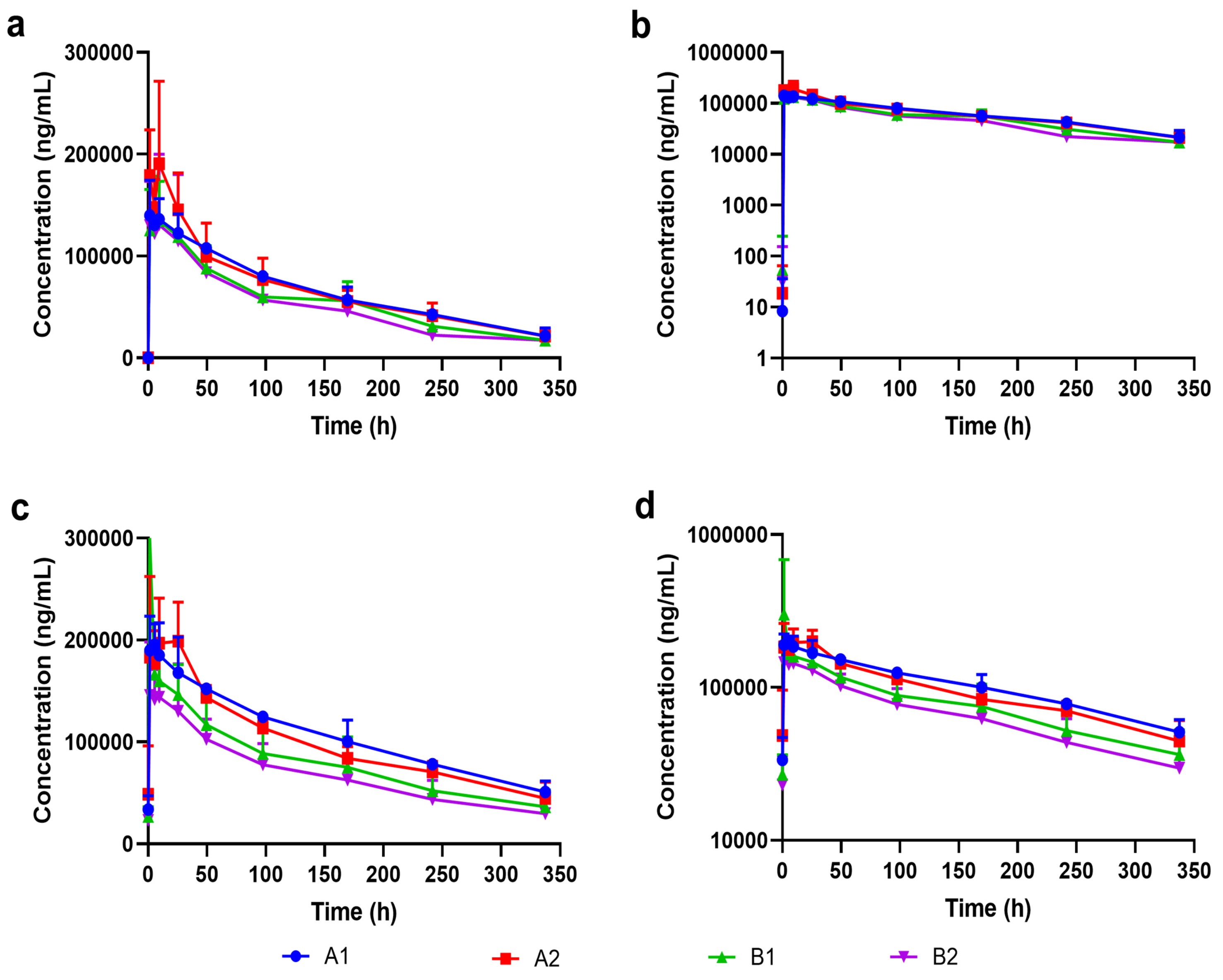

Supplementary Figure 4. Plasma concentration-time curves of JMT101 at the dose of 6 mg/kg.

a. Linear plots after single dose; b. Semi-logarithmic plots after single dose; For a and b, A1 cohort: n=11; A2 cohort: n=6; B1 cohort: n=12; B2 cohort: n=109, n refers to the number of plasma samples; c. Linear plots after the third dose; d. Semi-logarithmic plots after the third dose. For c and d, A1 cohort: n=11; A2 cohort: n=6; B1 cohort: n=11; B2 cohort: n=100, n refers to the number of plasma samples. Error bars represent standard deviation.

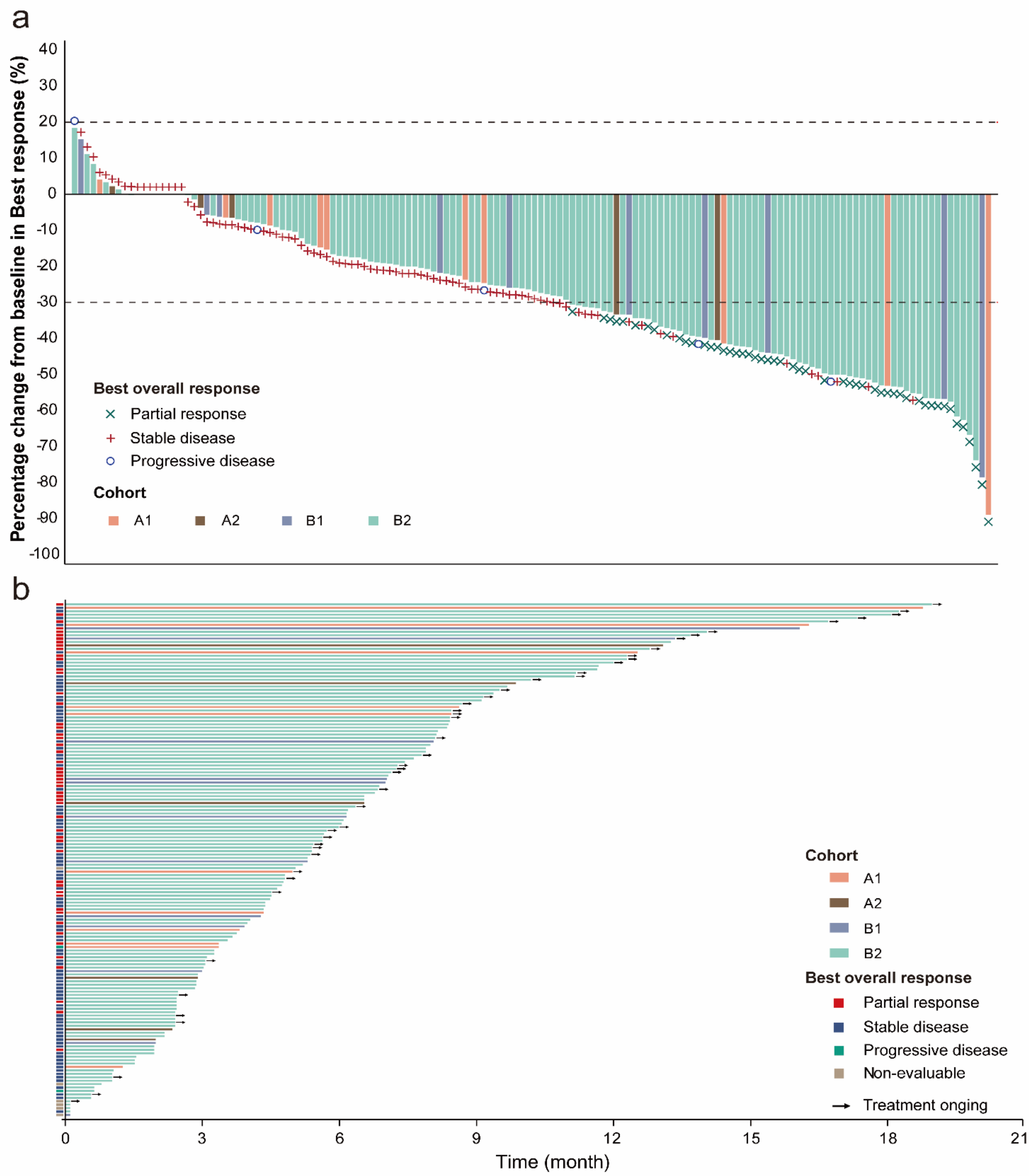

Supplementary Figure 5. Activity of JMT101 plus afatinib or osimertinib in the overall population.

a. Maximal percentage change in the sum of target lesions from baseline based on IRC assessment; **b.** Duration of treatment and best of response in patients per IRC assessment.

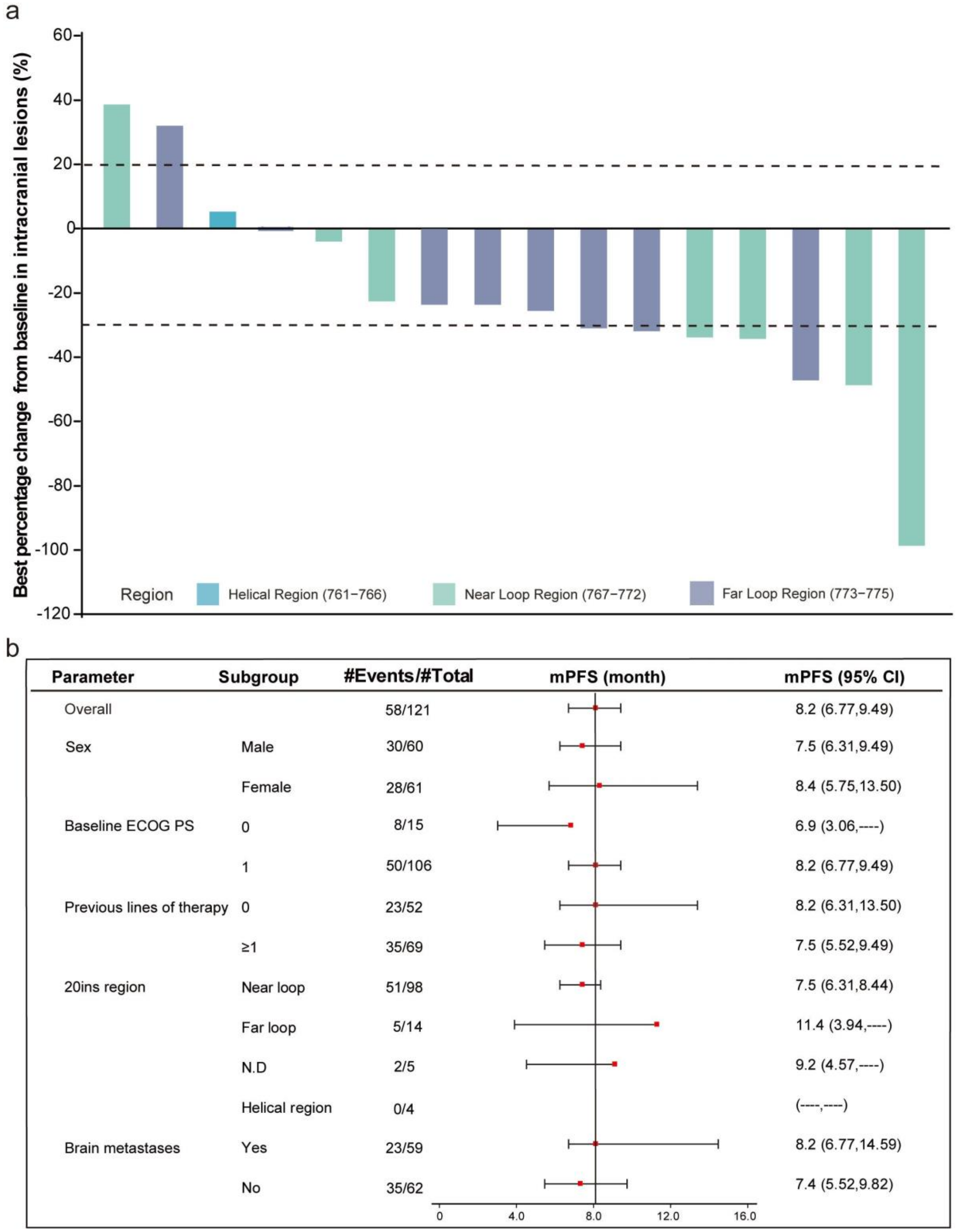

Supplementary Figure 6. Intracranial response and subgroup analysis of PFS in the efficacy population

a. Intracranial response in patients with brain metastases as target lesions (n=16). Maximal percentage change in the sum of target lesions from baseline per investigator-assessment. Dotted lines at 20% and -30% indicate cutoffs for progressive disease and partial response per RECIST v1.1, respectively. **b.** Post hoc subgroup analysis of PFS in the efficacy population. PFS are estimated using the Kaplan-Meier method and are compared using the non-parametric log-rank test. PFS are presented as median (center) with 95% confidence interval (error bar). #Events stands for the number of patients that have reported the event of PFS. #Total stands for the number of the patient in the subgroup.

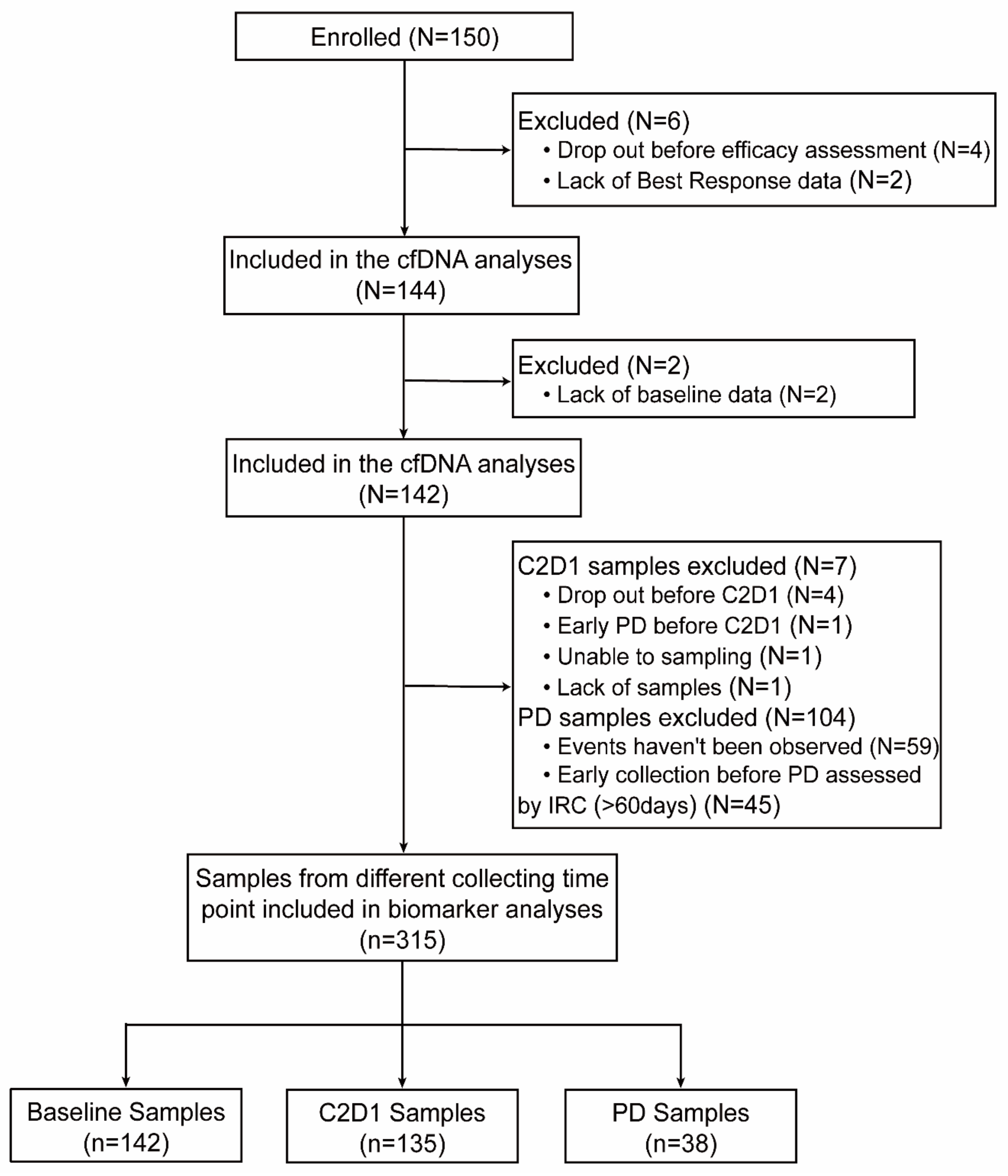

Supplementary Figure 7. Sample distribution for biomarker analysis.

cfDNA, circulating free DNA; C2D1, cycle 2 day 1; PD, disease progression.

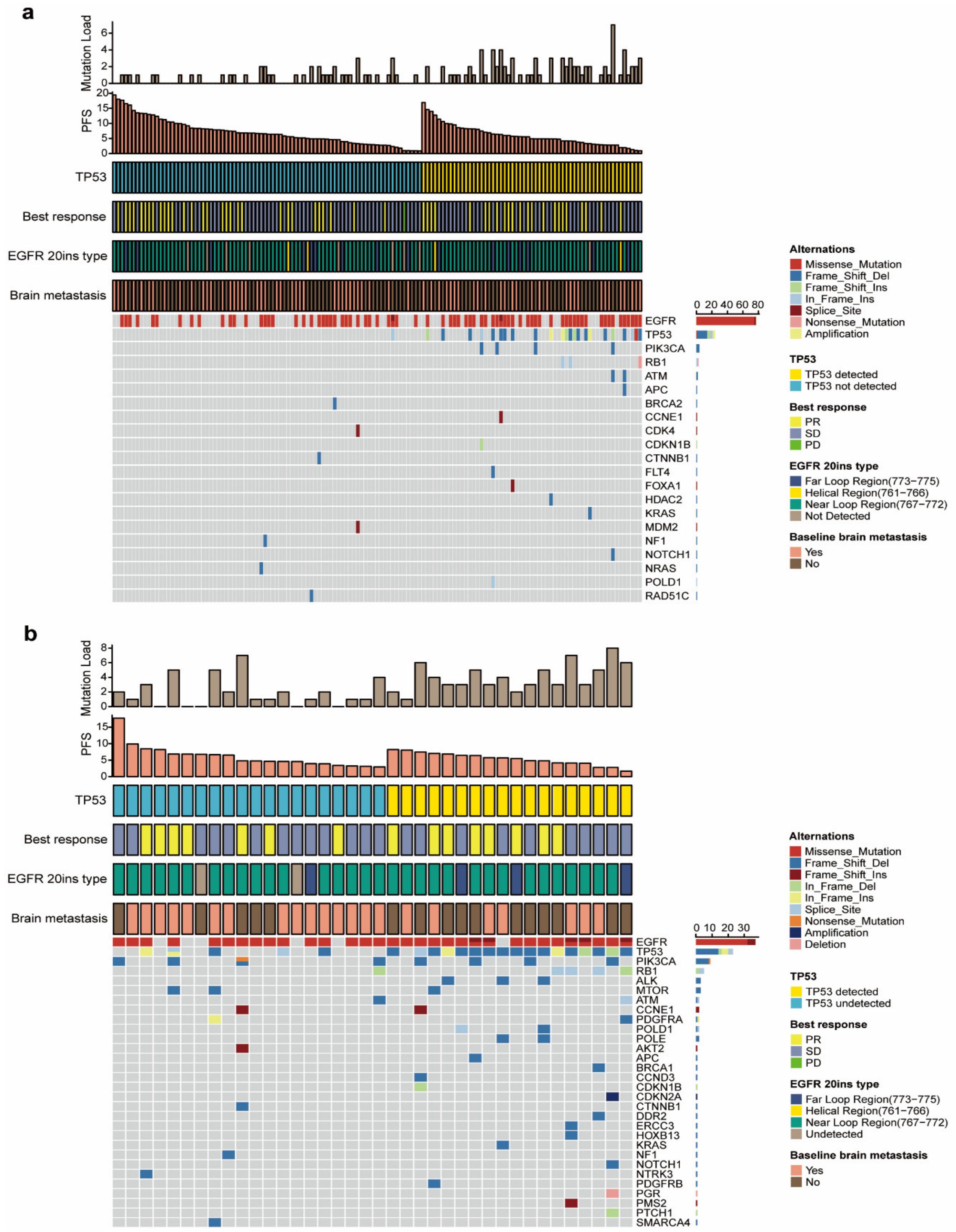

Supplementary Figure 8. Tumor mutation landscapes in patients at C2D1 (n=135, a) and at disease progression (n=38, b).

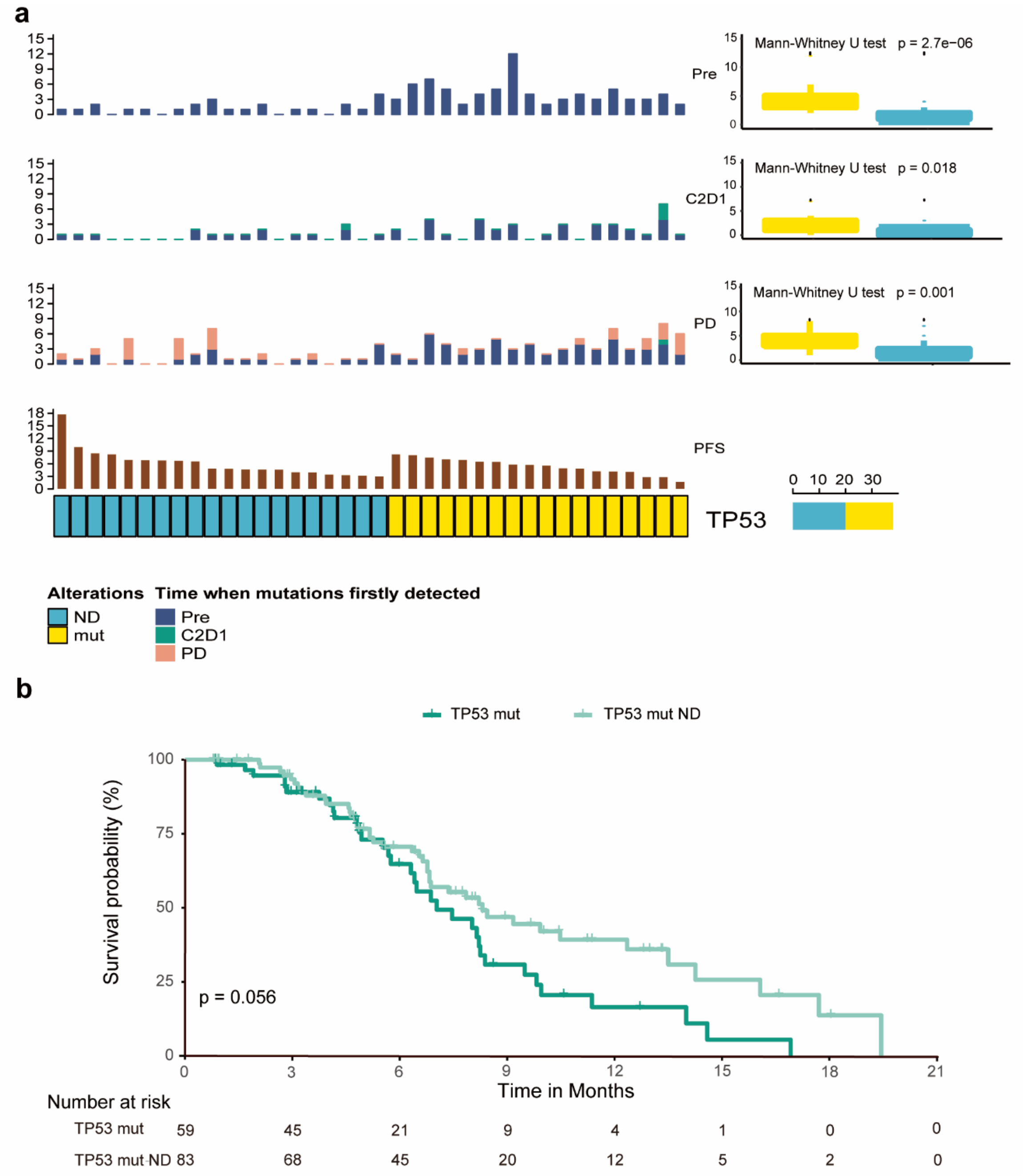

Supplementary Figure 9. Clinical impacts of concurrent TP53 alterations detected at baseline.

a. Changes in tumor mutation load during the course of treatment in patients with TP53-altered tumors versus those with TP53-wildtype at baseline, C2D1 and PD. Data are derived from 38 biologically independent samples (N=38), of which $N_{TP53\ ND}$=20, $N_{TP53\ mut}$=18. Boxplots display box limits, whiskers and outliers, which can be calculated by IQR

(Inter Quartile Range, Q3-Q1). The center line in the box plots represents the median; the upper limit of the box plots represents the 75th percentile (Q3); the lower limit of the box plots represents the 25th percentile (Q1); the upper whisker is the maximum value of Q3+1.5IQR; and the lower whisker is the minimum value of Q1-1.5IQR. The outlier is defined as a value less than Q1-1.5IQR or greater than Q3+1.5IQR. Outliers are showed as point in the box plots. Statistical analyses were performed using the two-sided Student's t test. **b.** Kaplan-Meier estimates of progression-free survival in patients with TP53-altered tumors versus those with TP53-wildtype (log-rank P=0.056).

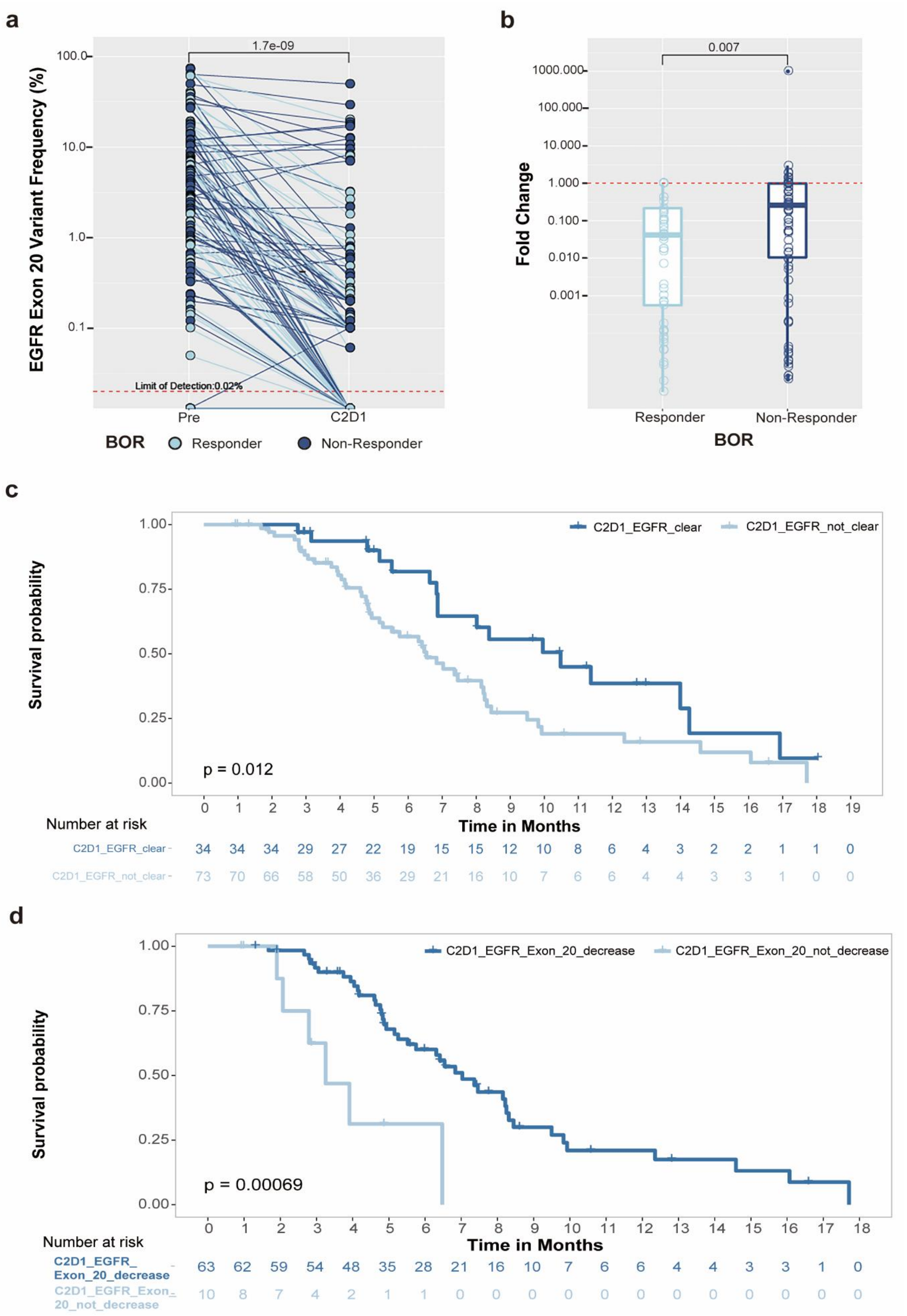
a
1.7e-09
EGFR Exon 20 Variant Frequency (%)
100.0
10.0
1.0
0.1
Limit of Detection:0.02%
Pre
C2D1
BOR
Responder
Non-Responder
b
0.007
1000.000
100.000
10.000
1.000
0.100
0.010
0.001
Fold Change
Responder
Non-Responder
BOR
c
C2D1_EGFR_clear
C2D1_EGFR_not_clear
Survival probability
1.00
0.75
0.50
0.25
0.00
p = 0.012
0 1 2 3 4 5 6 7 8 9 10 11 12 13 14 15 16 17 18 19
Time in Months
Number at risk
C2D1_EGFR_clear 34 34 34 29 27 22 19 15 15 12 10 8 6 4 3 2 2 1 1 0
C2D1_EGFR_not_clear 73 70 66 58 50 36 29 21 16 10 7 6 6 4 4 3 3 1 0 0
d
C2D1_EGFR_Exon_20_decrease
C2D1_EGFR_Exon_20_not_decrease
Survival probability
1.00
0.75
0.50
0.25
0.00
p = 0.00069
0 1 2 3 4 5 6 7 8 9 10 11 12 13 14 15 16 17 18
Time in Months
Number at risk
C2D1_EGFR_ Exon_20_decrease 63 62 59 54 48 35 28 21 16 10 7 6 6 4 4 3 3 1 0
C2D1_EGFR_Exon_ 20_not_decrease 10 8 7 4 2 1 1 0 0 0 0 0 0 0 0 0 0 0 0

Supplementary Figure 10. Changes in plasma variant allelic frequency (VAF) and association with treatment outcomes in patients with EGFR 20ins.

a. Changes in plasma VAF at baseline and at C2D1 (n=135). Patients with IRC-assessed, confirmed PR as the best of response were defined as responders (n=53). Other patients were non-responders (n=82). P-value was determined by two-sided Mann-Whitney U test; **b.** Box-and-whisker plots of fold change in plasma VAF after one cycle of treatment. Each dot represents one patient (Responder = 53, Non-responders = 82). The center line in the box plots represents the median; the upper limit of the box plots represents the 75th percentile (Q3); the lower limit of the box plots represents the 25th percentile (Q1); the upper whisker is the maximum value of Q3+1.5IQR, and the lower whisker is the minimum value of Q1-1.5IQR. The outlier is defined as a value less than Q1-1.5IQR or greater than Q3+1.5IQR. P-value was determined by two-sided Mann-Whitney U test; **c.** Kaplan-Meier curves of progression-free survival in patients with plasma EGFR 20ins VAF clearance at C2D1 (n=34) versus those without (n=73); **d.** Kaplan-Meier curves of progression-free survival in patients with decreased plasma EGFR 20ins VAF at C2D1 (fold change<1, n=63) versus those with unchanged or increased VAF (fold change≥1, n=10). PFS curves were compared using the log-rank test.

Supplementary Table 1. EGFR exon 20 insertions detected in enrolled patients.

Mutation	No. of patients (%) (N = 150)
A767_V769dup	49 (32.7)
S768_D770dup	29 (19.3)
P772_H773dup	10 (6.7)
H773dup	7 (4.7)
N771_H773dup	7 (4.7)
A763_Y764insFQEA	4 (2.7)
H773_V774dup	3 (2.0)
N771delinsGY	3 (2.0)
D770_N771insG	2 (1.3)
H773_V774insHPH	2 (1.3)
D770_N771delDinsGG	1 (0.7)
D770_N771delinsGGT	1 (0.7)
D770_N771insY	1 (0.7)
D770_N771insGF	1 (0.7)
D770_P772dup	1 (0.7)
D770delinsEK	1 (0.7)
D770delinsGY	1 (0.7)
H773_V774insAH	1 (0.7)
H773_V774insANPH	1 (0.7)
H773_V774insPHPH	1 (0.7)
H773_V774insY	1 (0.7)
H773delinsQY	1 (0.7)
H773delinsYNPY	1 (0.7)
N771_P772insG	1 (0.7)
N771_P772insGNN	1 (0.7)
N771_P772insNN	1 (0.7)
N771_P772insT	1 (0.7)
N771delinsAH	1 (0.7)
N771delinsGD	1 (0.7)
N771delinsGF	1 (0.7)
N771delinsKG	1 (0.7)
N771delinsRRH	1 (0.7)
N771delinsSH	1 (0.7)
P772_H773delinsHNPY	1 (0.7)
P772_H773insL	1 (0.7)
S768_V769insLDS	1 (0.7)
Y764_V765insHH	1 (0.7)
Unknown	7 (4.7)

Supplementary Table 2. Incidence of all-grade treatment-related TEAEs≥10% and grade 3 or higher treatment-related TEAEs≥3% in cohort A1, A2 and B2.

TEAE, n (%)	A1 cohort (n = 11)		A2 cohort (n = 6)		B1 cohort (n = 12)	
	All grade	Grade ≥3	All grade	Grade ≥3	All grade	Grade ≥3
Any	11 (100.0)	8 (72.7)	6 (100.0)	1 (16.7)	11 (91.7)	5 (41.7)
Rash	9 (81.8)	4 (36.4)	4 (66.7)	0	9 (75.5)	2 (16.7)
Diarrhea	6 (54.5)	1 (9.1)	5 (83.3)	0	7 (58.3)	1 (8.3)
Dry skin	8 (72.7)	1 (9.1)	5 (83.3)	0	8 (66.7)	1 (8.3)
Decreased appetite	6 (54.5)	1 (9.1)	4 (66.7)	0	8 (66.7)	0
Paronychia	9 (81.8)	0	5 (83.3)	1 (16.7)	3 (25.0)	0
Aspartate aminotransferase increased	8 (72.7)	0	1 (16.7)	0	4 (33.3)	0
Alanine aminotransferase increased	4 (36.4)	0	3 (50.0)	0	3 (25.0)	0
Weight decreased	0	0	0	0	2 (16.7)	0
Vomiting	4 (36.4)	0	1 (16.7)	0	6 (50.0)	0
Oral mucositis	5 (45.5)	1 (9.1)	3 (50.0)	0	2 (16.7)	0
Nausea	4 (36.4)	0	0	0	3 (25.0)	0
Pruritus	4 (36.4)	0	3 (50.0)	0	6 (50.0)	0
Anemia	4 (36.4)	1 (9.1)	0	0	3 (25.0)	0
Malaise	6 (54.5)	0	0	0	4 (33.3)	0
White blood cell decreased	2 (18.2)	0	0	0	2 (16.7)	0
Platelet count decreased	0	0	0	0	1 (8.3)	1 (8.3)
Hypoalbuminemia	3 (27.3)	0	2 (33.3)	0	3 (25.0)	0

Neutrophil count decreased	1 (9.1)	0	0	0	0	0
Fever	5 (45.5)	0	3 (50.0)	0	0	0
Skin hypopigmentation	3 (27.3)	0	2 (33.3)	0	3 (25.0)	0
Dizziness	3 (27.3)	0	1 (16.7)	0	4 (33.3)	0
Hyponatremia	1 (9.1)	0	0	0	3 (25.0)	0
Proteinuria	0	0	0	0	1 (8.3)	0
Stomal ulcer	1 (9.1)	0	0	0	1 (8.3)	0
Hypokalemia	4 (36.3)	0	0	0	7 (58.3)	2 (16.7)
CPK increased	0	0	0	0	0	0
Headache	3 (27.3)	0	2 (33.3)	0	2 (16.7)	0
Skin fissures	0	0	0	0	2 (16.7)	0
QT interval prolonged	0	0	0	0	0	0
Blood LDH increased	0	0	0	0	2 (16.7)	0
Blood creatinine increased	1 (9.1)	0	1 (16.7)	0	1 (8.3)	0
Hypocalcemia	1 (9.1)	0	0	0	0	0

Abbreviations: TEAE, treatment-emergent adverse events; CPK, creatine phosphate kinase.

Supplementary Table 3. Summary of pharmacokinetic parameters of JMT101

	A1 cohort (n = 8)	A2 cohort (n = 5)	B1 cohort (n = 10)	B2 cohort (n = 77)
**PK parameters after single dose**				
$T_{1/2}$ (h)	115 (18.9)	115 (22.6)	115 (19.6)	110 (24.7)
C_{max} (ng/mL)	148250 (25756)	215400 (71101)	145200 (32089)	153655 (73925)
AUC_{0-t} (h*ng/mL)	22638822 (3991585)	26024627 (4251518)	20629211 (4603208)	19399681 (7368327)
AUC_{0-inf} (h*ng/mL)	25881026 (4293541)	29677623 (4363755)	23603219 (4817013)	22004572 (7946652)
V_z (mL/kg)	39.2 (7.83)	34.5 (9.42)	44.5 (12.9)	47.1 (14.6)
CL (mL/h/kg)	0.24 (0.04)	0.21 (0.03)	0.27 (0.07)	0.30 (0.10)
**PK parameters after the third dose**				
	**A1 cohort*** **(n = 2)**	**A2 cohort (n = 3)**	**B1 cohort (n = 5)**	**B2 cohort (n = 37)**
$T_{1/2}$ (h)	151	119 (26.8)	115 (23.3)	116 (25.6)
$C_{max, ss}$ (ng/mL)	242500	253000 (19157)	448800 (555557)	172427 (59836)
AUC_{0-t} (h*ng/mL)	41558650	39769458 (6347694)	26436290 (4085989)	23753637 (10025438)
AUC_{0-inf} (h*ng/mL)	51493964	46017479 (9374443)	30075805 (3597079)	27658535 (12577749)
C_{τ} (ng/mL)	45800	34800 (11628)	21340 (5678)	21424 (13339)
CL_{ss} (mL/h/kg)	0.148	0.153 (0.024)	0.232 (0.037)	0.296 (0.133)
V_{ss_obs} (mL/kg)	29.9	20.9 (4.81)	33.9 (11.3)	41.2 (15.8)

*Data were described by mean (SD) except for A1 cohort after the third dose which showed only the mean values, due to the data from two patients. AUC_{0-t}, Area under the plasma concentration-time curve from time 0 to a definite time t; $AUC_{0-\infty}$, Area under the plasma concentration-time curve from time 0 to infinity; C_{max}, Maximum concentration; $C_{max, ss}$, Maximum concentration in steady-state; C_{τ}, Trough (pre-dose) concentration; CL, Clearance; CL_{ss}, Clearance in steady-state; $T_{1/2}$, Half-time; V_{ss_obs}, Apparent volume of distribution in steady-state.

Supplementary Table 4. Antitumor activity of JMT101 plus afatinib or osimertinib in NSCLC patients with EGFR exon 20 insertions.

Response per RECIST 1.1	A1 cohort (n = 11)	A2 cohort (n = 6)	B1 cohort (n = 12)	B2 cohort (n = 121)
IRC-assessed, No. (%)				
Confirmed ORR (95% CI)	18.2 (2.3-51.8)	33.3 (4.3-77.7)	41.7 (15.2-72.3)	36.4 (27.8-45.6)
Confirmed DCR (95% CI)	90.9 (58.7-99.8)	100.0 (54.1-100.0)	91.7 (61.5-99.8)	95.0 (89.5-98.2)
CR	0	0	0	0
PR	2 (18.2)	2 (33.3)	5 (41.7)	44 (36.4)
SD	8 (72.7)	4 (66.7)	6 (50.0)	71 (58.7)
PD	1 (9.1)	0	0	1 (0.8)
NE	0	0	1 (8.3)	5 (4.1)
Investigator-assessed, No. (%)				
Confirmed ORR (95% CI)	27.3 (6.0-61.0)	33.3 (4.3-77.7)	33.3 (9.9-65.1)	34.7 (26.3-43.9)
Confirmed DCR (95% CI)	90.9 (58.7-99.8)	100.0 (54.1-100.0)	91.7 (61.5-99.8)	95.0 (89.5-98.2)
CR	0	0	0	0
PR	3 (27.3)	2 (33.3)	4 (33.3)	42 (34.7)
SD	7 (63.6)	4 (66.7)	7 (58.3)	73 (60.3)
PD	1 (9.1)	0	0	3 (2.5)
NE	0	0	1 (8.3)	3 (2.5)

Abbreviations: BOR, best overall response; CR, complete response; DCR, disease control rate; NE, not evaluated; ORR, objective response rate; PD, progressive disease; PR, partial response; SD, stable disease.

Supplementary Table 5. Genes and the type of alterations covered in the HapOnco™ 107 panel.

Genes	Type of alterations	Genes	Type of alterations
ABCB1	SNV/Indel	ESR1	SNV/Indel
BMPR1A	SNV/Indel	GREM1	SNV/Indel
CDK6	SNV/Indel	KIT	SNV/Indel
ERBB2	SNV/Indel, CNV	MSH6	SNV/Indel
FOXA1	SNV/Indel	NTRK2	SNV/Indel, rearrangements
JAK2	SNV/Indel	PTCH1	SNV/Indel
MLH1	SNV/Indel	ROS1	SNV/Indel, rearrangements
NRAS	SNV/Indel, CNV	TP53	SNV/Indel
PMS2	SNV/Indel	ALK	SNV/Indel, rearrangements
RET	SNV/Indel, rearrangements	BRIP1	SNV/Indel
SMARCA4	SNV/Indel	CHEK2	SNV/Indel
AKT1	SNV/Indel	FANCA	SNV/Indel
BRAF	SNV/Indel, CNV	HDAC2	SNV/Indel
CDKN1B	SNV/Indel	KRAS	SNV/Indel, CNV
ERBB3	SNV/Indel	MTOR	SNV/Indel
GNA11	SNV/Indel	NTRK3	SNV/Indel, rearrangements
JAK3	SNV/Indel	PTEN	SNV/Indel
MRE11A	SNV/Indel	RRM1	SNV/Indel
NRG1	SNV/Indel	TSC1	SNV/Indel
POLD1	SNV/Indel	APC	SNV/Indel
RICTOR	SNV/Indel	CCND1	SNV/Indel
SMO	SNV/Indel	CTNNB1	SNV/Indel
AKT2	SNV/Indel	FBXW7	SNV/Indel
BRCA1	SNV/Indel	HIF1A	SNV/Indel
CDKN2A	SNV/Indel	MAP2K1	SNV/Indel
ERCC3	SNV/Indel	MUTYH	SNV/Indel
GNAQ	SNV/Indel	PALB2	SNV/Indel
KDR	SNV/Indel	RAD51	SNV/Indel
MSH2	SNV/Indel	SDHA	SNV/Indel
NTRK1	SNV/Indel, rearrangements	TSC2	SNV/Indel
POLE	SNV/Indel	AR	SNV/Indel
RIT1	SNV/Indel	CCND2	SNV/Indel
STK11	SNV/Indel	DDR2	SNV/Indel
AKT3	SNV/Indel	FGFR1	SNV/Indel, CNV, rearrangements
BRCA2	SNV/Indel	HOXB13	SNV/Indel
CHEK1	SNV/Indel	MAP2K2	SNV/Indel
ARAF	SNV/Indel	MYC	SNV/Indel

CCND3	SNV/Indel	PDGFRA	SNV/Indel
DPYD	SNV/Indel	RAD51C	SNV/Indel
FGFR2	SNV/Indel, CNV, rearrangements	SDHB	SNV/Indel
HRAS	SNV/Indel	VEGFA	SNV/Indel
MAPK1	SNV/Indel	CCNE1	SNV/Indel
NBN	SNV/Indel	EGFR	SNV/Indel, CNV
PDGFRB	SNV/Indel	FGFR3	SNV/Indel, CNV
RAD51D	SNV/Indel	IGF1R	SNV/Indel
SDHC	SNV/Indel	MDM2	SNV/Indel
ATM	SNV/Indel	NF1	SNV/Indel
RAF1	SNV/Indel	PGR	SNV/Indel
SDHD	SNV/Indel	NOTCH1	SNV/Indel
BCL2L11	SNV/Indel	PIK3CA	SNV/Indel
CDK4	SNV/Indel	RB1	SNV/Indel
EPCAM	SNV/Indel	SMAD4	SNV/Indel
FLT4	SNV/Indel	JAK1	SNV/Indel
MET	SNV/Indel, CNV		

Supplementary Note

A Phase Ib Clinical Study to Evaluate the Safety and Efficacy of JMT101 in Combination with Afatinib or Osimertinib in Stage IIIB or IV Non-small Cell Lung Cancer Patients with EGFR Exon 20 Insertion Mutations

Protocol No.: JMT101-CSP-001
Version No.: v 3.0
Version date: December 10, 2020
Principal Investigator: Professor Zhang Li
Clinical study site: Sun Yat-sen University Cancer Center
Sponsor: Shanghai JMT-Bio Inc.
Sponsor representative: Yang Xiugao
Tel: 021-60677906

Confidentiality Note

This document contains important confidential business information that is proprietary to Shanghai JMT-Bio Inc. Any disclosure is strictly prohibited unless such disclosure is required by current laws or regulations. Persons to whom the information is disclosed must be informed that it is confidential. This requirement also applies to all documents of a confidential nature that will be provided to you in the future.

Clinical Study Protocol Consent Form - Investigator Signature Page

I, the undersigned, have read this clinical study protocol (protocol number: JMT101-CSP-001, version number: v3.0, version date: December 10, 2020) and agreed to conduct clinical trial in accordance with all the provisions of this protocol, current regulations, and the ethical principles in the Declaration of Helsinki.

Clinical study site: Sun Yat-sen University Cancer Center
Principal Investigator's Signature/Date:

YY MM DD

Clinical Study Protocol Consent Form - Sponsor Signature Page

I, the undersigned, have read this clinical study protocol (protocol number: JMT101-CSP-001, version number: v3.0, version date: December 10, 2020) and agreed to conduct clinical trial in accordance with all the provisions of this protocol, current regulations, and the ethical principles in the Declaration of Helsinki.

Sponsor: Shanghai JMT-Bio Inc.
Signature/date of sponsor's representative:

YY MM DD

____

Other study sites

No.	Site name

List of Abbreviations

**Abbreviations and terms**	**Full name in English**
ADA	Anti-Drug Antibodies
ADCC	Antibody-dependent Cell-mediated Cytotoxicity
AE	Adverse Event
ALB	Albumin
ALT	Alanine Aminotransferase
ALP	Alkaline Phosphatase
ANC	Absolute Neutrophil Count
APTT	Activated Partial Thromboplastin Time
AST	Aspartate Aminotransferase
AUC_{0-t}	Area under the Curve from Zero to the Time Point
AUC_{0-inf}	Area under the Curve from Zero to Infinite
$AUC_{0-\tau}$	Area under the Plasma Concentration-Time Curve of any Interdose at Steady State
BUN	Blood Urea Nitrogen
Ca	Calcium
Ccr	Creatinine Clearance Rate
CDC	Complement - dependent Cytotoxicity
CK	Creatine Kinase
Cl	Chlorine
CL	Clearance of Drug
Cr	Creatinine
CRF	Case Report Form
CTCAE	Common Terminology Criteria for Adverse Events
$C_{av,ss}$	Steady-State Average Concentration
C_{max}	Maximum Concentration
$C_{max,ss}$	Steady-State Maximum Concentration
$C_{min,ss}$	Steady-State Minimum Concentration
CYP3A4	Cytochrome P450 3A4
DBil	Direct Bilirubin
DCR	Disease Control Rate
DLT	Dose Limited Toxicity
DNA	Deoxyribonucleic aAcid
DF	Degree of Fluctuation
DOR	Duration of Response
EDC	Electronic Data Capture
EGFR	Epidermal Growth Factor Receptor
FAS	Full Analysis Set
FBG	Fibrinogen
FDA	The Food and Drug Administration
GCP	Good Clinical Practice
GGT	Glutamyltransferase
Glu	Glucose
h	Hour
Hb/HGB	Hemoglobin
HIV	Human Immunodeficiency Virus

HNSTD	The Highest Nonseverely Toxic Dose
ICH	The International Council for Harmonisation of Technical Requirements for Pharmaceuticals for Human Use
INR	International Normalized Ratio
IHC	Immunohistochemistry
IRB	Institutional Review Board
IRC	Independent Review Committee
ITT	Untend to Treat
K	Potassium
KET	Ketone Body
LDH	Lactic Dehydrogenase
LVEF	Left Ventricular Ejection Fraction
Mg	Magnesium
Min	Minute
MTD	Maximum Tolerated Dose
Na	Sodium
NAbs	Neutralizing Antibodies
NCCN	The National Comprehensive Cancer Network
NGS	Next-generation Sequencing
NMPA	National Medical Products Administration
NOAEL	No Observable Adverse Effect Level
NYHA	New York Heart Association
ORR	Objective Response Rate
OS	Overall Survival
P	Phosphorus
PD	Progressive Disease
PFS	Progression Free Survival
P-gp	P-glycoprotein
PLT	Platelet
PK	Pharmacokinetics
PR	Partial Response
PT	Prothrombin Time
qPCR	Quantitative Polymerase Chain Reaction
RANKL	Receptor Activator for Nuclear Factor- κ B Ligand
RBC	Red Blood Cell
RECIST1.1	Response Evaluation Criteriain Solid Tumors
RNA	Ribonucleic Acid
RP2D	Recommended Phase 2 Dose
SAE	Serious Adverse Event
SD	Stable Disease
SOC	Systematic Organ Classification
SOP	Standard Operation Procedure
SS	Safety Analysis Set
TBil	Total Bilirubin
TC	Total Cholesterol
TEAE	Treatment Emergent Adverse Event
TG	Triglyceride
TP	Total Protein

TT	Thrombin Time
$t_{1/2}$	Half-Time
T_{max}	Time to Maximum Plasma Concentration
$T_{max,ss}$	Steady-State Time to Maximum Plasma Concentration
V_d	Volume of Distribution
$\%AUC_{ext}$	% of the Area under the Curve that has been Derived after Extrapolation
ULN	Upper limit of Normal
WBC	White Blood Cell

Table of Contents

1 SYNOPSIS 7
1.1 Summary 7
1.2 Study Process Schema 19
1.3 Schedule of Activities (SOA) 20
2 Introduction 28
2.1 Study Rationale 28
2.1.1 Epidermal growth factor receptor 28
2.1.2 Epidemiological characteristics of NSCLC with EGFR exon 20 insertion mutations 28
2.1.3 Unmet clinical needs of NSCLC with EGFR exon 20 insertion mutations 29
2.2 Background 30
2.2.1 Investigational drug 30
2.2.2 Overview of Pre-clinical Study 31
2.2.3 Clinical Studies 32
2.2.4 Overview of Similar Drug Studies 33
2.2.5 Known Potential Risks 34
2.2.6 Known Potential Benefits 34
2.2.7 Assessment of Potential Risks and Benefits 35
3 Study objectives and endpoints 35
4 Trial design 35
4.1 Overall Design 35
4.2 Definition of Dose-Limiting Toxicity 37
4.3 Definition of Maximum Tolerated Dose 38
4.4 Definition of End of Study 38
5 Study population 38
5.1 Inclusion Criteria 38
5.2 Exclusion Criteria 39
5.3 Screening Failure 40
6 Study intervention 40
6.1 Investigational drug 40
6.1.1 Investigational Drug Description 40
6.1.2 Dose Regimen of Investigational Drug 41
6.1.3 Principles of dose modification and toxicity treatment 42
6.2 Preparation/Handling/Storage/Disposal of Investigational Drug 43
6.2.1 Drug receipt and accountability 43
6.2.2 Dosage form, appearance, packaging and labeling 44
6.2.3 Product storage and stability 44
6.2.4 Preparation 44
6.3 Pharmacokinetics, immunogenicity, biomarker test 44
6.3.1 Pharmacokinetic test 44
6.3.2 Immunogenicity detection 44
6.3.3 Biomarker detection 45
6.4 Methods to reduce deviations: randomization and blinding 45
6.5 Study intervention compliance 45
6.6 Concomitant Medication and Treatment 45
6.6.1 Prohibited drugs and treatments 45
6.6.2 Permitted Drugs and Treatments 45
7 Discontinuation of Study Intervention and Subject Withdrawal 46
7.1 Discontinuation of Study Intervention 46

7.2 Subject Withdrawal 46
7.3 Lost to follow-up 47
8 Study evaluation and procedure 47
8.1 Demographic Characteristics and Background Evaluation 47
8.1.1 Demographic characteristics 47
8.1.2 Body weight and height 47
8.1.3 Medical history and other past medical histories 47
8.1.4 Prior medication/concomitant medication and therapy 48
8.1.5 Prior anti-tumor therapy 48
8.1.6 Serum virology and syphilis 48
8.1.7 Tumor assessment 48
8.2 Assessment on Safety and Other Items 49
8.2.1 Laboratory test items 49
8.2.2 Laboratory test time and evaluation 49
8.2.3 Physical examination 49
8.2.4 Vital sign 49
8.2.5 Performance status 50
8.2.6 ECG examination 50
8.2.7 Cardiac color ultrasonography 50
8.3 Efficacy Assessment 50
8.3.1 Endpoints 50
8.3.2 Efficacy criteria 50
8.4 Pharmacokinetic, immunogenicity and biomarker evaluation 51
8.4.1 Pharmacokinetic parameters 51
8.4.2 Immunogenicity parameters 51
8.4.3 Biomarker parameters 51
8.5 Adverse Events and Serious Adverse Events 51
8.5.1 Definition 51
8.5.2 Adverse event evaluation and classification 52
8.5.3 Causal relationship 53
8.5.4 Adverse event collection, recording and evaluation 55
8.5.5 Serious adverse event reporting 56
8.5.6 Pregnancy 56
9 Statistical Considerations 57
9.1 Statistical Hypotheses 57
9.2 Sample Size Determination 57
9.3 Analysis Population 57
9.4 Statistical Analysis 58
9.4.1 General method 58
9.4.2 Subject disposition 58
9.4.3 Baseline descriptive statistics 58
9.4.4 Pharmacokinetic, immunogenicity and biomarker analysis 58
9.4.5 Safety analysis 58
9.4.6 Efficacy analysis 59
9.4.7 Planned interim analysis 60
9.4.8 Sub-group analyses 60
9.4.9 Exploratory analyses 60
10 Supporting Documentation and Operation Considerations 60
10.1 Regulatory, Ethical, and Study Oversight Considerations 60
10.1.1 Informed consent process 60

10.1.2 Study suspension and termination 60
10.1.3 Confidentiality and privacy 61
10.1.4 Future use of stored specimens and data 61
10.1.5 Key role and study governance 62
10.1.6 Safety surveillance 62
10.1.7 Quality control and quality assurance 62
10.1.8 Clinical study monitor 62
10.1.9 Data processing and record retention 63
10.1.10 Protocol deviations 63
10.1.11 Study publication and data sharing policy 64
10.2 Historical Record of Protocol Modifications 65
11 References 66
Appendix 70
Appendix - ECOG Performance Status 70
Appendix II Heart Function Grade of New York Heart Association (NYHA) 70
Appendix III Response Evaluation Criteria in Solid Tumors (RECIST 1.1) Excerpts 70
Appendix IV Strong and Moderate Inducers of CYP3A4 and Strong Inducer and Inhibitor of P-gp 82
Appendix V Revision 7 of International TNM Classification and Staging of Lung Cancer 82

1 SYNOPSIS

1.1 Summary

<table>
<tr><td>Protocol No.</td><td colspan="2">JMT101-CSP-001</td></tr>
<tr><td>Protocol name</td><td colspan="2">A Phase Ib Clinical Study to Evaluate the Safety and Efficacy of JMT101 in Combination with Afatinib or Osimertinib in Stage IIIB or IV Non-small Cell Lung Cancer Patients with EGFR Exon 20 insertion Mutations</td></tr>
<tr><td>Version No./date</td><td colspan="2">V3.0, December 10, 2020</td></tr>
<tr><td>Trial staging</td><td colspan="2">Phase Ib</td></tr>
<tr><td>Registration classification</td><td colspan="2">Class 2 biological products</td></tr>
<tr><td>Sponsor</td><td colspan="2">Company: Shanghai JMT-Bio Inc.
Study director: Yang Xiugao
Tel.: 021-60677906</td></tr>
<tr><td>Principal investigator</td><td colspan="2">Professor Zhang Li</td></tr>
<tr><td>Clinical study site</td><td colspan="2">Sun Yat-sen University Cancer Center</td></tr>
<tr><td rowspan="6">Study objectives and endpoints</td><td><u>Study Objectives</u></td><td><u>Endpoints</u></td></tr>
<tr><td>Primary objective:
• To evaluate the safety and tolerability of JMT101 in combination with Afatinib or Osimertinib in stage IIIB or IV non-small cell lung cancer (NSCLC) patients with EGFR exon 20 insertion mutations.</td><td>Primary endpoint:
• Adverse events, physical examination, vital signs, laboratory tests (including routine blood, blood biochemistry, routine urine, coagulation), and ECG.</td></tr>
<tr><td>Secondary objectives:
• To evaluate the efficacy of JMT101 in combination with Afatinib or Osimertinib in stage IIIB or IV NSCLC patients with EGFR exon 20 insertion mutations, so as to provide a basis for the recommended dosing regimen for subsequent studies.</td><td>Secondary endpoints:
• Efficacy endpoints: objective response rate (ORR, assessed by IRC and investigators according to RECIST 1.1 criteria, respectively), duration of response (DOR), disease control rate (DCR), progression-free survival (PFS), and overall survival (OS).</td></tr>
<tr><td>• To evaluate the pharmacokinetic profile of JMT101.</td><td>• Pharmacokinetic parameters: including but not limited to AUC_{0-t}, AUC_{0-inf}, C_{max}, T_{max}, t½ and CL.</td></tr>
<tr><td>• To evaluate the immunogenicity of JMT101.</td><td>• Occurrence of anti-drug antibodies and neutralizing antibodies.</td></tr>
<tr><td>• To analyze possible correlations between biomarkers and clinical outcomes</td><td>• Tumor-associated biomarkers are explored to analyze possible correlations between biomarkers and clinical outcomes.</td></tr>
</table>

Trial design	This is a multi-center, open-label, dose-escalation phase Ib clinical study in stage IIIB or IV NSCLC patients, which aims to evaluate the safety, tolerability, and efficacy of JMT101 in combination with Afatinib or Osimertinib in patients with stage IIIB or IV NSCLC harboring EGFR exon 20 insertion mutations, to provide a basis for the recommended dosing regimen for subsequent studies, to evaluate the pharmacokinetic profile and immunogenicity of JMT101, and to explore tumor-associated biomarkers.<br>This study is divided into two stages: the first stage (Stage I) is a dose escalation study and the second stage (Stage II) is a dose expansion study.<br>**Stage I dose escalation study:**<br>This study adopts a combination therapy regimen, which divides the patients into Group A and Group B according to different combined drugs: Group A is treated with JMT101 in combination with Afatinib and Group B is treated with JMT101 in combination with Osimertinib. Each group is divided into two cohorts according to the administered dose, and a total of 4 cohorts are established as follows:<br>A1 cohort: JMT101 6mg/kg, intravenous drip, Q2W + Afatinib 30mg, oral, QD;<br>A2 cohort: JMT101 6mg/kg, intravenous drip, Q2W + Afatinib 40mg, oral, QD;<br>B1 cohort: JMT101 6mg/kg, intravenous drip, Q2W + Osimertinib 80mg, oral, QD;<br>B2 cohort: JMT101 6mg/kg, intravenous drip, Q2W + Osimertinib 160mg, oral, QD;<br>The dose escalation follows the “3 + 3 principle”. Each cohort includes 3~6 subjects to observe safety and tolerability. Subjects with asymptomatic central nervous system (CNS) metastasis or meningeal metastasis are enrolled in Group A or B at the discretion of the investigator, and the rest of the subjects are alternately enrolled into Group A and B in sequence.<br>Subjects are first enrolled in A1 and B1 cohorts, and each of the cohorts shall have at least 3 evaluable subjects. Subjects can be enrolled in higher dose cohorts (A2 or B2) only if no dose-limiting toxicity (DLT) is observed in 3 subjects in cohorts A1 or B1 during the DLT observation period (Cycle 1, Day 1 to 28). If DLT occurs in 1 of 3 subjects in one cohort, 3 additional subjects need to be added to the same cohort (the cohort has 6 evaluable subjects). If no DLT occurs in the 3 additional subjects, the subjects will continue to be enrolled in the higher dose cohort; if DLT occurs in 1 or more of the 3 additional subjects or in 2 or more of the 6 subjects in total, the planned higher dose cohort enrollment will not be conducted. If DLT occurs in 2 of 3 subjects in one cohort, the planned higher dose cohort enrollment will not be conducted.<br>If drug intolerance occurs in the initial dose cohort (A1 or B1) in a group, the investigator and sponsor are required to discuss jointly to decide whether to proceed with a cohort that is one dose level down from JMT101 (i.e., JMT101 4 mg/kg Q2W, with no change in combined drug dose) or to stay in the initial dose cohort and continue enrolling subjects

	until excessive toxicity occurs or early discontinuation of the study in the group. If the safety and tolerability of this dose group remains good when the dose is escalated to the predetermined maximum dose (cohort A2 or B2), the decision to explore JMT101 up one dose level cohort (i.e. JMT101 8 mg/kg Q2W with no change in the combination drug dose) may be discussed between the investigator and the sponsor.<br>The previous dose level of the dose limiting toxicity (DLT) dose level is defined as the maximum tolerated dose (MTD). At least 6 subjects are enrolled and evaluated in the MTD dose group.<br>**Stage II dose expansion study:**<br>According to the safety, tolerability and efficacy data obtained in Stage I, the dose expansion study is carried out for the target dose cohort. All cohorts with good safety and tolerability in Stage I (up to 4 cohorts) are selected in which a certain number of additional subjects are included to further explore safety, tolerability, pharmacokinetic profile, and anti-tumor activity. Subjects with asymptomatic central nervous system (CNS) metastasis or meningeal metastasis are enrolled in Group A or B at the discretion of the investigator, and the rest of the subjects are enrolled into each cohort in a certain order. Safety and efficacy data are monitored periodically during the course of the study. Considering the subject benefits, if a cohort shows clear evidence of treatment disadvantage, the enrollment into the cohort should be closed in advance to avoid more subjects receiving ineffective or low effective treatment; if a cohort shows clear evidence of treatment benefits, the recruiting number may be increased in that cohort. A minimum of 12 subjects and a maximum of 200 subjects are expected to be enrolled in the dose escalation study and the dose expansion study.<br>For all the above subjects, every 4 weeks (28 days) is considered as a treatment cycle during the treatment period, and treatment continues until one of the following occurs: progressive disease, unacceptable toxicity, subject’s withdrawal request, or absence of further benefit from treatment judged by the investigator, whichever occurs first. The dosing regimen may be adjusted based on the subject’s toxicity reaction (the dosing regimen are not allowed to be adjusted for the subjects during the DLT observation period in Stage I), refer to section 6.1.3 of the protocol for specific dose modification principles.<br>Safety inspections are performed during treatment as required by the protocol, and tumor assessments are performed at the end of Cycle 1 and the end of every two cycles thereafter (Cycle 3, Cycle 5 ....) (If tumor-associated symptoms worsen and the investigator deems it necessary, the duration of tumor assessment may be shortened). After the last dose of investigational drug, subjects should be followed up for survival every 8 weeks, and for adverse events still present at the end-of-study visit.
Dose-limiting toxicity (DLT)	DLT is defined as: one or more of the following toxic reactions judged to be reasonably related (related, probably related, and possibly related) to the investigational drug that occurs within Cycle 1 (Day 1 to 28) of study dosing.<br>**(1) Non-hematologic toxicity:**<br>• Grade 4 rash, or Grade 3 rash that leads to 4 weeks of suspension, or

	Grade 3 rash with severe infection.<br>• Grade 4 diarrhea, or Grade 3 diarrhea that has not resolved after 2 weeks of suspension, or Grade 3 diarrhea that reappears after appropriate supportive therapy.<br>• Any non-hematologic toxicity of grade 3 or greater other than rash and diarrhea (nausea/vomiting, constipation and electrolyte imbalance are considered as DLT only if they remain ⩾ grade 3 after appropriate supportive treatment).<br>• Development of interstitial pneumonia or pulmonary fibrosis.<br>**(2) Hematologic toxicity:**<br>• Grade 4 neutropenia that persists for more than 5 days.<br>• Febrile neutropenia (ANC < 1.0 × 10^9/L with temperature of 38.3℃ (axillary temperature) in a single measurement or temperature ⩾ 38℃ (axillary temperature) for more than one hour).<br>• Grade 3 neutropenia with evidence of infection.<br>• Grade 3 thrombocytopenia with clinically significant hemorrhage.<br>• Grade 4 thrombocytopenia.<br>• Grade 4 anaemia (life threatening).<br>**(3) Other toxic reactions that, in the judgment of the investigator, should result in permanent discontinuation of the investigational drug.**<br>All adverse events will be graded according to the National Cancer Institute Common Terminology Criteria for Adverse Events (NCI-CTCAE) version 5.0.
Trial population	**Inclusion criteria:**<br>1. At age of ≥18 years old, regardless of gender.<br>2. Naive or treated patients who have a histologically or cytologically confirmed diagnosis of stage IIIB or IV NSCLC and are not suitable for radical surgery or radiotherapy, with confirmed EGFR exon 20 insertion mutations (including duplication mutations).<br>*NSCLC staging refers to the 7th Edition of TNM Classification for Lung Cancer (revised). see Appendix V for details.<br>3. The presence of at least one measurable lesion at baseline defined by RECIST 1.1 criteria.<br>4. ECOG performance status score: 0 or 1.<br>5. Estimated survival time ≥ 3 months.<br>6. Major organ and bone marrow function within 7 days prior to treatment meets the following criteria (no transfusion, EPO, G-CSF, GM-CSF, or other supportive therapies within 7 days prior to investigational drug administration).

System	Laboratory test value
**Routine blood**	
Absolute neutrophil count (ANC)	⩾1.5×10^9 /L
Platelet	⩾90×10^9 /L
Haematoglobin	⩾90 g/L or ⩾5.6 mmol/L
**Kidney**	
Serum creatinine	⩽1.5 × upper limit of normal (ULN)
**Liver**	
Total bilirubin	⩽1.5×ULN

	Patients with liver metastasis: ≤ 3 × ULN
AST and ALT	≤2.5 × ULN<br>Patients with liver metastasis: ≤ 5×ULN
**Coagulation**	
International normalized ratio (INR) or prothrombin time (PT)	≤1.5×ULN
Activated partial thromboplastin time (APTT)	≤1.5×ULN

7. Females of childbearing potential must have a negative result upon blood pregnancy test within 7 days prior to administration of investigational drug; any male and female patients of childbearing potential must agree to use effective contraceptive methods during the entire trial period and within six months after trial completion. A patient of childbearing potential, in the judgment of the investigator, refers to one who is biologically capable of giving birth to a child as well as having a normal sexual life. Female patients of no childbearing potential (i.e., at least 1 of the following criteria is met):

- Having undergone hysterectomy or bilateral oophorectomy, or
- Medically confirmed as ovarian failure, or medically confirmed as post-menopause (at least 12 consecutive months of menopause without pathological or physiological causes).

8. Subjects are required to give informed consent to the study and voluntarily sign a written informed consent form prior to the trial.

Exclusion criteria:

1. Previously treated with EGFR monoclonal antibody targeted therapy.
2. Having received anti-tumor therapies such as chemotherapy, biologic therapy, targeted therapy, or immunotherapy within 4 weeks prior to first dose of investigational drug, oral small molecule targeted drugs, within 2 weeks prior to first dose of investigational drug or within 5 half-lives of the known drug (whichever is longer); for radiotherapy, within 2 weeks prior to first dose of investigational drug.
3. Treated with another clinical investigational drug within 4 weeks prior to first dose of investigational drug.
4. Major organ surgery (excluding aspiration biopsy) or significant trauma within 4 weeks prior to first dose of investigational drug.
5. Known hypersensitivity reactions or intolerances to any component of the investigational drug or its excipients.
6. Use of strong or moderate inducers of CYP3A4, strong inducers and inhibitors of P-gp within 14 days prior to first dose of investigational drug. See Appendix IV for details.
7. Adverse effects due to prior anti-tumor therapy that have not returned to a CTCAE 5.0 grade of ≤1 (except for toxicities such as alopecia, which in the judgment of the investigator are of no safety risk).
8. CNS metastasis or meningeal metastasis with clinical signs.
9. History of autoimmune disease, immunodeficiency, including positive HIV, or other acquired or congenital immunodeficiency diseases, or history of organ transplantation.
10. Active hepatitis B (hepatitis B virus titer > 1000 copies/mL or 200

	IU/mL); hepatitis C virus, syphilis infection.<br>11. History of serious cardiovascular diseases, including but not limited to:<br>• Having complete left bundle branch block or third-degree atrioventricular block.<br>• History of myocardial infarction, angioplasty, coronary artery bypass.<br>• Patients with prolonged QT/QTc interval on the ECG at baseline (QTcF > 450 ms for males and > 480 ms for females).<br>• Severe cardiac arrhythmias that, in the judgment of the investigator, has an impact on this trial.<br>• Left ventricular ejection fraction (LVEF) ≤50% determined by echocardiography (ECHO) or multi-gated acquisition (MUGA) technique at baseline.<br>• New York Heart Association (NYHA) Class II or above cardiac failure.<br>• Poorly controlled hypertension (BP greater than or equal to 150/95 mmHg despite optimal treatment).<br>• Previous or current cardiomyopathy that, in the judgment of the investigator, has an impact on this trial.<br>12. Inability to swallow the drug orally, or presence of a condition that, in the judgment of the investigator, severely impairs gastrointestinal absorption.<br>13. Other malignancies diagnosed within 5 years prior to first dose of investigational drug, except effectively treated skin basal cell carcinoma, skin squamous cell carcinoma and/or effectively resected in situ cervical cancer and/or breast cancer.<br>14. Any previous history of interstitial lung disease, drug-induced interstitial lung disease, radiation pneumonia requiring steroid therapy, or any evidence of clinically active interstitial lung disease.<br>15. History of other serious systemic diseases who, in the judgment of the investigator, are not suitable for participation in the clinical trial.<br>16. Known alcohol or drug dependence.<br>17. Previous history of definite neurological or psychiatric disorders, including epilepsy or dementia.<br>18. Pregnant or lactating women.<br>19. Not suitable to participate in this clinical study for other reasons, in the opinion of the investigator.<br>20. Known to carry EGFR exon 20 insertion mutations along with other EGFR mutations corresponding to approved EGFR-TKI therapeutics (i.e., exon 19 del, L858R, T790M, L861Q, G719X or S768I, where X is any other amino acid).<br>21. Prior use of proposed drugs for NSCLC with EGFR exon 20 insertion mutations such as TAK-788, poziotinib or JNJ-61186372; patients with previous effective prior EGFR-TKI therapy (including optimal efficacy CR, PR, or SD for more than 6 months); patients in the combined Osimertinib cohort who have used immunotherapy agents such as PD-(L)1 monoclonal antibody, etc. within the previous 3 months.
Investigational drug	1. Drug name: Recombinant Humanized Anti-epidermal Growth Factor Receptor (EGFR) Monoclonal Antibody Injection, abbreviated as

	JMT101; Strength: 100 mg/10.0 mL/vial.<br>2. Drug Name: Afatinib Dimaleate Tablets; Trade Name: Giotrif; Strength: 30 mg, 40 mg.<br>3. Drug Name: Osimertinib Mesylate Tablets; Trade Name: TAGRISSO; Strength: 80 mg.
Study intervention	**Method of administration:**<br>JMT101: The administered dose is calculated according to 6 mg/kg based on the body weight before the 1st dose (the error from the theory is allowed ≤±5%). If the subject's body weight in subsequent treatments does not exceed 10% compared with the weight before the 1st dose, the dose will not be recalculated. The route of administration is intravenous drip, and the frequency of administration is every 2 weeks (Q2W). The required amount of this product is drawn accurately, diluted to 100 mL with 0.9% sodium chloride injection, mixed well and administered intravenously for 90 min (±15 min) at a constant rate. If the first dose is tolerable, the infusion time can be shortened to 60 min (60-105 min) in subsequent treatments; the maximum infusion rate should not exceed 10 mg/min. The infusion line is rinsed with 0.9% sterile sodium chloride solution at the end of infusion. This product is not allowed for intravenous bolus or rapid injection. If the drug cannot be used in time after preparation, it should be left at room temperature for no more than 4 h; and at 2-8°C for up to 24 h. Dose modification may be made according to the protocol based on the subject's toxicity reaction.<br>Afatinib: 30 mg or 40 mg, oral, once daily (QD). This product should not be taken with food. It should be taken at least 3 h after eating or at least 1 h before eating. The tablet should be swallowed in whole with water. Dose modification may be made according to the protocol based on the subject's toxicity reaction.<br>Osimertinib: 80 mg or 160 mg, oral, once daily (QD). If one dose is missed, the missed dose should be taken unless the next dose is within 12 h. This product should be taken at the same time each day as possible, either with a meal or on an empty stomach. Dose modification may be made according to the protocol based on the subject's toxicity reaction.<br><br>**Dosing regimen:**<br>• A1 cohort: JMT101 6 mg/kg, intravenous drip, Q2W + Afatinib 30 mg, oral, QD;<br>• A2 cohort: JMT101 6 mg/kg, intravenous drip, Q2W + Afatinib 40 mg, oral, QD;<br>• B1 cohort: JMT101 6 mg/kg, intravenous drip, Q2W + Osimertinib 80 mg, oral, QD;<br>• B2 cohort: JMT101 6 mg/kg, intravenous drip, Q2W + Osimertinib 160 mg, oral, QD.<br><br>Every 4 weeks (28 days) is considered a treatment cycle during the treatment period, and treatment continues until one of the following occurs: progressive disease, unacceptable toxicity, withdrawal of consent, or absence of further benefits in the opinion of the investigator, whichever occurs first. The dosing regimen may be adjusted based on the subject's toxicity reaction (the dosing regimen are not allowed to be

	adjusted for the subjects during the DLT observation period in Stage I). Refer to Section 6.1.3 for specific dose modification principles.
Pharmacokinetic test	**Stage I dose escalation study:**<br>Approximately 2.5 mL of blood will be collected within 30 minutes before the 1st and 3rd dose and immediately after the end of the dose (+2 min), at 4 h (±15 min), 8 h (±30 min), 24 h (±1 h), 48 (±2 h), 96 h (±4 h), 168 h (±7 h), and 240 h (±12 h) after the 1st and 3rd dose.<br>Approximately 2.5 mL of blood will be collected within 30 minutes before and immediately after the 2nd, 4th, and 5th doses (+2 min) and 30 days (±3 days) after the last dose.<br>**Stage II dose expansion study:**<br>Approximately 2.5 mL of blood will be collected within 30 minutes before the 1st and 3rd dose and immediately after the end of the dose (+2 min), at 8 h (±30 min), 24 h (±1 h), and 168 h (±7 h) after the 1st and 3rd dose.<br>Approximately 2.5 mL of blood will be collected each within 30 minutes before and immediately after the 2nd, 4th, and 5th doses (+2 min) and 30 days (±3 days) after the last dose.
Immunogenicity detection	Before the 1st dose, 15 days (±1 day) (before the 2nd dose), 29 days (±1 day) (before the 3rd dose), 57 days (±3 days) (before the 5th dose) after the 1st dose, and at the last visit (30±3 days after the last dose). Blood will be collected within 30 minutes prior to each dose, approximately 3.5 mL per collection.<br>Samples will be first tested for anti-drug antibodies. Samples that are tested positive for anti-drug antibodies need to be further tested for neutralizing antibodies.
Biomarker detection	**Diagnostic biomarker detection:**<br>For EGFR exon 20 insertion mutation detection, one of the following conditions should be met:<br>(1) For those who cannot provide the test report of EGFR exon 20 insertion mutation, NGS or qPCR should be used to detect EGFR exon 20 insertion mutations in tumor tissue DNA or peripheral blood specimens of patients before enrollment. Patients with verified mutations will be enrolled.<br>(2) For those who can provide the test report of EGFR exon 20 insertion mutation, they may be enrolled only after the investigator has reviewed the third-party test report and confirmed the mutation.<br>**Pharmacodynamic biomarker detection:**<br>Ten (10) mL of blood will be collected before the first dose (within 30 min), at the time of first efficacy assessment and disease progression, respectively, and plasma free DNA are extracted and analyzed for the correlation between efficacy and biomarkers using NGS.
Sample Size	Based on the defined cohort: Stage I: The dose escalation study is expected to enroll a minimum of 12 subjects. Stage II: According to the existing study results, the expected ORR of the combination therapy cohort is about 40%, and when the sample size is 90 subjects, the probability of observed ORR>35% calculated by normal approximation method is 83.4%; if the expected ORR is lower than 40%, the probability of observed ORR>35% shows a decreasing trend. Together with subjects in the dose escalation stage, the total number of subjects enrolled in all

	the cohorts is not more than 200. Safety and efficacy data are monitored periodically during the course of the study. Considering the benefits to the subjects, if a cohort shows clear evidence of treatment disadvantage, the cohort will be closed early to avoid more subjects receiving ineffective or low effective treatment; if a cohort shows clear evidence of treatment benefits, an increase in the number of subjects enrolled in that cohort may be considered.<br>A minimum of 12 subjects and a maximum of 200 subjects are expected to be enrolled in the whole study.
Statistical analysis	**Analysis population**<br>Full analysis set (FAS): a collection of all subjects that have been successfully enrolled and used at least one dose of the investigational drug.<br>Per-protocol set (PPS): a collection of subjects that meet the inclusion criteria, do not meet the exclusion criteria, and complete the treatment regime, the set of all subjects who meet the study protocol, have good compliance, complete the treatment specified in the protocol, and have no major protocol deviation in the process will be analyzed.<br>Safety analysis set (SS): All enrolled subjects who have used at least one dose of the investigational drug and have post-dosing safety records will be included in the safety analysis set.<br>DLT Analysis Set (DLT set): The DLT set will include all subjects in the DLT assessment period (1 to 28 d), subjects who complete the DLT assessment or those who withdraw from the trial early due to adverse events during the DLT assessment period (subjects whose DLT is not evaluable due to intolerance to non-investigational drug dose will not be included in the DLT statistical sample). This analysis set will be used to analyze and summarize DLT events.<br>PK concentration set (PKS): Subjects who have received at least one dose of investigational drug and have at least one measured plasma concentration of investigational drug during the trial.<br>PK parameter set (PKPS): Subjects who have received at least one dose of investigational drug and have at least one valid calculation of PK parameter of the investigational drug.<br>Immunogenicity analysis set: Subjects who have received at least one dose of investigational drug and have at least one sample of anti-drug antibodies.<br>Biomarker analysis set: Subjects who have received at least one dose of investigational drug and have at least one tumor tissue sample or one biomarker blood sample collected.<br>**Statistical analysis methods**<br>■ General statistical principles<br>The statistical description of categorical variables is expressed as number of cases and percentage (%). The statistical description of continuous variables is described by number of cases, missing number, mean, standard deviation, median, lower quartile, upper quartile, minimum value, and maximum value. Unless otherwise specified, hypothesis testing will be performed using a two-sided test with a significant level of 0.05, and 95% CI will be used for the estimation of confidence intervals for parameters.

	■ Safety Analysis<br>• Adverse events and adverse reactions:<br>Adverse events and adverse reactions during dosing will be analyzed to summarize the number of cases, frequency and percentage of adverse events, adverse reactions, trial discontinuation due to adverse events, death due to adverse events, serious adverse events, and DLT events in each group of subjects; group comparisons of incidence will be performed when necessary.<br>Adverse events and adverse reactions are coded by System Organ Class (SOC) and Preferred Term (PT) according to MedDRA, and the number of cases, frequency and percentage of adverse events and adverse reactions will be counted according to SOC/PT:<br>The number of cases, frequency and percentage of adverse events and adverse reactions will be counted by SOC/PT and severity. A class of adverse events in a subject is counted only once under the highest severity within the same term (SOC or PT).<br>All adverse events (including those occurring during non-dosing periods), adverse reactions, serious adverse events, study discontinuation due to adverse events, death due to adverse events, adverse events of special interest, and DLT events wil be tabulated separately.<br>• Laboratory test:<br>The statistical time points for each laboratory test item include baseline, each post-baseline visit and the last visit. The measured values at each time point, the minimum and maximum values of post-baseline measurements and observed values at the last visit, as well as their changes from baseline will be counted by dose group and total, respectively.<br>The normal or abnormal changes of each parameter before and after administration are compared using a cross tabulation of clinical judgment.<br>A detailed list of each laboratory test is presented by group.<br>• Vital sign:<br>The statistical time points for vital sign examination items include baseline, each post-baseline visit, and early withdrawal visit. The descriptive statistics results at baseline, post-baseline visit and at the end of the study are counted by group and total. The normal or abnormal changes of each parameter before and after administration are compared using a cross tabulation of clinical judgment (if applicable).<br>• Physical examination and ECG:<br>Physical examination items: general condition, skin and mucosa, lymph node, head and neck, chest, abdomen, spine, muscles and skeletons, nervous system and other parts. The normal or abnormal changes of each dose group before and after administration are compared using a cross tabulation of clinical judgment.<br>ECG examination items include: heart rate, PR, QRS, QTc. The minimum, maximum and last visit observations and their change from baseline values will be statistically analyzed by dose group and total for each visit time point at baseline, post-baseline measurements. The normal or abnormal changes of each dose group before and after administration are compared using a cross tabulation of clinical judgment.

	A detailed list of physical examination and ECG is presented by group.<br><br>■ Efficacy analysis:<br>Objective response rate (ORR: complete response (CR) rate + partial response (PR) rate, assessed by IRC and investigators respectively), duration of response (DOR), disease control rate (DCR: complete response (CR) rate + partial response (PR) rate + stable disease (SD) rate), progression-free survival (PFS), and overall survival (OS) will be assessed according to RECIST v1.1 criteria and relevant evaluation results.<br>The number of cases, percentage and 95% confidence interval (CI) of ORR and DCR are calculated for each dose group versus all subjects.<br>The Kaplan-Meier method is used to estimate the median DOR, median PFS, median OS and their 95% CIs for all subjects and each dose group, and survival curves will be plotted.<br><br>■ PK analysis:<br>(1) Stage I dose escalation study<br>Data from the PK parameter analysis set are analyzed using a non-compartment model to calculate PK parameters for each subject, including C_{max}, AUC_{0-t}, AUC_{0-inf}, T_{max}, V_z, $t_{1/2}$, CL, $\%AUC_{ext}$ after the first dose, and $C_{max,ss}$, $C_{min,ss}$, $AUC_{0-\tau}$, AUC_{0-inf}, $T_{max,ss}$, V_{ss}, $t_{1/2}$, CL, $C_{av,ss}$, DF, Range, $\%AUC_{ext}$, $R_{1ac}=C_{max,ss}/C_{max}$, $R_{2ac}= AUC_{0-\tau}/AUC_{0-t}$ after multiple doses. The mean and standard deviation of each parameter are also calculated.<br>(2) Stage II dose expansion study<br>Sparse blood collection is performed. Other JMT101 concentration data will be pooled for population PK analysis. See separate reports for details and analysis results.<br><br>■ Immunogenicity analysis:<br>The number of cases and incidence of positive ADA and NABs are listed by dose group and total.<br><br>■ Biomarker analysis:<br>Pharmacodynamic biomarkers: The cut-off value is determined based on the level of free DNA in plasma and the low and high expression groups are identified based on this value. The Kaplan-Meier method is used to estimate the median PFS, median OS and their 95% CIs for all subjects and each dose group, and survival curves will be plotted.
Trial duration:	The study consists of two stages, and the study process in each stage mainly includes screening, treatment, safety follow-up and survival follow-up. Each subject will receive investigational drug for approximately 6 months. Subjects will receive a safety follow-up visit 30 days (±3 days) after the last dose specified in the study protocol. The survival follow-up will be conducted thereafter.<br>The specific number of subjects to be included in each study stage could not be clearly predicted in this study. In addition, the duration of the study may also vary among subjects, depending on the need for

	subsequent treatment and progression-free survival time. Therefore, the duration of the entire study is only a preliminary estimate. For subjects who complete the entire study from the first dose of investigational drug to the survival follow-up, the duration of the entire study will be approximately 3.5 years.
Duration of visit:	Provisionally 1.5 years

1.2 Study Process Schema

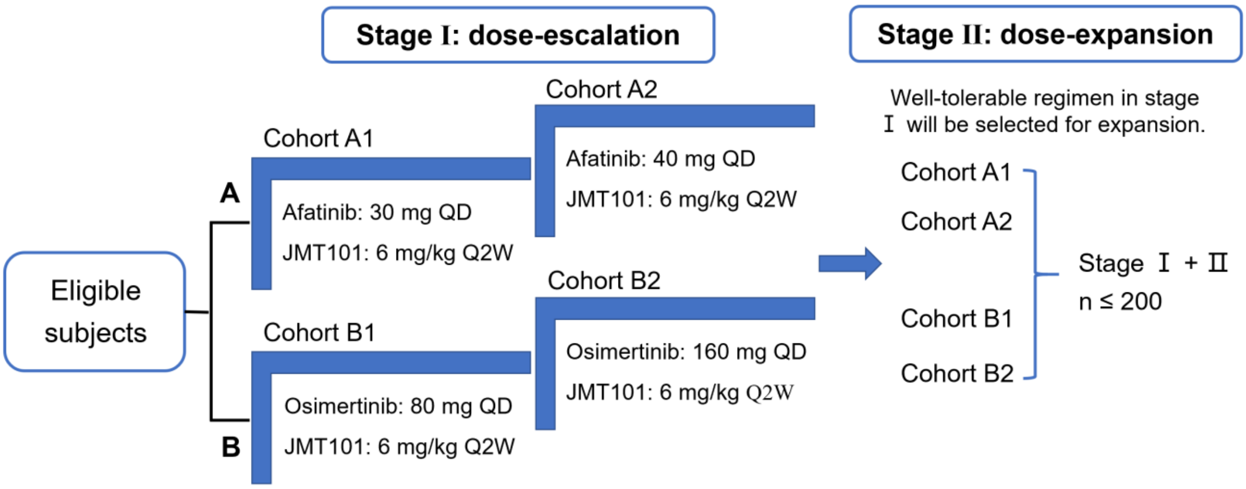

Figure 1-1 Schema of the Overall Study Process

1.3 Schedule of Activities (SOA)

Schedule 1-1 Study Process Schema

Assessment	Screening[1] / baseline period[2]		Cycle 1 C1		Cycle 2 C2		Cycle 3 C3		Cycle 4 C4		Subsequent cycle C5 and beyond	End-of-treatment visit (EOT) [3]	Safety follow-up[4]	Survival follow-up[5]
Day	-28~-1													
	-28~-8	-7~-1	D1	D15	D1	D15	D1	D15	D1	D15	Every 14 days	Within 7 days after the last dose	Within 30 ± 3 days after the last dose	Once every 8 weeks
Time window (day)	-	-	±2	±2	±2	±2	±2	±2	±2	±2	±2	±3		±7
Signing informed consent form	X													
Inclusion/exclusion criteria	X													
Demographic data	X													
Medical history and medication history	X													
EGFR gene	X													
Height/weight[6]	X		X					X		X	X	X		
Vital sign[7]		X	X					X		X	X	X		
Physical examination[8]		X						X		X	X	X		
ECOG score		X	X					X		X	X	X		
Routine blood [9]		X						X		X	X	X		
Routine urine [9]		X						X		X	X	X		

Assessment	Screening[1] / baseline period[2]		Cycle 1 C1		Cycle 2 C2		Cycle 3 C3		Cycle 4 C4		Subsequent cycle C5 and beyond	End-of-treatment visit (EOT) [3]	Safety follow-up[4]	Survival follow-up[5]
Day	-28~-1													
	-28~-8	-7~-1	D1	D15	D1	D15	D1	D15	D1	D15	Every 14 days	Within 7 days after the last dose	Within 30 ± 3 days after the last dose	Once every 8 weeks
Time window (day)	-	-	±2	±2	±2	±2	±2	±2	±2	±2	±2	±3		±7
Blood biochemistry[9]		X						X		X	X	X		
Coagulation[9]		X						X		X	X	X		
Blood pregnancy test (if applicable)[10]		X										X		
Virological screening[11]		X												
12-lead ECG[12]		X						X		X	X	X		
Echocardiography[13]	X		Every 3 cycles (± 7 days)											
Tumor assessment[14]	X		X		Every 2 cycles (± 7 days)									
Tumor tissue sample collection (optional)[15]	X													
PK sample collection[16]			X										X	
Immunogenicity sample collection[17]			X										X	

Assessment	Screening[1] / baseline period[2]		Cycle 1 C1		Cycle 2 C2		Cycle 3 C3		Cycle 4 C4		Subsequent cycle C5 and beyond	End-of-treatment visit (EOT) [3]	Safety follow-up[4]	Survival follow-up[5]
Day	-28~-1<br>-28~-8	-7~-1	D1	D15	D1	D15	D1	D15	D1	D15	Every 14 days	Within 7 days after the last dose	Within 30 ± 3 days after the last dose	Once every 8 weeks
Time window (day)	-	-	±2	±2	±2	±2	±2	±2	±2	±2	±2	±3		±7
Biomarker blood sample collection[18]	X		X										X	
JMT101 administration			Once every two weeks											
Afatinib or Osimertinib Administration			Continuous administration											
Concomitant medication and treatment[19]	X													
Adverse event[20]	X													
Survival														X

Notes:

1. **Screening period**: Patients are required to sign an ICF prior to any study-related procedures. If an assessment performed for other purposes prior to informed consent also applies to the screening evaluation (within the protocol time window and meeting examination content requirements), the examination may not be repeated with the consent of the patient / legal representative.
2. **Baseline examination during the screening period**: CT/MRI/bone scan results and echocardiography within 28 days prior to first dose, ECOG score and laboratory results (routine blood, routine urine, blood biochemistry, coagulation, virological screening, blood pregnancy test) within 7 days prior to first dose, vital signs and physical examination within 1 day prior to first dose are acceptable.
3. **End-of-treatment visit**: The end date of treatment is the date on which the investigator confirms that the subject needs to discontinue treatment for any reason. The end-of-treatment visit needs to be completed within 7 days after the last dose. If a decision is made to discontinue treatment at one visit (e.g., due to disease progression), that visit can also be considered as an end-of-treatment visit. If a patient fails to return to the study site for an EOT visit, this patient should be contacted to collect any adverse

events that occurred.

4. **Safety follow-up**: All subjects, including those who withdraw early from the study, should complete a safety follow-up visit (i.e., assessment of AE and/or SAE, and concomitant medication) within 30 days (±3 days) after the last dose or prior to new anti-tumor therapy, whichever occurs first. Patients whose treatment is interrupted or permanently discontinued due to adverse events (including abnormal laboratory results) must be followed until the event is recovered or stabilized, whichever occurs earlier.
5. **Survival follow-up**: Follow-up of progression-free survival: For patients who discontinue treatment before disease progression, tumor assessment should be performed every 8 weeks ± 7 days after the last dose until disease progression, loss to follow-up, death, or change of treatment regimen (whichever occurs first). Follow-up of overall survival: Subjects with confirmed disease progression or initiation of new anti-tumor therapy will enter a survival follow-up period. After the end of trial treatment, survival status and subsequent anti-tumor therapy condition are collected through clinical follow-up or telephone follow-up every 8 weeks ± 7 days until death, loss to follow-up, or end of study (whichever occurs first).
6. **Height/weight**: Height is measured during the screening period only, and weight is measured during the screening period, at each visit during the treatment period (before JMT101 administration), and at the end-of-treatment visit.
7. **Vital sign**: during the screening period, before C1D1 administration (-30 min) and 2 h (±30 min), 4 h (±30 min) and 8 h (±30 min) after the end of administration; on C1D15, C2D1, C2D15, C3D1, C3D15, C4D1, and C4D15 (all ±2 days); every 14 (±2) days during the subsequent cycles and at the end-of-treatment visit.
8. **Physical examination**: during the screening period, on C1D15, C2D1, C2D15, C3D1, C3D15, C4D1, and C4D15 (all ±2 days), every 14 (±2) days during the subsequent cycles, and at the end-of-treatment visit.
9. **Laboratory tests**: including blood routine, urine routine, blood biochemistry, coagulation function. Time points for laboratory tests: during the screening period, on C1D15, C2D1, C2D15, C3D1, C3D15, C4D1, and C4D15 (all ±2 days), every 14 (±2) days during the subsequent cycles, and at end-of-treatment visit.
10. **Blood pregnancy test**: Women of childbearing age undergo a blood pregnancy test during the screening period and at the end-of-treatment visit.
11. **Virological screening**: only during the screening period. It includes: 5 markers of hepatitis B, hepatitis C virus antibody (HCV-Ab), human immunodeficiency virus antibody (HIV-Ab), and treponema pallidum antibody (RPR or TRUST). Subjects with positive HBsAg and HCV-Ab should be tested for HBV-DNA and HCV-RNA virus quantitative detection.
12. **12-Lead ECG**: during the screening period, on C1D15, C2D1, C2D15, C3D1, C3D15, C4D1, and C4D15 (all ±2 days), every 14 (±2) days during the subsequent cycles, and at the end-of-treatment visit. If a prolonged QTcF interval occurs in a subject, the investigator may increase the frequency of examination depending on the subject’s condition. Subjects should rest for at least 10 minutes before each examination.
13. **Echocardiography**: during the screening period, at the end of every 3 treatment cycles, and at the end-of-treatment visit.
14. **Tumor assessment**: during the screening period, at the end of Cycle 1 (±7 days) and at the end of every 2 cycles thereafter (Cycle 3,

Cycle 5 ......, ±7 days), at the end-of-treatment visit. If the dose is delayed, the corresponding imaging examination time points should be also delayed, but not more than 10 weeks after the last tumor assessment. Subsequent tumor assessment methods should be consistent with those in the screening period as much as possible. If the patient's tumor-associated symptoms worsen during the treatment, tumor assessment may be performed earlier as determined by the investigator. For patients who discontinue treatment before disease progression, tumor assessment should be performed every 8 weeks ± 7 days after the last dose until disease progression, loss to follow-up, death, or change of treatment regimen (whichever occurs first). If the subject have a tumor assessment within 4 weeks prior to the end-of-treatment visit, no additional tumor assessment is required at the end-of-treatment visit.

15. **Tumor tissue sample collection**: This is optional. For those who cannot provide the detection report of EGFR exon 20 insertion mutation, tumor tissue samples from subjects may be collected during the screening period for diagnostic biomarker detection if the subject is willing to provide it.
16. **PK sample collection**: See Schedule 1-2 and Schedule 1-3 for details.
17. **ADA sample collection**: See Schedule 1-4 for details.
18. **Biomarker blood sample collection**: diagnostic biomarker blood sample: For those who cannot provide the detection report of EGFR exon 20 insertion mutation during the screening period, 10 mL of peripheral blood specimen will be collected for EGFR exon 20 insertion mutation detection if the subject is unable to provide sufficient tumor tissue samples. For those who can provide the detection report of EGFR exon 20 insertion mutation, diagnostic biomarker sample collection is not required. Pharmacodynamic biomarker blood sample: 10 mL of blood is collected before the first dose (within 30 min), at the time of first efficacy assessment and disease progression, respectively.
19. **Concomitant medication and treatment**: All concomitant medications and treatments from the date of signing the informed consent until the safety follow-up period (or until the subject starts a new anti-tumor therapy) are documented.
20. **Adverse event**: Information is collected on all adverse events from the date of signing the informed consent until the safety follow-up period (or until the subject starts a new anti-tumor therapy, whichever occurs first).

*****If the dose is delayed due to toxicity during the treatment, the subsequent safety assessment and tumor assessment time points will be delayed accordingly. The investigator may increase the frequency of laboratory tests or add unplanned additional tests depending on clinical indications. In case of holidays or major social public events, the corresponding dosing and safety visit schedule may be postponed as necessary.

Schedule 1-2 PK blood collection time point (Stage I dose escalation study)

Visit days	Cycle 1 C1							Cycle 2 C2							Cycle 3 C3	Within 30± 3 days after the last dose
	1	2	3	5	8	11	15	29	30	31	33	36	39	43	57	
Pre-dose (-30 min)	X						X	X						X	X	
0 min post-dose (+2 min)	X						X	X						X	X	
4 h post-dose (±15 min)	X							X								
8 h post-dose (±30 min)	X							X								
24 h post-dose (±1 h)		X							X							
48 h post-dose (±2 h)			X							X						
96 h post-dose (±4 h)				X							X					
168 h post-dose (±7 h)					X							X				
240 h post-dose (±12 h)						X							X			
30 days after the last dose (±3d)																X

Notes:

1. PK sampling time should be as close as possible to the specified time. To this end, some other assessments scheduled at the same time can be advanced or delayed to allow for completion of the necessary testing, and time should be set aside for PK blood sample collection at the specified time point.
2. If a patient develops intolerance that will lead to drug discontinuation, blood samples can be collected on the same day for PK analysis.
3. 2.5 mL of blood is collected each time.

Schedule 1-3 PK blood collection time point (Stage II dose expansion study)

Visit days	Cycle 1 C1				Cycle 2 C2				Cycle 3 C3	Within 30± 3 days after the last dose
	1	2	8	15	29	30	36	43	57	
Pre-dose (-30 min)	X			X	X			X	X	
0min post-dose (+2 min)	X			X	X			X	X	
8 h post-dose (±30 min)	X				X					
24 h post-dose (±1 h)		X				X				
168 h post-dose (±7 h)			X				X			
30 days after the last dose (±3d)										X

Notes:

1. PK sampling time should be as close as possible to the specified time. To this end, some other assessments scheduled at the same time can be advanced or delayed to allow for completion of the necessary testing, and time should be set aside for PK blood sample collection at the specified time point.
2. If a patient develops intolerance that will lead to drug discontinuation, blood samples can be collected on the same day for PK analysis.
3. 2.5 mL of blood is collected each time.

Schedule 1-4 ADA blood collection time point

Visit days	1	15 (±1d)	29 (±1d)	57 (±3d)	30d after the last dose (±3d)
Pre-dose (i.e. before the first dose, -30 min)	X				
15d post-dose (i.e. before the 2nd dose, -30 min)		X			
29d post-dose (i.e. before the 3rd dose, -30 min)			X		
57d post-dose (i.e. before the 5th dose, -30 min)				X	
30d after the last dose					X

Notes:

1. 3.5 mL of blood samples are collected for immunogenicity test within 30 minutes before administration.
2. Samples are first tested for anti-drug antibodies. Samples that are tested positive for anti-drug antibodies need to be further tested for neutralizing antibodies.

2 Introduction

2.1 Study Rationale

2.1.1 Epidermal growth factor receptor

The epidermal growth factor receptor (EGFR), a member of ErB family, is a transmembrane glycoprotein with ligand-induced tyrosine protein kinase activity. When bound to ligands such as EGF and TGFα, EGFR activates downstream signaling pathways such as Ras/RAF/MAPK and PI3K/Akt by forming homodimerization or activating post-receptor tyrosine kinase activity with other heterodimers to regulate normal cell proliferation, survival, differentiation, migration, adhesion, repair and other cascade cell response effects[1]-[4].

EGFR is a transmembrane receptor that is structurally divided into three parts: extracellular region (extracellular ligand binding domain), transmembrane domain, and intracellular domain (intracellular tyrosine kinase domain). When the extracellular domain of EGFR binds to the corresponding ligand, the intracellular tyrosine kinase domain is activated, leading to autophosphorylation, which provides docking sites for a variety of downstream molecules and initiates downstream signaling pathways[5]. Several studies have confirmed that blocking the EGFR signaling pathway can inhibit EGFR phosphorylation and activation of downstream signals to achieve tumor cell growth inhibition and anti-tumor effects[6]-[8]. Epidermal growth factor receptor monoclonal antibodies act on the extracellular ligand binding domain and epidermal growth factor receptor tyrosine kinase inhibitors (EGFR-TKIs) act on the intracellular tyrosine kinase domain, both of which inhibit tumor growth, metastasis and invasion by blocking the EGFR signaling pathway.

EGFR is highly or abnormally expressed in many solid tumors, such as colorectal carcinoma, esophageal squamous carcinoma, head and neck squamous carcinoma, non-small cell lung cancer and other malignancies[9]-[10]. EGFR overexpression causes enhanced downstream signaling, is involved in tumor cell proliferation and promotes angiogenesis, tumor invasion and metastasis[11]-[14]. Many tumors have mutant EGFR. The EGFR gene is located on the short arm of chromosome 7 (7p12-14), which is approximately 118 kb long and consists of 28 exons[5]. The genes encoding the EGFR kinase domain are located in exons 18 to 24. EGFR mutations are one of the important driver genes of NSCLC, and EGFR mutations in NSCLC patients mainly occurs in exons 18~21. Mutations in the gene encoding the EGFR kinase domain may result in increased receptor kinase activity. Positive EGFR-sensitive mutation is a prerequisite for effective treatment of NSCLC with EGFR-TKIs-targeted drugs.

2.1.2 Epidemiological characteristics of NSCLC with EGFR exon 20 insertion mutations

Lung cancer is a malignant tumor with the highest incidence and mortality rate in China and worldwide, which seriously threatens patients' health. Most patients are already at a late stage when diagnosed, and have a low 5-year survival rate and a high disease burden. About 85-90% of lung cancers are caused by active or passive smoking. Lung cancer can be divided into non-small cell lung cancer (NSCLC) and small cell lung cancer according to histological characteristics, with NSCLC accounting for about 85%[15] of all lung cancers. A variety of molecular mechanisms, including mutations and expression abnormalities, have been shown to be associated with NSCLC pathogenesis, with EGFR being one of the major driver genes. With the identification of a series of oncogenic driver genes of lung cancer, targeted drugs have greatly improved the prognosis and prolonged the survival of NSCLC patients carrying

the corresponding driver genes. The type of lung cancer has also been further subdivided into molecular subtypes based on driver genes from the simple pathological histological classification in the past.

The positive rate of EGFR mutations in NSCLC patients from Asian populations and China is 40-50%[16]-[18]. The proportion of EGFR mutations differs between Asians and Westerners. There are four main types of EGFR mutations: exon 19 deletion mutation, exon 21 point mutation, exon 18 point mutation and exon 20 insertion mutation[19]. The most common EGFR mutations are exon 19 deletion mutation (19DEL) and exon 21 L858R point mutation, both of which are EGFR TKI-sensitive mutations. Mutations of G719X in exon 18, S768I in exon 20, and L861Q in exon 21 are also sensitive mutations. Rare EGFR mutations account for about 15-20% of all EGFR mutations, and more than 50% of these rare mutations are located in EGFR exon 20, with EGFR exon 20ins being the most common[20].

In lung cancer patients with EGFR mutations, the EGFR exon 20 insertion mutation, referred to as EGFR 20ins, is the most common type of mutation among rare EGFR mutations, accounting for about 30% of rare EGFR mutations. Studies have reported that EGFR 20ins accounts for 4.8-12% of all EGFR mutations in Western population, and there are more than 30 known types of EGFR 20ins mutations[21]-[22]. EGFR 20 insertion mutations account for 3-4% of EGFR mutations in Chinese population. The genetic characteristics of EGFR 20ins mutations in Chinese NSCLC patients is comparable to that reported in Caucasian patients, and both account for approximately 2-3% of total NSCLC in terms of prevalence, including most unusual mutations and the most prevalent co-mutations[22],[23]-[25].

2.1.3 Unmet clinical needs of NSCLC with EGFR exon 20 insertion mutations

The relationship between the efficacy of precision-targeted therapy and molecular subtyping in advanced EGFR-sensitive mutation-positive NSCLC has been fully confirmed in clinical practice. EGFR-TKIs now have become the first-line standard of care treatment for EGFR-sensitive mutant non-small cell lung cancer. Among them, Afatinib, the second-generation EGFR TKIs, has been approved in China for first-line treatment of EGFR-sensitive mutant NSCLC. Osimertinib is a third-generation EGFR TKIs and the recommended drug for first-line treatment of EGFR-sensitive mutations in many international guidelines. The drug has been approved in China for first-line treatment of EGFR-sensitive mutant NSCLC and later-line treatment of T790M-positive NSCLC.

However, the insensitivity of EGFR 20ins patients to first- and second-generation EGFR-TKI therapy has been confirmed by several clinical studies. In a clinical study of 11 lung cancer patients with EGFR 20ins treated with Erlotinib, only 3 achieved partial response (PR), and the time to tumor progression (TTP) was only 3 months[26]. A post-marketing combination analysis of LUX-Lung 2, LUX-Lung 3 and LUX-Lung 6 also showed that patients with EGFR 20ins had the least benefit from Afatinib treatment compared to patients with other mutations, with ORR of 8.7%, median PFS of 2.7 months and median OS of 9.2 months in 23 patients with exon 20ins receiving first-line treatment of Afatinib, which were the shortest among all patients with EGFR mutations, suggesting that Afatinib alone had limited efficacy against exon 20ins mutations[19]. Studies[19],[22],[26]-错误!未找到引用源。 showed that when treated with first- and second-generation EGFR-TKIs, patients carrying exon 20 insertion mutations had an overall effective rate of 0% to 11%, a median progression-free survival of 2 to 3 months, and an overall survival that was similar to that of patients with wild-type EGFR. The National Comprehensive Cancer Network (NCCN) guidelines for NSCLC also pointed out that: most EGFR 20 exon insertion mutations predicted clinical resistance to TKI; a rare EGFR 20 exon insertion variant

(p.A763_Y764insFQEA) was the exception, which was associated with onset of effect of EGFR TKI therapy; therefore, the understanding of EGFR 20 exon insertion must include clear sequence changes.

The 2018 European Society of Medical Oncology Annual Meeting (ESMO) released preliminary data from a US phase II clinical study of Osimertinib in advanced NSCLC patients with positive EGFR exon 20 insertion mutations (NCT 03191149) with median of prior systemic therapy lines of 1 (range 0 to 3): of all 17 patients treated with Osimertinib, only one patient showed partial response, with an ORR of 6%, a median PFS of 3.7 months and a DCR of 35% at five months (6/17). It was concluded that Osimertinib had limited anti-tumor activity in NSCLC patients with EGFR exon 20 mutations[28]. In 2019, ESMO published preliminary data from another Korean phase II clinical study evaluating the efficacy of Osimertinib in advanced NSCLC patients with EGFR exon 20 insertion mutations after failure of standard chemotherapy (NCT03414814): From January 2018 to February 2019, 15 patients received Osimertinib as second-line treatment (20%, n=3) and ≥ third-line treatment (n=12) in the first stage; the ORR was 0%, and most diseases were stable (stable disease, accounting for 46.7%, n = 7); the median PFS was 3.5 months (95% CI 1.6-NR), and the median OS was not reached (1-year OS rate of 56.3%); the DCR at 6 months was 31.1%; and the most common adverse events (all grades) were nausea (20%, n=3), vomiting (20%, n=3), anemia (13.3%, n=2) and fever (13.3%, n=2). It was concluded that Osimertinib was well tolerated but had limited efficacy in NSCLC patients with EGFR exon 20 insertion mutations after failure of standard chemotherapy[29].

In summary, it was shown that first-, second- and third-generation EGFR TKIs were less effective in NSCLC patients with EGFR exon 20 insertion mutations. The current first-line standard of care treatment for this patient population was chemotherapy, and the ORR to systemic chemotherapy was only 20-40%[30]-[32], showing poor efficacy. Currently, there is no targeted drug approved for this target population and no recommended regimen in domestic and international clinical guidelines, so there is an unmet clinical need for treatment of NSCLC with EGFR 20ins mutations. Although the proportion of patients with EGFR 20ins mutations is small, it is still a large group that cannot be ignored considering the number of lung cancer patients in China and worldwide. If effective treatment regimens can be found, it will undoubtedly contribute greatly to the overall survival of lung cancer patients.

The investigational drug “Recombinant Humanized Anti-epidermal Growth Factor Receptor (EGFR) Monoclonal Antibody Injection (JMT101)” in this clinical study is a targeted drug for EGFR independently developed by Shanghai JMT-Bio Inc. It is a fully humanized monoclonal antibody IgG1 subtype. Pre-clinical studies have confirmed that JMT101 is similar to its marketed similar drugs in terms of protein structure, physicochemical properties and freeze-thaw stability, and has obvious anti-tumor effects in vitro and in vivo, with good safety profiles. This study will use JMT101 in combination with Afatinib or Osimertinib to initially explore the treatment of stage IIIB or IV non-small cell lung cancer with EGFR exon 20 insertion mutations, so as to provide a new treatment strategy for lung cancer patients with EGFR exon 20 insertion mutations.

2.2 Background

2.2.1 Investigational drug

Generic name: Recombinant Humanized Anti-epidermal Growth Factor Receptor (EGFR) Monoclonal Antibody Injection

Abbreviation: JMT101

English name: Recombinant Humanized Anti-EGFR monoclonal Antibody Injection

Chinese pinyin: Chongzu Renyuanhua Kang Biaopishengzhangyinzishouti Dankelongkangti Zhusheye

Molecular structure: 148,132 Dalton (Da)

Active ingredient: Recombinant Humanized Anti-epidermal Growth Factor Receptor (EGFR) Monoclonal Antibody

2.2.2 Overview of Pre-clinical Study

Pharmacodynamic studies

In vitro studies showed that the affinity of JMT101 with human EGFR was about 7 times that of cetuximab and about 2 times that of panitumumab; the affinity of JMT101 with cynomolgus monkey EGFR was about 6 times that of cetuximab and about 3 times that of panitumumab. JMT101 had similar antibody-dependent cell-mediated cytotoxicity (ADCC) and complement-dependent cytotoxicity (CDC) effects to cetuximab. The results of in vitro tests in eight cell lines showed that JMT101 and cetuximab had comparable inhibitory effects on tumor cells (including DiFi, A431, NCI-H292, HCC-827, etc.) at equivalent doses, similar inhibitory effects on EGFR-mediated signaling pathways, and generally consistent inductive effect on cell cycle arrest at G1 phase. In the cell proliferation assay of Ba/F3 EGFR-Exon20 V769_D770insASV cell line, JMT101 showed a significant synergistic effect in combination with Afatinib or Osimertinib.

In vivo pharmacodynamic studies showed that JMT101 and cetuximab had comparable tumor suppressive effects on DiFi, A431, FaDu, and Eca109 tumor-bearing mice at equivalent doses (1.5 mg/kg and 5 mg/kg). In the Ba/F3 EGFR Exon20 V769_D770insASV subcutaneously xenograft tumor model, JMT101 had an approximate synergistic or additive effect in combination with Afatinib or Osimertinib; the tumor suppressive effects of the combination were both significantly stronger than those of Afatinib or Osimertinib alone.

Pharmacokinetic studies

Pharmacokinetic studies showed that after single intravenous infusion of JMT101 and cetuximab at the same dose (7.5 mg/kg) in cynomolgus monkeys, the plasma concentration-time changes and pharmacokinetic behaviors were similar, with JMT101 plasma exposure slightly higher than that of cetuximab and with a relative bioavailability of 116.6%. In cynomolgus monkeys given multiple consecutive intravenous doses of JMT101 (7.5 mg/kg once a week for 4 consecutive doses), the last drug exposure was higher than that of a single dose, with an accumulation index of approximately 1.0.

Toxicity studies

Acute toxicity studies: In toxicological studies, JMT101 was given by single IV infusion at doses of 30, 100, and 300 mg/kg, and no animal death was observed; MTD >300 mg/kg.

4-week repeat-dose toxicity study in cynomolgus monkeys with a 4-week recovery period

JMT101 was administered once a week for 4 consecutive weeks at doses of 15, 25, and 60 mg/kg with a 4-week recovery period, and two females in the high-dose group died early. The cause of these deaths may be related to dehydration due to diarrhea and rash associated with EGFR targets. Other clinical manifestations were mainly rash, diarrhea, ulceration and other symptoms associated with EGFR targets, and no toxicity reactions not related to EGFR targets were seen. The animals were in good condition during the recovery period, and all drug-related toxic manifestations were significantly reduced. The highest non-severely toxic dose (HNSTD) was 25 mg/kg.

13-week repeat-dose toxicity study in cynomolgus monkeys with an 8-week

recovery period

JMT101 (7.5, 20 or 45 mg/kg, administered once a week by intravenous infusion for 13 repeated doses) was given to cynomolgus monkeys for 13 weeks with an 8-week recovery period. The results showed that biological toxicity reactions related to the target of JMT101 were seen in animals in all dose groups of JMT101. Toxicity reactions included secondary animal death caused by gastrointestinal reactions and skin damage, and symptoms such as skin damage and gastrointestinal reactions. The histopathological examination showed thickening of skin epidermal spine layer, dermatitis, crusting, ulceration and epidermal/dermal/subcutaneous inflammatory cell infiltration, and extensive thymus or cortical cell reduction, all of which were fully recovered at the end of the recovery period. The main toxic target organs were the skin and thymus.

General pharmacological studies: Safety pharmacological study. The safety pharmacological study of JMT101 in cynomolgus monkeys was carried out concomitantly with the repeated-dose toxicity study at doses of 15, 25 and 60 mg/kg, respectively. In the female animals in the high-dose group, symptoms such as depression, abnormal spontaneous activity and decreased heart rate were found, which may be caused by secondary reactions. At doses of 15-60 mg/kg, JMT101 showed no significant effects on the central nervous system, respiratory system and cardiovascular system of cynomolgus monkeys. JMT101 was given to mice by single intravenous administration at 10, 50 and 250 mg/kg, showing no significant effect on the function of the central nervous system of mice. JMT101 had no synergistic effect with pentobarbital sodium, and 250 mg/kg was a safe dose.

Tissue cross-reactivity: The specific binding of JMT101 to normal human frozen tissue, normal cynomolgus monkey frozen tissue and cynomolgus monkey frozen skin tissue was essentially identical to that of cetuximab.

Local irritation test and hemolysis test: the results were negative.

2.2.3 Clinical Studies

The Phase I clinical study of JMT101, titled “Phase I Clinical Study to Evaluate the Safety, Tolerability, and Pharmacokinetics of JMT101 for the Treatment of Advanced Solid Tumors” (protocol number: JMT101-ECL), was a first-in-human clinical study. The objective of the study was to evaluate the safety, tolerability, pharmacokinetics and preliminary pharmacodynamics of JMT101 in advanced solid tumors. The study was divided into two stages: dose escalation and expansion. The dose escalation stage adopted accelerated titration and standard “3 + 3” method. In JMT101 monotherapy regimen, the initial dose of JMT101 was 0.5 mg/kg (QW), and was sequentially increased to 2.0, 4.0, 6.0, 8.0, and 10.0 mg/kg (Q2W) in each dose group; in the combination chemotherapy regimen, JMT101 dose was sequentially administrated at doses of 6.0 and 8.0 mg/kg (Q2W). 139-259 subjects are planned to be enrolled. As of August 12, 2020, 50 subjects with advanced colorectal cancer had been enrolled in this study, including 29 in the dose-escalation stage and 21 in the expansion stage. Preliminary results showed no cases of dose-limiting toxicity (DLT); investigational drug-related adverse event with a high incidence was rash, which had severity of Grade 1 to 3 (according to CTCAE 4.03); there were no Grade 4 rash at that time, and no SAEs definitely related to the investigational drug. After repeated dose of JMT101, the exposure increased with dose, and the half-life tended to be prolonged with increasing dose ($T_{1/2}$ of 48.92-133.81 h) in the dose range of 2-10 mg/kg. Of the 38 evaluable tumor cases, the best response was partial response (PR) in 11 cases (2 cases with monotherapy and 9 cases with combination chemotherapy), stable disease (SD) for more than 12 weeks in 16

cases (7 cases with monotherapy and 9 cases with combination chemotherapy), and SD for more than 60 weeks in 1 case (monotherapy). The above monotherapy was second-line or above treatment, and the combination chemotherapy was mainly first- or second-line treatment.

2.2.4 Overview of Similar Drug Studies

Marketed Similar Drugs

Clinical studies showed targeted EGFR monoclonal antibody drugs used alone or in combination with radiotherapy/chemotherapy both had a higher disease control rate, and significantly improved progression-free survival (PFS) and/or overall survival (OS) in tumor patients[34]-[36]. The common adverse reactions were mild to moderate in severity and controllable, mainly manifested as rash, infusion reaction and hypomagnesemia. Cetuximab (trade name: ERBITUX®) was the first anti-EGFR monoclonal antibody (human-mouse chimeric IgG1) to be approved for marketing. Other approved drugs included the humanized IgG1 monoclonal antibody Nimotuzumab (trade name: BIOMAb-EGFR®) and Necitumumab (trade name: PORTRAZZA), and the humanized IgG2 monoclonal antibody Panitumumab (trade name: VECTIBIX®). Their indications included metastatic colorectal cancer, advanced head and neck squamous cell carcinoma and advanced squamous non-small cell lung cancer[37][40]. In advanced colorectal cancer, the FDA approved cetuximab alone or in combination with chemotherapy regimens for the treatment of KRAS wild-type, EGFR-expressing metastatic colorectal cancer[37]. Panitumumab was approved by FDA for the treatment of RAS wild-type metastatic colorectal cancer as first-line therapy in combination with FOLFOX, and was also approved as monotherapy in patients with disease progression under chemotherapy containing fluorouracil, oxaliplatin and irinotecan[38]. NCCN guidelines and CSCO guidelines recommend FOLFIRI or FOLFOX in combination with anti-EGFR monoclonal antibodies as the first-line treatment in patients with RAS/BRAF wild-type colorectal cancer[41]. Other indications: cetuximab in combination with radiotherapy for locally or regionally advanced squamous cell carcinoma of the head and neck; combination of 5-fluorouracil and platinum-based chemotherapy for locally recurrent or metastatic squamous cell carcinoma of the head and neck; recurrent or metastatic squamous cell carcinoma of the head and neck that has progressed again after platinum-based chemotherapy[37]. Necitumumab in combination with gemcitabine/cisplatin was approved for the first-line treatment of locally progressive or metastatic squamous non-small cell lung cancer[39].

In China, cetuximab and nimotuzumab were approved by the CFDA for the following clinical use: cetuximab for the treatment of RAS gene wild-type metastatic colorectal cancer expressing epidermal growth factor receptor (EGFR); cetuximab in combination with irinotecan for patients who experienced failure of irinotecan-containing therapy; nimotuzumab in combination with radiotherapy for treatment of patients with stage III/IV EGFR-positive nasopharyngeal carcinoma[40],[42].

Studies of Similar Drug Use in Target Populations

Currently, no anti-EGFR monoclonal antibody has been approved for the treatment of NSCLC with EGFR exon 20 insertion mutations in China or abroad. In vitro tests, animal models, and small sample clinical studies initially showed good therapeutic effects of EGFR monoclonal antibodies in combination with Afatinib or Osimertinib in treating EGFR 20ins. A study using in vitro cytologic test and tumor-bearing mice in vivo test found that cetuximab alone was less effective in tumor cells with EGFR 20ins mutations, while a combination regimen of cetuximab with Afatinib or Osimertinib inhibited the growth of cells carrying

partial mutations or transplanted tumors. Cetuximab enhanced Afatinib- and Osimertinib-mediated pro-apoptotic activity of EGFR 20ins and also enhanced the inhibition of pEGFR and downstream signaling pathways[42]. Using a PDX model, another study found that tumors of PDX mice carrying EGFR 20 ins (N771_P772>SVDNP) + EGFR-amplified tumor cells responded to treatment with Osimertinib in combination with cetuximab, and tumor growth was inhibited[43]. A Dutch study attempting to use Afatinib in combination with cetuximab regimen in 4 clinical patients found that 3 patients had treatment response. Clinically, it was Preliminarily clinical results showed high efficiency of Afatinib combined with cetuximab, but relatively high toxicity, with 2 patients having dose reductions due to toxicity[44]. Chinese scholars reported for the first time that Osimertinib in combination with cetuximab was effective in NSCLC patients with EGFR 20 ins and suggested that high-dose Osimertinib may be a treatment regimen for well-tolerated patients[45]. Although these results still need to be validated in large clinical studies, they certainly offer a promising new direction for the treatment of NSCLC patients with EGFR 20 ins. A single-arm, open-lable, multi-center phase II clinical study of Afatinib in combination with cetuximab in NSCLC with EGFR exon 20 insertion mutations (NCT03727724) is currently underway overseas with an expected sample size of 37 cases, and no relevant data have been released yet.

2.2.5 Known Potential Risks

No human study data have been reported for JMT101 and therefore there are no clinically known significant risks. The main symptoms observed in the pre-clinical repeat-dose toxicity study of cynomolgus monkeys were skin rash, diarrhea, ulceration and other EGFR target related symptoms, and the main toxic target organs were skin and thymus.

For risk control, subjects with high expected safety risks will be excluded. Subjects are required to have recovered to ≤ Grade 1 from toxicity reactions of previous anti-tumor therapies at enrollment, except for alopecia etc., which, in the judgment of the investigator, is not clinically significant, and to have adequate organ function.

After subjects are enrolled after screening, they will be closely monitored for safety parameters, including vital signs, physical examination, routine blood, blood biochemistry (liver function, kidney function, electrolytes, cardiac enzymes, etc.), 12-lead ECG, and echocardiography during the treatment period and 30 days after the last dose of investigational drug. At the same time, the occurrence of adverse events from the first dose to 30 days after the last dose will be closely monitored and recorded. For serious adverse events (SAE) and suspected unexpected serious adverse reactions (SUSAR), they will be reported in accordance with the relevant laws and regulations.

Regarding the expected toxicity of the investigational drug and toxicity management measures, the sponsor has developed a detailed risk management plan. For pharmacovigilance, the sponsor has established a professional team to collate, evaluate and analyze adverse events, and will notify the investigator in a timely manner when significant or unexpected safety risks occur, and hold a meeting with the investigator to discuss them if necessary. The sponsor will notify the investigator immediately if any additional safety or toxicological information is found during the study.

2.2.6 Known Potential Benefits

This clinical study enrolls stage IIIB or IV non-small cell lung cancer (NSCLC) subjects with EGFR exon 20 insertion mutations. Pre-clinical data showed that the affinity of JMT101 with human EGFR was about 7 times that of cetuximab, and its structure and mechanism of action were similar to those of cetuximab, showing significant in vivo and in vitro anti-tumor effects. Cetuximab is a chimeric monoclonal antibody, and pre-clinical studies have showed

that cetuximab in combination with Afatinib or Osimertinib have significant anti-tumor activity against EGFR exon 20 insertion mutation[43]-[44]. Small sample clinical studies in china and abroad have also reported[45]-[46] that cetuximab in combination with Afatinib or Osimertinib is effective and tolerable in NSCLC patients with EGFR exon 20 insertion mutations. Based on these findings, this study will develop a combination regimen for NSCLC with EGFR exon 20 insertion mutations. Therefore, participation in this study may bring clinical benefits to the subjects.

2.2.7 Assessment of Potential Risks and Benefits

Based on the results of the non-clinical safety study of this product, corresponding preventive and treatment measures have been proposed for the possible clinical study risks of this product, and a clinical risk control plan has been formulated. Safety assessment will be conducted through vital signs, physical examination, electrocardiogram and laboratory tests to ensure the safety of the subjects.

In summary, the risk-benefit ratio of this clinical study is good.

3 Study objectives and endpoints

Objective	Endpoint
**Main Objective**	**Primary Endpoint**
To evaluate the safety and tolerability of JMT101 in combination with Afatinib or Osimertinib in stage IIIB or IV non-small cell lung cancer (NSCLC) patients with EGFR exon 20 insertional mutations.	Adverse events, physical examination, vital signs, laboratory tests (including routine blood, blood biochemistry, routine urine, coagulation), and ECG.
**Secondary Objective**	**Secondary Endpoints**
• To evaluate the efficacy of JMT101 in combination with Afatinib or Osimertinib in stage IIIB or IV NSCLC patients with EGFR exon 20 insertional mutations, so as to provide a basis for the recommended dosing regimen for subsequent studies.<br><br>• To evaluate the pharmacokinetic profile of JMT101.<br><br>• To evaluate the immunogenicity of JMT101.<br>• To analyze possible correlations between biomarkers and clinical outcomes.	• Efficacy endpoints: objective response rate (ORR, assessed by IRC and investigators according to RECIST 1.1 criteria, respectively), duration of response (DOR), disease control rate (DCR), progression-free survival (PFS), and overall survival (OS).<br>• Pharmacokinetic parameters: including but not limited to $AUC_{0\text{-}t}$, $AUC_{0\text{-}inf}$, C_{max}, T_{max}, $t_{1/2}$ and CL.<br>• Occurrence of anti-drug antibodies and neutralizing antibodies.<br>• Tumor-associated biomarkers are explored to analyze possible correlations between biomarkers and clinical outcomes.

4 Trial design

4.1 Overall Design

This is a multi-center, open-label, dose-escalation phase Ib clinical study in stage IIIB or IV NSCLC patients, which aims to evaluate the safety, tolerability and efficacy of JMT101 in combination with Afatinib or Osimertinib in patients with stage IIIB or IV NSCLC EGFR exon 20 insertional mutations, to provide a basis for the recommended dosing regimen for

subsequent studies, to evaluate the pharmacokinetic profile and immunogenicity of JMT101, and to explore tumor-associated biomarkers.

This study is divided into two stages: the first stage (Stage I) is a dose escalation study and the second stage (Stage Ⅱ) is a dose expansion study.

Stage I dose escalation study:

This study adopts a combination therapy regimen, which divides the patients into Group A and Group B according to different combined drugs: Group A is treated with JMT101 in combination with Afatinib and Group B is treated with JMT101 in combination with Osimertinib. Each group is divided into two cohorts according to the administered dose, and a total of 4 cohorts are established as follows:

A1 cohort: JMT101 6 mg/kg, intravenous drip, Q2W + Afatinib 30 mg, oral, QD;
A2 cohort: JMT101 6 mg/kg, intravenous drip, Q2W + Afatinib 40 mg, oral, QD;
B1 cohort: JMT101 6 mg/kg, intravenous drip, Q2W + Osimertinib 80 mg, oral, QD;
B2 cohort: JMT101 6 mg/kg, intravenous drip, Q2W + Osimertinib 160 mg, oral, QD;

The dose escalation follows the “3 + 3 principle”. Each cohort includes 3-6 subjects to observe safety and tolerability. Subjects with asymptomatic central nervous system (CNS) metastasis or meningeal metastasis are enrolled in Group A or B at the discretion of the investigator, and the rest of the subjects are enrolled into Group A and B in sequence.

Subjects are first enrolled in A1 and B1 cohorts, and each of the cohorts shall have at least 3 evaluable subjects. Subjects can be enrolled in higher dose cohorts (A2 or B2) only if no dose-limiting toxicity (DLT) is observed in 3 subjects in cohorts A1 or B1 during the DLT observation period (Cycle 1, Day 1 to 28). If DLT occurs in 1 of 3 subjects in one cohort, 3 additional subjects need to be added to the same cohort (the cohort has 6 evaluable subjects). If no DLT occurs in the 3 additional subjects, the subjects will continue to be enrolled in the higher dose cohort; if DLT occurs in 1 or more of the 3 additional subjects or in 2 or more of the 6 subjects in total, the planned higher dose cohort will not be conducted. If DLT occurs in 2 of 3 subjects in one cohort, the planned higher dose cohort enrollment will not be conducted.

If drug intolerance occurs in the initial dose cohort (A1 or B1) in a group, the investigator and sponsor are required to discuss jointly to decide whether to proceed with a cohort that is one dose level down from JMT101 (i.e., JMT101 4 mg/kg Q2W, with no change in combined drug dose) or to stay in the initial dose cohort and continue enrolling subjects until excessive toxicity occurs or early discontinuation of the study in the group. If the safety and tolerability of this dose group remains good when the dose is escalated to the predetermined maximum dose (cohort A2 or B2), the decision to explore JMT101 up one dose level cohort (i.e., JMT101 8 mg/kg Q2W with no change in combined drug dose) may be discussed between the investigator and sponsor.

The previous dose level of the dose-limiting toxicity (DLT) dose level is defined as maximum tolerated dose (MTD). At least 6 subjects are enrolled and evaluated in the MTD dose group.

Stage II dose expansion study:

According to the safety, tolerability and efficacy data obtained in Stage I, the dose expansion study is carried out for the target dose cohort. All cohorts with good safety and tolerability in Stage I (up to 4 cohorts) are selected in which a certain number of additional subjects are included (plus subjects in the dose escalation stage) to further explore safety,

tolerability, pharmacokinetic profile and anti-tumor activity. Subjects with asymptomatic central nervous system (CNS) metastasis or meningeal metastasis are enrolled in Group A or B at the discretion of the investigator, and the rest of the subjects are enrolled into each cohort in a certain order. Safety and efficacy data are monitored periodically during the course of the study. Considering the subject benefits, if a cohort shows clear evidence of treatment disadvantage, the enrollment into the cohort should be closed in advance to avoid more subjects receiving ineffective or low effective treatment; if a cohort shows clear evidence of treatment benefits, the recruiting number may be increased in that cohort. A minimum of 12 subjects and a maximum of 200 subjects are expected to be enrolled in the dose escalation study and the dose expansion study.

For all the above subjects, every 4 weeks (28 days) is considered as a treatment cycle during the treatment period, and treatment continues until one of the following occurs: progressive disease, unacceptable toxicity, subject’s withdrawal request, or absence of further benefit from treatment judged by the investigator, whichever occurs first. The dosing regimen may be adjusted based on the subject’s toxicity reaction (the dosing regimen are not allowed to be adjusted for the subjects during the DLT observation period in Stage I). Refer to Section 6.1.3 for specific dose modification principles.

Safety inspections are performed during treatment as required by the protocol, and tumor assessments are performed at the end of Cycle 1 and the end of every two cycles thereafter (Cycle 3, Cycle 5 ....) (If tumor-associated symptoms worsen and the investigator deems it necessary, the duration of tumor assessment may be shortened). After the last dose of investigational drug, subjects should be followed up for survival every 8 weeks, and for adverse events still present at the end-of-study visit.

4.2 Definition of Dose-Limiting Toxicity

DLT is defined as: one or more of the following toxic reactions judged to be reasonably related (related, probably related, and possibly related) to the investigational drug that occurs within Cycle 1 (Day 1 to 28) of study dosing.

(1) Non-hematologic toxicity:

- Grade 4 rash or Grade 3 rash that leads to 4 weeks of suspension, or Grade 3 rash with severe infection.
- Grade 4 diarrhea or Grade 3 diarrhea that has not resolved after 2 weeks of suspension, or Grade 3 diarrhea that reappears after appropriate supportive therapy.
- Any non-hematologic toxicity of grade 3 or greater other than rash and diarrhea (nausea/vomiting, constipation and electrolyte imbalance are considered as DLT only if they remain ≥ grade 3 after appropriate supportive treatment).
- Development of interstitial pneumonia or pulmonary fibrosis.

(2) Hematologic toxicity:

- Grade 4 neutropenia that persists for more than 5 days.
- Febrile neutropenia (ANC < 1.0 x 10^9/L with temperature of 38.3°C (axillary temperature) in a single measurement or temperature ≥ 38°C (axillary temperature) for more than one hour).
- Grade 3 neutropenia with evidence of infection.
- Grade 3 thrombocytopenia with clinically significant hemorrhage.
- Grade 4 thrombocytopenia.
- Grade 4 anaemia (life threatening).

(3) Other toxic reactions that, in the judgment of the investigator, should result in permanent discontinuation of the investigational drug.

All adverse events will be graded according to the National Cancer Institute Common Terminology Criteria for Adverse Events (NCI-CTCAE) Version 5.0.

4.3 Definition of Maximum Tolerated Dose

The previous dose level of the dose-limiting toxicity (DLT) dose level is defined as MTD. At least 6 subjects are enrolled and evaluated in the MTD dose group.

4.4 Definition of End of Study

A subject is considered to have completed the study if he or she has completed all stages of the study, including the last visit or the last planned procedure listed in the study schedule (SoA, Section 1.3).

End of study is defined as the completion of the last visit or the completion of all steps listed in the study schedule (SoA).

5 Study population

5.1 Inclusion Criteria

1. At age of ≥18 years old, regardless of gender.
2. Naive or treated patients who have a histologically or cytologically confirmed diagnosis of stage IIIB or IV NSCLC and are not suitable for radical surgery or radiotherapy, with confirmed EGFR exon 20 insertion mutations (including duplication mutations).
   *NSCLC staging refers to the 7th Edition of TNM Classification of Lung Cancer (revised). See Appendix V for details.
3. The presence of at least one measurable lesion at baseline defined by RECIST 1.1 criteria.
4. ECOG performance status score: 0 or 1.
5. Estimated survival time ≥ 3 months.
6. Major organ and bone marrow function within 7 days prior to treatment meets the following criteria (no transfusion, EPO, G-CSF, GM-CSF, or other supportive therapies within 7 days prior to investigational drug administration):

System	Laboratory test value
**Blood routine**	
Absolute neutrophil count (ANC)	≥1.5×10^9/L
Platelet	≥90×10^9/L
Haematoglobin	≥90 g/L or ≥5.6 mmol/L
**Kidney**	
Serum creatinine	≤1.5 × upper limit of normal (ULN)
**Liver**	
Total bilirubin	≤1.5×ULN<br>Patients with liver metastasis: ≤ 3 × ULN
AST and ALT	≤2.5×ULN<br>Patients with liver metastasis: ≤ 5×ULN
**Coagulation**	
International normalized ratio (INR) or prothrombin time (PT)	≤1.5×ULN
Activated partial thromboplastin time (APTT)	≤1.5×ULN

7. Females of childbearing potential have a negative blood pregnancy test within 7 days prior to administration of investigational drug; any male and female patients of

childbearing potential must agree to use effective contraceptive methods during the entire study period and within six months after trial completion. A patient of childbearing potential, in the judgment of the investigator, refers to one who is biologically capable of giving birth to a child as well as having a normal sexual life. Female patients of no childbearing potential (i.e., at least 1 of the following criteria is met):

Having undergone hysterectomy or bilateral oophorectomy, or

Medically confirmed as ovarian failure, or medically confirmed as post-menopause (at least 12 consecutive months of menopause without pathological or physiological causes).

8. Subjects are required to give informed consent to the study and voluntarily sign a written informed consent form prior to the trial.

5.2 Exclusion Criteria

1. Previously treated with EGFR monoclonal antibody targeted therapy.
2. Having received anti-tumor therapies such as chemotherapy, biologic therapy, targeted therapy, or immunotherapy within 4 weeks prior to first dose of investigational drug, oral small molecule targeted drugs, within 2 weeks prior to first dose of investigational drug or within 5 half-lives of the known drug (whichever is longer); for radiotherapy, within 2 weeks prior to first dose of investigational drug.
3. Treated with another clinical investigational drug within 4 weeks prior to first dose of investigational drug.
4. Major organ surgery (excluding aspiration biopsy) or significant trauma within 4 weeks prior to first dose of investigational drug.
5. Known hypersensitivity reactions or intolerances to any component of the investigational drug or its excipients.
6. Use of strong or moderate inducers of CYP3A4, strong inducers and inhibitors of P-gp used within 14 days prior to first dose of investigational drug. See Appendix IV for details.
7. Adverse effects due to prior anti-tumor therapy that have not returned to a CTCAE 5.0 grade of ≤1 (except for toxicities such as alopecia, which in the judgment of the investigator are of no safety risk).
8. CNS metastasis or meningeal metastasis with clinical signs.
9. History of autoimmune disease, immunodeficiency, including positive HIV, or other acquired or congenital immunodeficiency diseases, or history of organ transplantation.
10. Active hepatitis B (hepatitis B virus titer > 1000 copies/mL or 200 IU/mL); hepatitis C virus, syphilis infection.
11. History of serious cardiovascular diseases, including but not limited to:

- Having complete left bundle branch block or third-degree atrioventricular block.
- History of myocardial infarction, angioplasty, coronary artery bypass.
- Patients with prolonged QT/QTc interval on the ECG at baseline (QTCF > 450 ms for males and > 480 ms for females).
- Severe cardiac arrhythmias that, in the judgment of the investigator, has an impact on this trial.
- Left ventricular ejection fraction (LVEF) ≤50% determined by echocardiography (ECHO) or multi-gated acquisition (MUGA) technique at baseline.
- New York Heart Association (NYHA) Class II or above cardiac failure.
- Poorly controlled hypertension (BP greater than or equal to 150/95 mmHg despite optimal treatment).
- Previous or current cardiomyopathy that, in the judgment of the investigator, has an

impact on this trial.

12. Inability to swallow the drug orally, or presence of a condition that, in the judgment of the investigator, severely impairs gastrointestinal absorption.
13. Other malignancies diagnosed within 5 years prior to first dose of investigational drug, except effectively treated skin basal cell carcinoma, skin squamous cell carcinoma and/or effectively resected in situ cervical cancer and/or breast cancer.
14. Any previous history of interstitial lung disease, drug-induced interstitial lung disease, radiation pneumonia requiring steroid therapy, or any evidence of clinically active interstitial lung disease.
15. History of other serious systemic diseases who, in the judgment of the investigator, are not suitable for participation in the clinical trial.
16. Known alcohol or drug dependence.
17. Previous history of definite neurological or psychiatric disorders, including epilepsy or dementia.
18. Pregnant or lactating women.
19. Not suitable to participate in this clinical study for other reasons, in the opinion of the investigator.
20. Known to carry EGFR exon 20 insertion mutations along with other EGFR mutations corresponding to approved EGFR-TKI therapeutic drugs (i.e., exon 19 del, L858R, T790M, L861Q, G719X or S768I, where X is any other amino acid).
21. Prior use of proposed drugs for NSCLC with EGFR exon 20 insertion mutations such as TAK-788, poziotinib or JNJ-61186372; patients with effective prior EGFR-TKI therapy (including optimal efficacy CR, PR, or SD for more than 6 months); patients in the combined Osimertinib cohort who have used immunotherapy agents such as PD-(L)1 monoclonal antibody, etc. within the previous 3 months.

5.3 Screening Failure

Subjects are allowed to be retested up to two times at the study site during screening if their laboratory tests or ECG results fails to meet the protocol requirements. Subjects who are unable to enter the study within 28 days from the date of signing the ICF because they do not meet the inclusion or meet the exclusion criteria are recorded as screening failure. Subjects who fail screening may be re-screened after appropriate treatment or observation as determined by the investigator. Re-screened subjects will be assigned a new screening number and a new ICF will be signed. Each subject has a maximum of one re-screening opportunity.

6 Study intervention

6.1 Investigational drug

6.1.1 Investigational Drug Description

1. Generic name: Recombinant Humanized Anti-epidermal Growth Factor Receptor (EGFR) Monoclonal Antibody Injection
Abbreviation: JMT101
English name: Recombinant Humanized Anti-EGFR Monoclonal Antibody Injection
Active ingredient: Recombinant Humanized Anti-epidermal Growth Factor Receptor (EGFR) Monoclonal Antibody
Strength: 100 mg/10.0 mL/vial
Stability and storage conditions:
The investigational drug is stable for at least 36 months at 2-8℃, 2 months at room

temperature, allowing for 5 repeated freeze-thaws, and oscillation.
Storage conditions: 2-8℃, protected from light; no freezing. Shelf life: 36 months (tentative)

2. Generic name: Afatinib Dimaleate Tablets
Trade name: Giotrif®
English name: Afatinib Dimaleate Tablets
Active ingredient: Afatinib Dimaleate
Strength: 30 mg/tablet, 40 mg/tablet
Stability and storage conditions:
Shelf life: 36 months
Storage condition: no more than 25℃.

3. Generic name: Osimertinib Mesylate Tablets
Trade name: TAGRISSO®
English name: Osimertinib Mesylate Tablets
Active ingredient: Osimertinib Mesylate
Strength: 80 mg/tablet
Stability and storage conditions:
Shelf life: 36 months
Storage condition: room temperature

6.1.2 Dose Regimen of Investigational Drug

- JMT101: The administered dose is calculated according to 6mg/kg based on the body weight before the 1st dose (the error from the theory is allowed ≤±5%). If the subject's body weight in subsequent treatments does not exceed 10% compared with the weight before the 1st dose, the dose will not be recalculated. The route of administration is intravenous drip and the frequency of administration is every 2 weeks (Q2W). The required amount of this product is drawn accurately, diluted to 100 mL with 0.9% sodium chloride injection, mixed well and administered intravenously for 90 min (±15 min) at a constant rate. If the first dose is tolerable, the infusion time can be shortened to 60 min (60-105 min) in subsequent treatments; the maximum infusion rate should not exceed 10 mg/min. The infusion line is rinsed with 0.9% sterile sodium chloride solution at the end of infusion. This product is not allowed for intravenous bolus or rapid injection. If the drug cannot be used in time after preparation, it should be left at room temperature for no more than 4 h; and at 2-8°C for up to 24 h. Dose modification may be made according to the protocol based on the subject's toxicity reaction.
- Afatinib: 30 mg or 40 mg, oral, once daily (QD). This product should not be taken with food. It should be taken at least 3 h after eating or at least 1 h before eating. The tablet should be swallowed in whole with water. Dose modification may be made according to the protocol based on the subject's toxicity reaction.
- Osimertinib: 80mg or 160mg, oral, once daily (QD). If one dose is missed, the missed dose should be taken unless the next dose is within 12 h. This product should be taken at the same time each day as possible, either with a meal or on an empty stomach. Dose modification may be made according to the protocol based on the subject's toxicity reaction.

Dosing regimen:

A1 cohort: JMT101 6 mg/kg, intravenous drip, Q2W + Afatinib 30 mg, oral, QD;
A2 cohort: JMT101 6 mg/kg, intravenous drip, Q2W + Afatinib 40 mg, oral, QD;
B1 cohort: JMT101 6 mg/kg, intravenous drip, Q2W + Osimertinib 80 mg, oral, QD;
B2 cohort: JMT101 6 mg/kg, intravenous drip, Q2W + Osimertinib 160 mg, oral, QD;

Every 4 weeks (28 days) is considered a treatment cycle during the treatment period, and treatment continues until one of the following occurs: progressive disease, unacceptable toxicity, withdrawal of consent, or absence of further benefits in the opinion of the investigator, whichever occurs first. The dosing regimen may be adjusted based on the subject’s toxicity reaction (the dosing regimen are not allowed to be adjusted for the subjects during the DLT observation period in Stage I). Refer to Section 6.1.3 for specific dose modification principles.

6.1.3 Principles of dose modification and toxicity treatment

6.1.3.1 Principles and methods for the treatment of JMT101-related toxic and side effects

The following adverse events related to JMT101 in clinical use will be handled in accordance with the corresponding principles.

Infusion reaction

Infusion reactions in clinical use mostly occur within one hour of the start of the first infusion and are mainly manifested as symptoms such as fever, chills, difficulty breathing, bronchospasm, and possible allergic reactions, such as hypotension and rash. Once the above adverse reactions occur during the infusion, the investigator will determine the severity and whether to stop the infusion administration. Treatment includes oxygen inhalation, use of conventional antipyretic analgesics (e.g. acetaminophen), anti-allergic drugs (e.g. diphenhydramine), corticosteroids.

Local reaction at the administration site

The investigator will evaluate the subject’s injection site for intravenous administration prior to dosing. If a local reaction occurs after administration, it will be evaluated by the investigator according to CTCAE version 5.0 and recorded as an AE.

Principles and methods of skin toxicity treatment

Skin toxicity is the most common toxic and side effect of anti-EGFR therapy. Please refer to the treatment principles and methods of marketed similar drugs[1].

Prophylactic oral antibiotics are required during the study. Subjects are asked to take oral minocycline capsules regularly from the first dose: 0.1g twice daily for 8 weeks. The dosage and duration of treatment could be adjusted according to the subject’s specific condition when necessary. If the subject is allergic to tetracyclines, the investigator may use other drugs for prophylactic treatment according to the subject’s specific condition.

Subjects are advised to apply sunscreen twice daily and to avoid direct sunlight during the investigational drug treatment. Subjects should wear loose-fitting, comfortable non-chemical synthetic material clothing, shoes and socks to avoid skin damage. Application of alcohol-free moisturizer on the face and neck is recommended.

If Grade 1-2 skin toxicity occurs during the study, topical antibiotics and hydrocortisone ointment may be used for treatment in addition to oral antibiotics. When Grade 3 or higher skin toxicity is reached, the investigational drug is discontinued, and other treatments are the same as Grade 1-2. When JMT101 administration is suspended, patients may start JMT101 treatment again if their skin toxicity has improved (< Grade 3) or if administration is delayed

for no more than 4 weeks. Patients with severe skin toxicity are advised to consult a dermatologist to assist with treatment or to seek dermatological treatment if necessary.

6.1.3.2 Dose Modifications and Treatment Delays

Dose modifications are allowed in this study except for the DLT observation period in the dose-escalation stage. In case of < Grade 3 adverse reactions, symptomatic supportive treatment can be provided, and study treatment can be continued without dose modification. All study treatment should be suspended in the event of Grade ≥3 adverse reactions that are judged by the investigator to be a safety risk: if Grade 3 or higher adverse reactions have improved to Grade 0 to 2 within 4 weeks after study treatment suspension, resume administration at the original dose (in case of first occurrence of a Grade ≥3 adverse reaction) or resume administration with a dose modification (when a Grade ≥3 adverse reaction occurs for the second or more times, or when it occurs for the first time but is judged by the investigator to be a greater risk when administered at the original dose); if the study treatment is suspended for more than 4 weeks and Grade 3 or higher adverse reactions have not improved to Grade 0 to 2, the study treatment will be terminated, or the investigator and the sponsor will discuss and decide whether to continue the treatment. If Grade ≥ 3 toxicity occurs despite two dose modifications, discontinuation of treatment is considered or a decision to continue treatment will be made by the investigator and the sponsor.

The dose modification rules are shown in Figure 2 and Figure 3. The dose of Afatinib or Osimertinib is preferentially reduced (the dose of Afatinib is reduced from 40 mg qd to 30 mg qd, the dose of Osimertinib is reduced from 160 mg qd to 80 mg qd, and the dose of JMT101 remains unchanged). If the dose of Afatinib or Osimertinib can no longer be further reduced due to specification limitations (Afatinib: 30 mg/tablet qd, Osimertinib: 80 mg/tablet qd), the JMT101 dose will be reduced by one dose level at a time according to 6 mg/kg-4 mg/kg-2 mg/kg (q2w), up to a maximum of two dose levels.

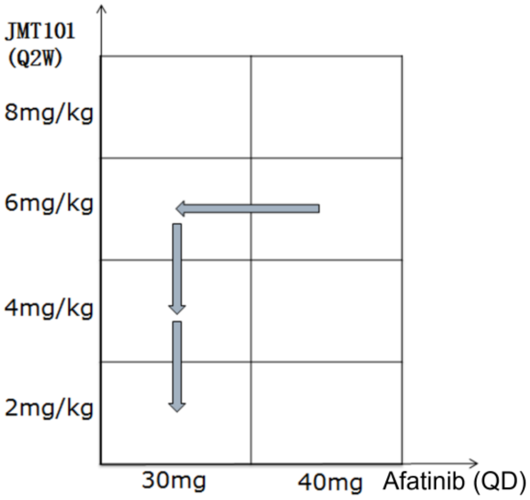

Figure 2: Dose modification rules of JMT101 combined with Afatinib

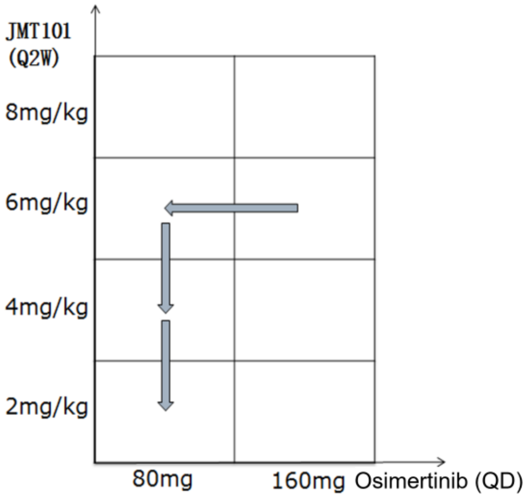

Figure 3: Dose modification rules of JMT101 combined with Osimertinib

6.2 Preparation/Handling/Storage/Disposal of Investigational Drug

6.2.1 Drug receipt and accountability

The sponsor is responsible for shipping investigational drugs to the study site and the investigator/pharmacist signs to confirm receipt.

Investigational drugs should only be used in this study and should only be managed by a specified person authorized by the investigator. The investigator/pharmacist responsible for drug management dispenses, recalls, destroys investigational drugs according to the study process and keeps accurate records. Used investigational drugs and their packaging will be disposed of and destroyed as medical waste at the study site, and unused or expired drugs will be handed over to the sponsor for recycling and destruction.

6.2.2 Dosage form, appearance, packaging and labeling

Investigational drug: JMT101

Dosage form: injection

Packaging: plastic vial

labeling:

- Protocol No.
- Drug No.
- Drug name
- Strength
- Batch No.
- The shelf life, which should be in the same label format as the test report of the investigational drug.
- Storage conditions
- Instructions
- Indicate “Keep out of reach of children”
- Indicate “for this clinical study only”
- Indicate “provided by Shanghai JMT-Bio Inc.”

6.2.3 Product storage and stability

The investigational drug is stable for at least 36 months at 2~8°C, 2 months at room temperature, allowing for 5 repeated freeze-thaws, and oscillation.

Storage conditions: 2-8℃, protected from light; no freezing. Shelf life: 36 months (tentative)

6.2.4 Preparation

All investigational drugs will be prepared by appropriately trained and experienced personnel in accordance with the study documents.

6.3 Pharmacokinetics, immunogenicity, biomarker test

6.3.1 Pharmacokinetic test

Stage I dose escalation study:

Approximately 2.5 mL of blood will be collected within 30 minutes before the 1st and 3rd dose and immediately after the end of the dose (+2 min), at 4 h (±15 min), 8 h (±30 min), 24 h (±1 h), 48 (±2 h), 96 h (±4 h), 168 h (±7 h), and 240 h (±12 h) after the 1st and 3rd dose.

Approximately 2.5 mL of blood will be collected each within 30 minutes before and immediately after the 2nd, 4th, and 5th doses (+2 min) and 30 days (±3 days) after the last dose.

Stage II dose expansion study:

Approximately 2.5 mL of blood will be collected within 30 minutes before the 1st and 3rd dose and immediately after the end of the dose (+2 min), at 8 h (±30 min), 24 h (±1 h), and 168 h (±7 h) after the 1st and 3rd dose.

Approximately 2.5 mL of blood are collected within 30 minutes before and immediately after the end of the 2nd, 4th, and 5th doses (+2 min) and 30 days (±3 days) after the last dose.

6.3.2 Immunogenicity detection

Before the 1st dose, 15 days (±1 day) (before the 2nd dose), 29 days (±1 day) (before the 3rd dose) and 57 days (±3 days) (before the 5th dose) after the 1st dose, and at the last visit (30±3 days after the last dose). Blood will be collected within 30 minutes prior to each dose, approximately 3.5 mL per collection.

Samples will be first tested for anti-drug antibodies. Samples that are tested positive for anti-drug antibodies need to be further tested for neutralizing antibodies.

6.3.3 Biomarker detection

Diagnostic biomarker detection:

For EGFR exon 20 insertion mutation detection, one of the following conditions should be met:

(1) For those who cannot provide the test report of EGFR exon 20 insertion mutation, NGS or qPCR should be used to detect EGFR exon 20 insertion mutations in tumor tissue DNA or peripheral blood specimens of patients before enrollment. Patients with verified mutations will be enrolled.

During the screening period, subjects are encouraged to preferentially provide sufficient tumor tissue samples for EGFR exon 20 insertion mutation detection, but it is not mandatory. If this is not feasible or if subjects refuse to provide, 10 mL of peripheral blood specimen needs to be collected from the subjects for EGFR exon 20 insertion mutation detection.

(2) For those who can provide the test report of EGFR exon 20 insertion mutation, they may be enrolled only after the investigator has reviewed the third-party test report and confirmed the mutation.

Pharmacodynamic biomarker detection:

Ten (10) mL of blood is collected before the first dose (within 30 min), at the time of first efficacy assessment and disease progression, respectively, and plasma free DNA are extracted and analyzed for the correlation between efficacy and biomarkers using NGS.

6.4 Methods to reduce deviations: randomization and blinding

This study is a non-randomized, open-label clinical study.

6.5 Study intervention compliance

The use of investigational drug should be strictly in accordance with the clinical protocol, and its use in each subject should be recorded in the eCRF.

The research staff at the study site should conduct a drug accountability at the time of drug distribution, recycling and destruction, and sign the drug transfer, handover and destruction documents for confirmation.

6.6 Concomitant Medication and Treatment

6.6.1 Prohibited drugs and treatments

Any other anti-tumor therapy (including chemotherapy, radiotherapy, targeted agents, immunotherapy, biotherapy, etc.) and all other clinical investigational drugs are prohibited during the study period.

The use of traditional Chinese medicines with anti-lung cancer indications approved by the National Medical Products Administration (NMPA) is prohibited during the study period.

The use of strong or moderate inducers of CYP3A4, strong inducers and inhibitors of P-gp is prohibited from 14 days prior to first dose of investigational drug until the completion of the study. See Appendix IV for details.

6.6.2 Permitted Drugs and Treatments

The following drugs or treatments are permitted as appropriate during the study period:

(1) Long-term medication required for comorbidities (e.g. hypertension, diabetes);

(2) Necessary supportive treatment for drug toxicity reactions or clinical indications given by the investigator for the benefit and safety of the subject;

(3) Patients with bone metastases may be treated with bisphosphonates or anti-RANKL monoclonal antibodies. Small area **(**the radiotherapy area must be <5% bone marrow area**)** palliative radiotherapy is allowed if the painful bone metastasis cannot be effectively controlled by systemic therapy or local analgesia;

(4) Patients with intracranial progression only may receive local radiotherapy if it is in the subject's best interest to continue study treatment as assessed by the investigator.

7 Discontinuation of Study Intervention and Subject Withdrawal

7.1 Discontinuation of Study Intervention

Discontinuation of study intervention is defined as discontinuation of treatment for the subject decided by the investigator for any reason. Discontinuation of study intervention does not mean study discontinuation. The subsequent study procedures shall be completed according to the protocol.

7.2 Subject Withdrawal

Subjects may request to withdraw from the study at any time.

The investigator may decide discontinuation or subject withdrawal from the study for any of the following reasons:

- Pregnancy
- Poor compliance to the study intervention
- Clinical adverse events (AEs), laboratory abnormalities or other medical conditions, where continued participation in the study will not serve for the best benefits of the subject
- Disease progression requiring discontinuation of study intervention
- Other discontinuation decided by the investigator

For all the subjects discontinuing or withdrawing from the study, the reason for discontinuation or withdrawal should be recorded, and if possible, the subjects should be contacted to complete the end-of-treatment evaluation, safety follow-ups and other visits.

End-of-treatment visit

The end-of-treatment date is the date of discontinuation of treatment for the subject decided by the investigator for any reason. The end-of-treatment visit needs to be completed within 7 days after the last dose. If a decision is made to discontinue treatment at one visit (e.g., due to disease progression), that visit can also be considered as an end-of-treatment visit. If a patient fails to return to the study site for an EOT visit, this patient should be contacted to collect any adverse events that occurred. Patients whose treatment is interrupted or permanently discontinued due to adverse events (including abnormal laboratory results) must be followed up until the event recovers or stabilizes, whichever occurs earlier.

Safety Follow-up

All subjects, including those who withdraw early from the study, should complete safety follow-up visits (i.e., assessment of AE and/or SAE, and concomitant medication) 30 days (±3 days) after the last dose or prior to new anti-tumor therapy, whichever occurs first. Patients whose treatment is interrupted or permanently discontinued due to adverse events (including abnormal laboratory results) must be followed up until the event recovers or stabilizes, whichever occurs earlier.

Survival follow-up

Follow-up for progression-free survival: For patients who discontinued the treatment before disease progression, tumor assessment is required every 8 weeks ± 7 days after the last dose (if treatment is discontinued due to disease progression, no lesion measurement or efficacy evaluation is required) until disease progression, loss to follow-up, death or replacement of treatment regimen (whichever occurs first).

Follow-up for overall survival: The subjects who have confirmed disease progression or receive new anti-tumor treatment will enter the survival follow-up period, and the survival state and the subsequent anti-tumor treatment should be collected by clinical follow-up or telephone follow-up every 8 weeks ± 7 days after the end of treatment until death, loss to follow-up or end of study (whichever occurs first).

7.3 Lost to follow-up

If subjects do not return to the study site for the scheduled visits and cannot be contacted by the study site personnel, the subjects will be regarded as lost to follow-up.

If subjects do not return to the study site for the specified study visits, the following actions must be taken:

- The study site should attempt to contact the subject to rearrange the missed visits, to explain the importance of compliance with the visit schedule to the subject, and to confirm whether the subject is willing to and/or should continue participation in the study.
- Before the subject is regarded as loss to follow-up, the investigator or the designee should attempt to contact the subject again (if possible, dial more than three times the mobile phone number, fixed phone number, WeChat number or other immediate contact numbers of the subject or his/her family member during different time periods. If necessary, send the WeChat or SMS reminder, or send a registered mail to the subject’s latest mailing address, or use the local effective contact information). These attempts to contact the subject should be documented in the subject’s medical record or study file.
- If the subject still cannot be contacted, he/she will be regarded as withdrawal from the study, and the reason for withdrawal is lost to follow-up.

8 Study evaluation and procedure

8.1 Demographic Characteristics and Background Evaluation

8.1.1 Demographic characteristics

The demographic data and baseline data will be collected on Day -28 to Day -1, including the name, gender, age and nationality.

8.1.2 Body weight and height

The height will be measured in the screening period only, and the body weight will be measured at each visit in the screening period and treatment period (prior to JMT101 administration) and at the end-of-treatment visit.

8.1.3 Medical history and other past medical histories

Medical history refers to the previous and contaminant disease information of clinical significance (or sign and symptom, if not confirmed) before signing of the Informed Consent Form (ICF), including the past medical history and the present medical history. The tumor disease history includes the diagnosis time, pathological type, diagnosis staging and screening staging. All the medical histories related to the subject’s tumor diagnosis must be traced back to the initial diagnosis. In addition, any clinically significant abnormality found in the history of smoking, alcoholism, drug abuse and surgery or related examinations during the screening period should also be recorded in the eCRF as past medical history.

The gene test report of EGFR 20 exon insertion mutation is required when the subject is enrolled into the study. Any other combined mutations should also be recorded accordingly.

8.1.4 Prior medication/concomitant medication and therapy

Prior medication refers to any drug used within 28 days prior to administration of investigational drug.

Concomitant medication/therapy refers to two or more than two drugs and therapies used concomitantly. In the study, it is defined as any medication/therapy administered at the same time or during the same period from the first dose of investigational drug until 30 days after the last dose.

At each visit during the study from the initial screening to the end of study, the investigator should ask the subjects for the medication/therapy related information since the previous visit. Any prior medication or concomitant therapy should be recorded in the eCRF. The records of concomitant medication include the following information: drug/non-drug name, reason for medication, dosage, dose unit, as well as frequency, route, start and end dates of administration; the records of concomitant non-drug therapy shall include the non-drug treatment name, treatment description, reason for treatment, treatment start date and end date. If the reason for concomitant medication/therapy meets the definition of AE, the related information should also be recorded in the subject's eCRF.

8.1.5 Prior anti-tumor therapy

It includes surgery, medication and radiotherapy, and the following data should be collected: start and end dates of treatment, name of treatment regimen, dose, unit, frequency, the best response and reason for discontinuation of treatment. Particularly, if the subjects who previously have received Afatinib, Osimertinib, Poziotini, TAK-788 or JNJ-61186372 are enrolled in the study, the related medication history must be recorded.

8.1.6 Serum virology and syphilis

Serum virology: Hepatitis B five-index tests (HBsAg, HBsAb, HBeAg, HBeAb, HBcAb), HBV-DNA quantitative test, HCV-Ab test, HCV-RNA quantitative test, HIV-Ab test, and treponema pallidum antibody test (RPR or TRUST).

They shall be only conducted during the screening period. The serum virology and syphilis assessment test shall be conducted at the study site. The subjects with positive HIV-Ab or syphilis test results during the screening period cannot participate in the study. The subjects with positive HBsAg or HCV-Ab should receive the HBV-DNA or HCV-RNA quantitative test.

8.1.7 Tumor assessment

Anti-tumor efficacy will be evaluated according to the RECIST 1.1.

The imaging examinations during the screening period include CT scans of the chest, abdominal and pelvic CT scan (preferably enhanced CT; in case of allergy to the contrast medium or contraindication, plain CT scan or abdominal and pelvic MRI scan is acceptable), head MRI scan (in case of contraindication, head CT scan is acceptable) and bone scan (ECT). If clinically indicated, appropriate methods shall be used to check any other known or suspected lesions, e.g. neck CT scan.

The tumor assessment during the screening period accept the results obtained within 4 weeks prior to the administration (including the imaging examination results prior to the signing of ICF).

During the screening period, the investigator should select an appropriate tumor assessment method based on the condition of the subject, and the imaging examination method used in the subsequent tumor assessment should be consistent with that used

during the screening period. If no obvious abnormality is found in the head MRI scan or bone scan during the screening period, the investigator may decide whether to use the head MRI scan or bone scan in subsequent tumor assessment based on the actual condition of the subject.

8.2 Assessment on Safety and Other Items

8.2.1 Laboratory test items

- Routine blood test: red blood cell count (RBC), haemoglobin (HGB), heamatocrit (HCT), mean corpuscular volume (MCV), mean corpuscular haemoglobin (MCH), mean corpuscular-hemoglobin concentration (MCHC), white blood cell count (WBC), platelet (PLT), absolute value of neutrophil (NE#) and absolute value of lymphocyte (LY#);
- Blood biochemistry test: alanine aminotransferase (ALT), aspartate aminotransferase (AST), total bilirubin (TBIL), direct bilirubin (DBIL), alkaline phosphatase (ALP), lactate dehydrogenase (LDH), creatine kinase (CK), gamma-glutamyl transpeptidase (GGT), total protein (TP), albumin (ALB), total cholesterol (TC), triglyceride (TG), urea (UREA) or urea nitrogen, creatinine (Cr), creatinine clearance (Ccr), glucose (Glu), potassium (K^+), sodium (Na^+), chlorine (Cl^-), calcium (Ca^{2+}) and magnesium (Mg^{2+});
- Routine urine test: pH, specific gravity (SG), urine glucose (GLU), urine protein (PRO), urinary red blood cell (RBC), urinary white blood cell (WBC) and urine ketones (KET);
- Coagulation function test: prothrombin time (PT), activated partial thromboplastin time (APTT), thrombin time (TT) and fibrinogen (FBG);

8.2.2 Laboratory test time and evaluation

Laboratory test will be conducted during the screening period, on C1D15, C2D1, C2D15, C3D1, C3D15, C4D1, C4D15 (±2 days), every 14 (±2) days in the subsequent cycles, and at the end-of-treatment visit.

The investigator will review all laboratory results, evaluate the changes from baseline in the laboratory results based on the laboratory reference range, judge the clinical significance of each abnormal laboratory result, and record it as “abnormal, not clinically significant (NCS)” or “abnormal, clinically significant (CS)” (if “normal”, no mark is required). Any laboratory abnormality with any of the following symptoms during the study will be listed as an adverse event and recorded in the eCRF:

- Symptoms directly leading to subject withdrawal from the study;
- Symptoms leading to serious adverse consequences;
- Obvious clinical symptoms;
- Symptoms of clinical significance in the opinion of the investigator.

The reviewed laboratory report will be signed and dated by the investigator.

8.2.3 Physical examination

Physical examination includes the general condition, skin and mucosa, lymph nodes, head and neck, chest, abdomen, spine, muscle and skeleton, nervous system and other parts.

Physical examination will be conducted during the screening period, on C1D15, C2D1, C2D15, C3D1, C3D15, C4D1, C4D15 (±2 days), every 14 (±2) days in the subsequent cycles, and at the end-of-treatment visit. Compared to the baseline, any abnormality of clinical significance should be recorded as an adverse event.

8.2.4 Vital sign

Vital signs include the sitting blood pressure, pulse and body temperature.

The same arm will be used to measure the sitting systolic blood pressure and diastolic blood pressure each time, and the subjects shall rest in the sitting position for at least 5 min before measurement. Standard equipment will be used for recording. Automatic or manual

measurement is acceptable. However, the investigator should continue to evaluate the individual subject using the same method as that used in the first measurement.

Measurement of vital signs will be conducted during the screening period, on C1D1 before administration (-30 min), at 2 h (±30 min), 4 h (±30 min) and 8 h (±30 min) at the end of administration, on C1D15, C2D1, C2D15, C3D1, C3D15, C4D1 and C4D15 (±2 days), every 14 (±2) days in the subsequent cycles, and at the end-of-treatment visit. Compared to the baseline, any abnormality of clinical significance should be recorded as an adverse event.

8.2.5 Performance status

The patient's performance status will be evaluated according to the ECOG Performance Status Scale (see Appendix 1 for details).

8.2.6 ECG examination

ECG examination will be conducted during the screening period, on C1D15, C2D1, C2D15, C3D1, C3D15, C4D1, C4D15 (±2 days), every 14 (±2) days in the subsequent cycles, and at the end-of-treatment visit. If a prolonged QTcF interval occurs in a subject, the investigator may increase the frequency of examination depending on the subject's condition. Subjects should rest for at least 10 minutes before each examination.

8.2.7 Cardiac color ultrasonography

Cardiac color ultrasonography will be conducted during the screening period, at the end of every 3 treatment cycles and at the end-of-treatment visit. The subjects should rest in the supine position for at least 5min before the cardiac color ultrasonography.

8.3 Efficacy Assessment

8.3.1 Endpoints

Objective response rate (ORR): defined as the proportion of patients with complete response (CR) or partial response (PR) (i.e. CR+PR) as the best response as evaluated by the independent review committee (IRC) and the investigator according to the Response Evaluation Criteria in Solid Tumors (RECIST v1.1, see Appendix III for details) during the period from the first dose of investigational drug to withdrawal from the study.

Duration of remission (DOR): defined as the time from the first documentation of objective response to the first documentation of PD or death from any cause before PD, reflecting the duration of ORR.

Disease control rate (DCR): defined as the proportion of patients with CR, PR and stable disease (SD) (i.e. CR+PR+SD) as the best response according to the RECIST v1.1 during the period from the first dose of investigational drug to withdrawal from the study.

Progression-free survival (PFS): defined as the time from initiation of investigational drug to the date of first recorded progression of disease (PD) or death, depending on which occurs first.

Overall survival (OS): defined as the time from initiation of investigational drug to the date of death from any cause.

8.3.2 Efficacy criteria

The investigator will conduct the imaging evaluation according to the Response Evaluation Criteria in Solid Tumors (RECIST v1.1) during the screening period, at the end of the first cycle (± 7 days) and at the end of every two cycles thereafter (the third cycle, the fifth cycle .... ± 7 days) and at the end-of-treatment visit. If the dose is delayed, the corresponding imaging examination time points should be also delayed, but not more than 10 weeks after the last tumor assessment. Subsequent tumor assessment methods should be consistent with those in the screening period as much as possible. If the patient's tumor-associated symptoms worsen during the treatment, tumor assessment may be performed

earlier as determined by the investigator. For patients who discontinue treatment before disease progression, tumor assessment should be performed every 8 weeks ± 7 days after the last dose until disease progression, loss to follow-up, death, or change of treatment regimen (whichever occurs first). If the subject has a tumor assessment within 4 weeks prior to the end-of-treatment visit, no additional tumor assessment is required at the end-of-treatment visit.

In the study, the patients with a first tumor evaluation of CR or PR require a second imaging evaluation at least 4 weeks later for confirmation (it is suggested to add an unscheduled visit 4 weeks later for tumor assessment, and thereafter the tumor assessment may be continued once every two cycles). The patients with disease progression as shown in the imaging examination should withdraw from the study.

In rare cases (e.g. only intracranial progression), the subjects with PD confirmed by imaging examination who are assessed by the investigator to still benefit from treatment may continue receive the study treatment with the subject's consent. The above subjects should be also under efficacy and safety evaluation and continuous risk-benefit assessment according to the study procedures specified in the protocol, until discontinuation of investigational drug and withdrawal from the study due to no benefit as judged by the investigator. The subjects with only intracranial progression are allowed to receive the local radiotherapy and continue participating in the study for collection of extracranial PFS data.

8.4 Pharmacokinetic, immunogenicity and biomarker evaluation

8.4.1 Pharmacokinetic parameters

(1) Stage I dose escalation study

C_{max}, AUC_{0-t}, AUC_{0-inf}, T_{max}, V_z, $t_{1/2}$, CL and $\%AUC_{ext}$ after the first dose; $C_{max,ss}$, $C_{min,ss}$, $AUC_{0-\tau}$, AUC_{0-inf}, $T_{max,ss}$, V_{ss}, $t_{1/2}$, CL, $C_{av,ss}$, DF, Range and $\%AUC_{ext}$ after repeated doses; accumulation index $R_{1ac}=C_{max,ss}/ C_{max}$; accumulation index $R_{2ac}= AUC_{0-\tau}/AUC_{0-t}$.

(2) Stage II dose expansion study

Sparse blood collection will be performed and pooled with other JMT101 concentration data for population pharmacokinetic analysis.

8.4.2 Immunogenicity parameters

To evaluate the immunogenicity of JMT101, samples are first tested for anti-drug antibodies. Samples that are tested positive for anti-drug antibodies should be further tested for neutralizing antibodies.

8.4.3 Biomarker parameters

Tumor-associated biomarkers such as serum free DNA are explored to analyze possible correlations between biomarkers and clinical outcomes.

8.5 Adverse Events and Serious Adverse Events

8.5.1 Definition

Adverse Event (AE)

Adverse event (AE) is any untoward medical occurrence associated with the use of the investigational drug, regardless of the relationship of the occurrence to the investigational drug. An AE can be any unfavorable and unintended sign (including an abnormal laboratory finding), symptom or disease temporally associated with the use of the drug, whether or not considered related to the drug.

Adverse Drug Reaction (ADR)

All noxious and unintended responses to the investigational drug related to any dose should be considered adverse drug reactions. There is at least one reasonable possibility for

the causal relationship between then study drug and the ADR, i.e., the correlation cannot be ruled out.

Treatment Emergent Adverse Event (TEAE)

TEAE is defined as any untoward medical events that newly occurs or gets worse within the study period.

Serious Adverse Event (SAE)

An SAE refers to any untoward medical events that meets any of the following criteria at any dose:

a. Results in death

b. Is life-threatening

The term “life-threatening” in the definition refers to an event in which the subject was at risk of death at the time of the event; it does not refer to an event which might have caused death if it were more severe.

c. Requires inpatient hospitalization or causes prolongation of existing hospitalization, unless hospitalization is for:

• Length of stay less than 24 h

• Planned hospitalization (e.g. elective or scheduled surgery arranged prior to the start of the study; hospitalization is a part of study procedures).

• Hospitalization is not associated with any AE (e.g. social hospitalization for the purpose of short-term care).

d. Results in permanent or significant disability/incapability

Disability refers to serious damage to the normal living ability of individuals.

e. Results in Congenital anomaly or birth defect

f. Other serious or important medical events

Important medical events may not immediately be life-threatening, leading to death or hospitalization, but if medical measures are required to prevent any of the above circumstances, such events will be regarded as serious, e.g. important treatment in the emergency room or allergic bronchospasm at home, dyscrasia or convulsion not leading to hospitalization, drug dependence or addiction.

Suspected Unexpected Serious Adverse Reaction (SUSAR)

Suspected and unexpected serious adverse reaction with the nature and severity of its clinical manifestations beyond the investigator's brochure for the investigational drug, package inserts of marketed drug or summary of product characteristics or other available information.

8.5.2 Adverse event evaluation and classification

The investigator should evaluate and record all the adverse events according to the following standards:

Seriousness

The seriousness of each adverse event must be determined according to the definition of SAE.

Severity

The National Cancer Institute Common Terminology Criteria for Adverse Events (NCI-CTCAE, Version 5.0) will be used to record the severity of adverse events.

The specific grading of CTCAE is as follows:

Grade 1: mild: asymptomatic or mild symptoms; clinical or diagnostic observations only; intervention not indicated.

Grade 2: moderate: minimal, local or noninvasive intervention indicated; limiting age-

appropriate instrumental activities of daily living (instrumental activities of daily living refer to preparing meals, shopping for groceries or clothes, using the telephone, managing money, etc.)

Grade 3: Severe or medically significant but not immediately life-threatening; hospitalization or prolongation of hospitalization indicated; disabling; limiting self-care activities of daily living (self-care activities of daily living refer to bathing, dressing and undressing, feeding self, using the toilet, taking medications, and not bedridden).

Grade 4: Life-threatening consequences; urgent intervention indicated.

Grade 5: Death related to AE.

8.5.3 Causal relationship

Causal relationship between adverse event and investigational drug

The investigator should evaluate the causal relationship between each adverse event and each investigational drug for all the AEs. The evaluation of causal relationship should be completed by the authorized clinician, and except for the judgment whether there is any causal relationship with the investigational drug, the rationale for judgment should also be stated out as detailed as possible.

Judgment approach: During the assessment of the causal relationship between adverse event and investigational drug, the important factors should be considered including:

a. Temporal relationship with the drug administration.

The event should occur after the drug administration. The duration from drug exposure to event occurrence should be evaluated in the clinical background of the event.

b. Responses after drug withdrawal (de-challenge) and after drug resumption (re-challenge)

The patient’s response after de-challenge or re-challenge should be evaluated in combination with the common clinical disease course of related events.

c. Underlying diseases, concomitant diseases, and complications

Each event should be evaluated in combination with the current disease and the subject’s other potential natural history and course of the disease.

d. Concomitant medications or therapies

Other drugs or therapies being received by the patient at the time of adverse event occurs should be investigated to determine whether they might lead to such event.

e. Known reactions to similar drug (clinical/preclinical)

f. Physical/psychological stress exposure

Stress exposure might induce the adverse change in subjects, and provide a more reasonable explanation for such event.

g. Pharmacology and pharmacokinetics of study treatment

The pharmacokinetic profiles (absorption, distribution, metabolism and excretion) of study treatment and the pharmacodynamics in individual subjects should be comprehensively considered.

The relationship between the investigational drug and the AE is classified as: definitely related, probably related, possibly related, doubtfully related and not related. The specific judgment criteria are shown in **错误!未找到引用源。**.

Judgment of relationship between adverse event and investigational drug

	**Definitely related**	**Probably related**	**Possibly related**	**Doubtfully related**	**Not related**
a. Whether there is a reasonable time relationship between the event and the investigational drug?	+	+	+	+	-
b. Whether the event meets the known adverse reaction to similar drug?	+	+	±	-	-
c. Whether the event is alleviated or disappears after drug discontinuation or dose reduction?	+	+	±?	±?	-
d. Whether the event occurs again after drug resumption?	+	?	?	?	-
e. Whether the event can be explained by the effect of concomitant medication, subject’s disease progression or other therapies?	-	-	±	±	+

Note: + denotes Yes; - denotes No; ± denotes undetermined; ? denotes unknown.

If an event is not completely consistent with a certain evaluation result, or a clinical observation cannot be confirmed as an adverse event, it should be strictly evaluated and reported.

Causal relationship with the protocol procedure and clinical operation

Based on the issue of whether there is a “reasonable causal relationship” between the adverse event and the protocol procedure and clinical operation, the evaluation result “related (Yes)” or “not related (No)” should be recorded in the eCRF. Generally, it refers to one of the following circumstances: adverse event arising from the harm/damage caused by any clinical operation (e.g. tissue biopsy); adverse event arising from the drug interruption (e.g. washout), dose reduction or treatment regimen adjustment required by the protocol procedure; or adverse event arising from other prophylactic medications.

Actions taken with the study treatment

All the actions taken with the investigational drug for solving the AEs according to the following categories. The specific actions shall be recorded in the eCRF.

- Dose interruption
- Permanent discontinuation
- Dose reduction
- Dose increased
- Dose unchanged
- Unknown
- Not applicable

Other specific treatment for the adverse event

- None
- Drug treatment
- Other treatments

Outcome

AE outcomes are recorded as follows:

- Recovered
- Relieved/resolved
- Not relieved/not resolved/persistent
- Recovered with sequelae
- Death
- Unknown

8.5.4 Adverse event collection, recording and evaluation

The investigator should record all the adverse medical events occurring from signing of the ICF through 30 days after the end of the last dose (or until the subject starts new anti-tumor treatment, whichever is earlier).

The investigator will not actively collect any AE after the end of safety follow-up. In case of SAE, if the investigator thinks that there is a reasonable causal relationship between the SAE and the investigational drug, it should be reported to the sponsor according to the SAE reporting procedure.

Any AE that has not completely recovered or stabilized at the end of safety follow-up (regardless of the causal relationship) must be followed up until it is recovered (to the baseline level or completely recovered) or reaches clinical stability.

After signing of the ICF until the first dose, the clinical adverse event may be recorded in the CRF as medical history or concomitant disease, or shall be recorded as an AE if meeting one of the following circumstances: harm/damage caused by any clinical laboratory test manipulation; adverse event arising from the drug discontinuation related to protocol; or adverse event arising from the drug other than the investigational drug taken as a part of the treatment regimen.

All the AEs prior to the end of 30 days after the last dose must be recorded. The record contents shall include AE term, AE start date and end date (and time), severity, seriousness, relationship with the investigational drug in the opinion of the investigator, actions taken with the investigational drug for solving the AE and the outcome, etc.

• In case of subject death, the cause for death or the symptom should be reported the AE term, and “death” should be regarded as the AE outcome. If the cause for death is unknown, “death with unknown cause” should be reported as the AE term.

• The event obviously consistent with the tumor disease progression should not be recorded as adverse event, e.g. jaundice caused by tumor progression which leads to common bile duct compression, pain caused by tumor bone metastasis and intracranial hypertension caused by tumor brain metastases, unless it is progressed to be atypical or accelerated or caused by the investigational drug in the opinion of the investigator. However, death events caused by any reason within 30 days after the last dose should be reported to the sponsor according to the SAE procedure.

The investigator shall judge the causal relationship between the AE and the investigational drug as well as the study procedure and operation. See 8.5.3 for details.

For the SAE, the sponsor must independently evaluate the expectedness, severity and causal relationship to the investigational drug. The reference for expectedness evaluation is

the latest edition of investigator's brochure (IB).

8.5.5 Serious adverse event reporting

8.5.5.1 Responsibilities of the investigator

Reporting to the sponsor

For all the serious adverse events occurring during the observation in Section 8.5.4, the investigator must report them to the sponsor within 24h after informed. For the death events, the investigator shall provide the sponsor with other required information, e.g. necropsy report and final medical report.

Reporting to the ethics committee

The investigator shall read and sign the received clinical trial related safety information provided by the sponsor, and consider whether to make corresponding adjustment on the subject's treatment, and if necessary, the investigator shall communicate with the subject, and report the suspected unexpected serious adverse reactions provided by the sponsor to the ethics committee. For the death events, the investigator shall provide the ethics committee with other required information, e.g. necropsy report and final medical report.

8.5.5.2 Responsibilities of the sponsor

The sponsor shall report the suspected unexpected serious adverse reactions to the drug regulatory authorities and the health competent authority.

The reporting time limit is as follows:

- For fatal or life-threatening SUSAR, report them within 7 days after they are first notified (the day the applicant is first notified is the 0th day), and report and improve follow-up information within the following 8 days.
- For non-lethal or life-threatening SUSAR, report within 15 days.
- For follow-up reports, report within 15 days after the new information is obtained.

The sponsor shall rapidly report the suspected unexpected serious adverse reactions to all the investigators and clinical study sites participating in the clinical trial and the ethics committee.

8.5.6 Pregnancy

The investigator must report all the pregnancy events occurring during participation of female subjects in the study to the sponsor. The investigator shall carefully follow up the pregnancy outcome, and report any abnormal maternal or infant outcome.

If a male subject’s spouse becomes pregnant, the investigator shall obtain the information related to the pregnancy process and result as much as possible with the consent of the subject.

In case of pregnancy event during the study, the investigator shall communicate with the subject in a scientific and rigorous manner based on the medication information, and inform her/him of the possible effect and risk of investigational drug on the pregnant women and fetus. In case of pregnancy event in a female subject, the investigator shall immediately suspend the clinical trial of the subject, and discontinue the investigational drug.

The investigator shall complete the pregnancy report and submit the report to the sponsor (ethics committee of the hospital, if necessary) within 24h after confirming the pregnancy event in the subject (or the subject’s sex partner).

After informed of the pregnancy outcome, the investigator shall complete the pregnancy follow-up report and submit the report to the sponsor (ethics committee of the hospital, if necessary) within 24h after informed.

9 Statistical Considerations

9.1 Statistical Hypotheses

There are no official statistical hypotheses in the study, and the statistical analysis is for the purpose of exploration.

9.2 Sample Size Determination

Based on the defined cohort: Stage I: The dose escalation study is expected to enroll a minimum of 12 subjects. Stage II: According to the existing study results, the expected ORR of the combination therapy cohort is about 40%, and when the sample size is 90 subjects, the probability of observed ORR>35% calculated by normal approximation method is 83.4%; if the expected ORR is lower than 40%, the probability of observed ORR>35% shows a decreasing trend. Together with subjects in the dose escalation stage, the total number of subjects enrolled in all the cohorts is not more than 200. Safety and efficacy data are monitored periodically during the course of the study. Considering the benefits to the subjects, if a cohort shows clear evidence of treatment disadvantage, the cohort will be closed early to avoid more subjects receiving ineffective or low effective treatment; if a cohort shows clear evidence of treatment benefits, an increase in the number of subjects enrolled in that cohort may be considered.

A minimum of 12 subjects and a maximum of 200 subjects are expected to be enrolled in the whole study.

9.3 Analysis Population

Full analysis set (FAS): a collection of all subjects that have been successfully enrolled and used at least one dose of investigational drug.

Per-protocol set (PPS): a collection of subjects that meet the inclusion criteria, do not meet the exclusion criteria, and complete the treatment regime. That means a collection of all subjects who meet the study protocol, have good compliance, complete the treatment specified in the protocol, and have no major protocol deviation in the process will be analyzed.

Safety analysis set (Safety set, SS): All enrolled subjects who have used at least one dose of the investigational drug and have post-dosing safety records will be included in the safety analysis set.

DLT analysis Set (DLT set): The DLT set includes all subjects in the DLT assessment period (1 to 28 d), subjects who complete the DLT assessment or those who withdraw from the trial early due to adverse events during the DLT assessment period (subjects whose DLT is not evaluable due to intolerance to non-investigational drug dose will not be included in the DLT statistical sample). This analysis set will be used to analyze and summarize DLT events.

Pharmacokinetics concentration set (PKCS): Subjects who have received at least one dose of investigational drug and have at least one plasma concentration data of investigational drug during the trial.

Pharmacokinetics parameter set (PKPS): Subjects who have received at least one dose of investigational drug and have at least one effective calculation of PK parameter of investigational drug.

Immunogenicity analysis set: Subjects who have received at least one dose of investigational drug and have at least one sample of anti-drug antibodies.

Biomarker analysis set: Subjects who have received at least one dose of investigational drug and have at least one tumor tissue sample or at least one biomarker blood sample collected.

9.4 Statistical Analysis

9.4.1 General method

The statistical description of categorical variables is expressed as number of cases and percentage (%). The statistical description of continuous variables is described by number of cases, missing number, mean, standard deviation, median, lower quartile, upper quartile, minimum value, and maximum value. Unless otherwise specified, hypothesis testing will be performed using a two-sided test with a significant level of 0.05, and 95% CI will be used for the estimation of confidence intervals for parameters.

9.4.2 Subject disposition

The subject enrollment, medication, dropout or exclusion and dataset division are summarized, and presented by the schema of subject disposition.

The details of subject dropout and exclusion will be tabulated.

9.4.3 Baseline descriptive statistics

The baseline characteristics of each group are statistically described, including the demography, past medical history and treatment history.

The baseline characteristics, past medical history and treatment history of subjects will be tabulated.

9.4.4 Pharmacokinetic, immunogenicity and biomarker analysis

Pharmacokinetic analysis:

The PK concentration set and the plasma concentration (C) - time (t) data of each subject measured in the study will be used to plot the C-t curve. Meanwhile, the mean drug concentration at each time point and the standard deviation will be tabulated, and the mean plasma concentration curve is plotted (and the standard deviation is attached).

The non-compartmental model will be used to analyze the data of PK parameter set in the Stage I dose escalation study, and the pharmacokinetic parameters of each subject will be calculated, including C_{max}, AUC_{0-t}, AUC_{0-inf}, T_{max}, V_z, $t_{1/2}$, CL and $\%AUC_{ext}$ after the first dose; $C_{max,ss}$, $AUC_{0-\tau}$, AUC_{0-inf}, $T_{max,ss}$, V_{ss}, $t_{1/2}$, CL, C_{av}, DF, Range and $\%AUC_{ext}$ after repeated doses; accumulation index $R_{1ac}=C_{max,ss}/C_{max}$; accumulation index $R_{2ac}= AUC_{0-\tau}/AUC_{0-t}$. Meanwhile, the mean and standard deviation of each parameter will be calculated.

The drug concentration data in the Stage II dose escalation study and other JMT101 concentration data will be pooled for population pharmacokinetic analysis. Refer to the independent report for the analysis details and results.

Immunogenicity analysis:

The number of cases and incidence of positive ADA and NABs in each dose group will be pooled and listed separately.

Biomarker Analysis:

Pharmacodynamic biomarker: The cut-off value is determined according to the free DNA content of plasma, and the low and high expression groups are identified based on this value. The Kaplan-Meier is used to estimate the median PFS, median OS and 95% confidence interval of all subjects and dose groups, and survival curves will be plotted..

9.4.5 Safety analysis

Adverse events and adverse reactions:

Adverse events and adverse reactions during dosing will be analyzed to summarize the number of cases, frequency and percentage of adverse events, adverse reactions, trial discontinuation due to adverse events, death due to adverse events, serious adverse events, and DLT events in each group of subjects; group comparisons of incidence will be performed when necessary.

The adverse events and adverse reactions are coded by the system organ class (SOC) and the preferred term (PT) according to the MedDRA, and the number of cases, number of occurrences and percentage of adverse events and adverse reactions will be analyzed by classification statistically according to the SOC/PT:

The number of cases, frequency and percentage of adverse events and adverse reactions will be counted by SOC/PT and severity. A class of adverse events in a subject is counted only once under the highest severity within the same term (SOC or PT).

All adverse events (including those occurring during non-dosing periods), adverse reactions, serious adverse events, study discontinuation due to adverse events, death due to adverse events, adverse events of special interest, and DLT events will be tabulated separately.

Laboratory test:

The statistical time points for each laboratory test item include baseline, each post-baseline visit and the last visit. The measured values at each time point, the minimum and maximum values of post-baseline measurements and observed values at the last visit, as well as their change values from baseline will be counted by dose group and total, respectively.

The normal or abnormal changes of each parameter before and after administration are compared using a cross tabulation of clinical judgment.

A detailed list of each laboratory test parameter is presented by group.

Vital sign:

The statistical time points for vital sign examination items include baseline, each post-baseline visit, and early withdrawal visit. The descriptive statistics results at baseline, post-baseline visit and at the end of the study are counted by group and total. The normal or abnormal changes of each parameter before and after administration are compared using a cross tabulation of clinical judgment (if applicable).

Physical examination and ECG:

Physical examination items: general condition, skin and mucosa, lymph node, head and neck, chest, abdomen, spine, muscles and skeletons, nervous system and other parts. The normal or abnormal changes of each dose group before and after administration are compared using a cross tabulation of clinical judgment.

ECG examination items include: heart rate, PR, QRS, QTc. The minimum and maximum and last visit observations and their change form baseline will be statistically analyzed by dose group and total for each visit time point at baseline, post-baseline measurements. The normal or abnormal changes of each dose group before and after administration are compared using a cross tabulation of clinical judgment.

A detailed list of physical examination and ECG is presented by group.

9.4.6 Efficacy analysis

Objective response rate (ORR: complete response (CR) rate + partial response (PR) rate, assessed by IRC and investigators respectively), duration of response (DOR), disease control rate (DCR: complete response (CR) rate + partial response (PR) rate + stable disease (SD) rate), progression-free survival (PFS), and overall survival (OS) will be assessed according to RECIST v1.1 criteria and relevant evaluation results.

The number of cases, percentage and 95% CI of ORR and DCR are calculated for each dose group versus all subjects.

The Kaplan-Meier is used to estimate the median DOR, median PFS, median OS and 95% CI of all subjects and dose groups, and survival curve will be plotted.

9.4.7 Planned interim analysis

The detailed analysis plan will be described in the statistical analysis plan (SAP).

9.4.8 Sub-group analyses

The detailed analysis plan will be described in the statistical analysis plan (SAP).

9.4.9 Exploratory analyses

N/A

10 Supporting Documentation and Operation Considerations

10.1 Regulatory, Ethical, and Study Oversight Considerations

10.1.1 Informed consent process

10.1.1.1 Informed consent form and other documents provided to the subjects

The study intervention, study procedure and risk informed consent form should be explained to the subjects in detail, and written informed consent document should be obtained prior to the study intervention/medication.

10.1.1.2 Informed consent process and document

Informed consent is a step started before an individual agrees to participate in the study and maintained in the entire participation in the study. The informed consent form will be approved by the institutional review board (IRB), and the subjects will be requested to read and check this document. The investigator will explain the study to the subjects, and answer any question from the subjects. The investigator will orally explain the study objective, procedure and potential risk and the right as a subject to the subjects in a manner suitable for understanding. The subjects should have sufficient time to carefully read the informed consent form and ask questions before signing the written informed consent form. The subjects should have opportunities to discuss the study or consider by themselves with family members or agents before consent to participation in the study. The subjects will sign the informed consent form before any study-specific procedure. The subjects must be informed that participation in the study is voluntary, and they can withdraw from the study at any time without getting damaged. The investigator will provide the subjects with the copy of informed consent form for safekeeping. The informed consent should be completed before the subjects receive any study-specific procedure, the informed consent process (including date) should be recorded in the source document, and the signed informed consent forms should be kept properly. The investigator must particularly inform the subjects that "if they reject to participate in the study, their medical service quality will not be adversely affected", so as to ensure the rights and benefits of the subjects.

10.1.2 Study suspension and termination

If there is a sufficient reasonable reason, the study might be temporarily suspended or terminated early. The party suspending or terminating the study should provide written notice to the subjects, the investigator and the regulatory agency, and record the reason for study suspension or termination. If the study is temporarily suspended or terminated early, the principal investigator should notify the subjects, the ethics committee and the sponsor, and provide the reason for study suspension or termination. The investigator will contact the subjects and notify the change in the visit schedule (if applicable).

The circumstances that possibly require study termination or temporary suspension include but are not limited to:

- Identified unexpected, major or unacceptable risk to the subjects
- Established efficacy that supports study termination
- Poor compliance with the protocol requirements

- Incomplete and/or unevaluable data
- Success in reaching the primary endpoint
- Determined ineffective study intervention

The study can be continued only when the safety, protocol compliance and data quality related problems are solved, and the requirements for the sponsor, the ethics committee and/or the regulator are satisfied.

10.1.3 Confidentiality and privacy

The confidentiality and privacy of subjects should be strictly kept confidential by the participating investigator, the staff and the sponsor as well as the intervention measures. In addition to the clinical information related to the subjects, such confidentiality should also be extended to cover the biological sample testing and the gene testing. Therefore, the protocol, document, data and all the other information generated in the study will be strictly kept confidential. Without the written permission of the sponsor, any related study information or data should not be disclosed to any unauthorized third party.

All the study activities will be conducted in a private environment.

The study monitor, the sponsor's other authorized representatives, the IRB's representatives, the regulator's representatives or the representatives of pharmaceutical companies providing the products may check all the documents and records to be kept by the investigator, including but not limited to the medical records (in office, clinic or hospital) and pharmacy records of subjects in the study. The clinical study site shall allow access to these records.

The contact information of subjects will be safely kept in each clinical study site for internal use during the study. At the end of the study, all the records will be still kept at a safety location for the time longer than that required by the IRB, the institution or the sponsor.

The study data of subjects for statistical analysis and scientific research report will be sent to or saved in the data management institution. This does not include the contact information or identification information of the subjects. On the contrary, individual subject and his/her study data will be identified by the unique study authentication code. The study data and the study management system used will be safety protected with password. At the end of the study, all the study databases will be de-identified, and saved in the data management institution.

10.1.4 Future use of stored specimens and data

The data collected in the study will be analyzed and saved in the designated data management institution. After completion of the study, the archived data excluding the personal information will be transferred to and saved in the EDC for use of other investigators (including the investigators of other studies). The permission to transfer data to the EDC will be regarded as one of the informed consent contents.

With the consent of subjects and the approval of the local institutional review board (IRB), the biological samples without personal information will be saved in the third party designated by the sponsor (or destroyed), identical with the shared data stored in the EDC. Due to increased risk of related complications and other symptoms for the patients with advanced solid tumors, these samples might be used for study on the pathogenesis of advanced solid tumors or for improvement of treatment methods. While keeping the personal information of subjects blind, the code associated information between the biological samples and the phenotype data of subjects shall also be provided for the EDC.

During the study, the subjects may select to withdraw the informed consent of allowing the stored biological samples to be used for other studies. However, after completion of the

study, the informed consent on the stored biological samples might not be withdrawn.

After completion of the study, the access to the study data and/or samples will be provided via the EDC.

10.1.5 Key role and study governance

**Principal investigator**	**Medical monitor**
Professor Zhang Li, Chief Physician	Hu Rong, Medical Manager
Sun Yat-sen University Cancer Center	Shanghai JMT-Bio Inc.
No. 651, Dongfeng East Road, Yuexiu District, Guangzhou, Guangdong	SOHO Donghai Plaza, No. 299, Tongren Road, Jing’an District, Shanghai
020-87343458	021-60677906
zhangli@sysucc.org.cn	hurong@mail.ecspc.com

10.1.6 Safety surveillance

Not applicable.

10.1.7 Quality control and quality assurance

The clinical study parties (e.g. the sponsor and the study site) should take appropriate quality control measures to ensure that the clinical study conforms to the Declaration of Helsinki, the GCP and the corresponding laws and regulations as well as the SOPs.

Quality control

The monitoring of the clinical study will be in the responsibility of the monitor of the sponsor, Shanghai JMT-Bio Inc. The sponsor should ensure that appropriate monitor is conducted before, during and after the clinical study. The sponsor should make the corresponding monitor plan according to the situation of the clinical study, and the monitor should supervise the informed consent documents of subjects, drugs, equipment, original documents of subjects, investigator’s folders and all the other study documents according to the monitor plan.

The data should be verified to confirm the accuracy by comparing the data in the eCRF with the appropriate source documents. All the inconsistencies should be amended by the investigator, or the personnel designated by the investigator. The monitor activities should be recorded, including but not limited to the monitor visit documents and the records of communication with the investigator via letter, email, fax or telephone.

The investigator should allow the monitor to directly check the related documents and communicate with the monitor after monitoring to understand and discuss the monitor results and the existing problems.

Quality assurance

Except the routine monitoring, Shanghai JMT-Bio Inc. also has the GCP quality assurance department. The department will audit the clinical study activities according to the audit procedures of the company to evaluate whether the execution of the study meets the GCP, the protocol and the corresponding laws and regulations.

The investigator should agree and allow the auditor to directly check the original documents, the eCRF and other related documents about subjects' participation in the study, and shall share sufficient time to participate in the audit and discover and discuss the problems.

If the regulator informs the investigator of the audit, the investigator shall immediately contact with Shanghai JMT-Bio Inc.

10.1.8 Clinical study monitor

The clinical study site should be monitored to guarantee the rights and benefits of

subjects, to ensure the study data accuracy, completeness and reliability, and to ensure the clinical study complies with the latest approved protocol or protocol amendment, the Good Clinical Practice of International Conference on Harmonization (ICH GCP) and the applicable laws and regulations.

- The monitor of the clinical study will be in the responsibility of Shanghai JMT-Bio Inc. The monitor (who must be experienced and qualified) will contact and visit the regulator regularly to ensure that the clinical study complies with the protocol, the Good Clinical Practice (GCP), the SOPs and the related regulations, to ensure that the original records related to the clinical study are reserved, and to ensure that the eCRF contents are complete, accurate and verifiable compared to the original data.
- The investigator will allow the monitor to directly access all the original data, including electronic medical records and/or documents helpful for data validation. The investigator will cooperate with the monitor in ensuring that all the possible deviations can be solved. The investigator is expected to meet with the monitor during the visits. The first subject will receive the monitor visit soon after being assigned to receive the investigational drug at the study site.
- 100% source data validation (SDV) will be conducted in the clinical study.

10.1.9 Data processing and record retention

Data collection and management responsibilities

The staff of clinical study will be responsible for data collection. The investigator should supervise the data collection and ensure the accuracy, completeness, readability and timeliness of reported data.

All the source documents should be kept clean and clear to ensure that the data can be identified accurately.

The permanent copies of study visit records will be regarded as source documents to record the data of enrolled subjects. The data recorded in the electronic case report form (eCRF) shall be from the source documents, and should be consistent with the source data.

The clinical data, including adverse events (AEs), concomitant medications, expected adverse drug reaction data and laboratory data, will be entered in the EDC. The system will be provided by a qualified EDC supplier, consistent with the provisions in Section 11 of 21 CFR. The data system contains the password protection and internal quality inspection functions. For example, it can identify the automatic domain of inconsistent, incomplete or inaccurate data. The clinical data will be directly entered according to the source documents.

Retention of study records

The investigator should retain the important documents related to the clinical study properly (including the investigator's folder) according to the related regulations.

All the clinical study data must be retained for at least 5 years after the end of the study. It is not allowed to destroy any data without the prior written consent of the sponsor. The sponsor is responsible for informing the investigator of the terminated retention date of the above documents.

10.1.10 Protocol deviations

Protocol deviations refers to any non-compliance with the clinical study protocol, the GCP and ICH-GCP or the Manual of Operation (MOP). Non-compliance might be from the subjects, the investigator or the staff of clinical study site. Collective measures should be taken and completed in a timely manner for such deviations.

In case of protocol deviations, the investigator must notify the monitor to review and discuss the related significance of deviations. Any deviations must be recorded as the answer

to the query in the eCRF and/or in the protocol deviations report. The protocol deviations report will be kept by Shanghai JMT-Bio Inc. The protocol deviations report and the supporting documents must be retained in the investigator's folder.

10.1.11 Study publication and data sharing policy

The intellectual property rights of the data and results derived from the clinical study as well as all the data and results shall belong to the sponsor. The investigator may use the data derived from the clinical study for scientific purpose, but must discuss with the sponsor before publication, and the contents to be published must be subject to the prior written consent of the sponsor.

The sponsor acknowledges that the investigator has the right to publish the results at the end of the clinical study. However, the investigator must send the manuscript to the sponsor before submission. The sponsor will review whether the manuscript is accurate (to avoid inconsistency with the information submitted to the regulator), prevent the confidential or patent information from being disclosed, and supplement the related information as appropriate. The manuscript will be approved in a timely manner, and will not be rejected for publication without reason. If the sponsor disagrees with the investigator, the contents to be published will be discussed to find a solution satisfactory to all parties.

For a multi-center study, the first publication must be based on the data of all sites, and must be analyzed by the biostatistician designated or recognized by the sponsor according to the protocol, rather than by the investigator alone. The investigator participating in the multi-center study shall not publish the data collected from one or several sites before the first publication of the data from all the sites, unless otherwise officially agreed by all the other investigators and the sponsor.

The manuscript author will be determined upon negotiation of all parties. If it is submission of summary data, the author may include the members participating in each study site and the staff of the sponsor.

The publication of study results will be detailed in the clinical study agreement.

10.2 Historical Record of Protocol Modifications

**Version No.**	**Version date**
v1.0	December 27, 2019
v2.0	June 23, 2020
v3.0	December 10, 2020

11 References

[1] Sako Y,minoghchi S, Yanagida T. Single-molecule imaging of EGFR signalling on the surface of living cells. Nature cell biology. 2000;2(3):168-72. doi;10.1038/35004044

[2] Lewis TS, Shapiro PS, Ahn NG. Signal transduction throughmAP kinase cascades. Advances in cancer research. 1998;74:49-139. doi:10.1016/S0065-230X(08)60765-4

[3] Zhao L, He LR, Xim, et al. Nimotuzumab promotes radiosensitivity of EGFR-overexpression esophageal squamous cell carcinoma cells by upregulating IGFBP-3. Journal of translational medicine. 2012;10:249. doi:10.1186/1479-5876-10-249

[4] Zhao L, Li QQ, Zhang R, et al. The overexpression of IGFBP-3 is involved in the chemosensitivity of esophageal squamous cell carcinoma cells to nimotuzumab combined with cisplatin. Tumour biology: the journal of the International Society for Oncodevelopmental Biology and medicine. 2012;33(4):1115-23. doi: 10.1007/s13277-012-0352-0

[5] Gao Yun, Chen Jiachang, Zhu Zhenyu, etc. Research Progress of EGFR Gene Mutation and Test Method [J]. Journal of Molecular Diagnosis and Therapy, 2019, 24(5): 454-462. doi: 10.3969/j.issn.1674-6929.2011.01.014

[6] Doody JF, Wang Y, Patel SN, et al. Inhibitory activity of cetuximab on epidermal growth factor receptor mutations in non small cell lung cancers. molecular cancer therapeutics. 2007;6(10):2642-51. doi: 10.1158/1535-7163.MCT-06-0506

[7] Matar P, Rojo F, Cassia R, et al. Combined epidermal growth factor receptor targeting with the tyrosine kinase inhibitor gefitinib (ZD1839) and the monoclonal antibody cetuximab (IMC-C225): superiority over single-agent receptor targeting. Clinical cancer research: an official journal of the American Association for Cancer Research. 2004;10(19):6487-501. doi: 10.1158/1078-0432.ccr-04-0870

[8] Fukuoka S, Kojima T, Koga Y, et al. Preclinical efficacy of Sym004, novel anti-EGFR antibody mixture, in esophageal squamous cell carcinoma cell lines. Oncotarget. 2017;8(7):11020-9.

[9] Rubin Grandis J, Chakraborty A, melhemmF, Zeng Q, Tweardy DJ. Inhibition of epidermal growth factor receptor gene expression and function decreases proliferation of head and neck squamous carcinoma but not normal mucosal epithelial cells. Oncogene. 1997;15(4):409-16. doi: 10.1038/sj.onc.1201188

[10] Salomon DS, Brandt R, Ciardiello F, Normanno N. Epidermal growth factor-related peptides and their receptors in humanmalignancies. Critical reviews in oncology/hematology. 1995;19(3):183-232. doi: 10.1016/1040-8428(94)00144-I

[11] Di Fiore PP, Pierce JH, Fleming TP, et al. Overexpression of the human EGF receptor confers an EGF-dependent transformed phenotype to NIH 3T3 cells. Cell. 1987;51(6):1063-70. doi: 10.1016/0092-8674(87)90592-7

[12] Arteaga CL. The epidermal growth factor receptor: from mutant oncogene in nonhuman cancers to therapeutic target in human neoplasia. Journal of clinical oncology: official journal of the American Society of Clinical Oncology. 2001;19(18 Suppl):32s-40s. doi:10.1002/ijc.1442

[13] Bronte G, Terrasim, Rizzo S, et al. EGFR genomic alterations in cancer: prognostic and predictive values. Frontiers in bioscience (Elite edition). 2011;3:879-87. doi: 10.2741/e296

[14] Gullick WJ. Prevalence of aberrant expression of the epidermal growth factor receptor in human cancers. British medical bulletin. 1991;47(1):87-98. doi:

10.1093/oxfordjournals.bmb.a072464
[15] Herbst RS, Heymach JV, Lippman SM. Lung cancer. N E J med, 2008, 359(13): 1367-1380. doi: 10.1056/NEJMra0802714.
[16] Wu YL, Zhong WZ, Li LY, et al. Epidermal growth factor receptor mutations and their correlation with gefitinib therapy in patients with non-small cell lung cancer: ameta-analysis based on updated individual patient data from six medical centers in mainland China. J Thorac Oncol, 2007, 2(5): 430-9.
[17] Shi Y, Au JS, Thongprasert SA, et al. Prospective molecular epidemiology study of EGFR mutations in Asian patients with advanced non small cell lung cancer of adenocarcinoma histology (PIONEER). J Thorac Oncol, 2014, 9(2): 154-62. doi: 0.1097/01.JTO.0000268677.87496.4c.
[18] Gou LY, Wu YL. Prevalence of driver mutations in non-small-cell lung cancers in the People’s Republic of China. Lung Cancer Target Therapy, 2014, 5: 1-9. doi: 10.2147/LCTT.S40817
[19] Yang JCH, Sequist LV, Greater SL, et al. Clinical activity of afatinib in patients with advanced non-small-cell lung cancer harbouring uncommon EGFR mutations: a combined post-hoc analysis of LUX-Lung 2, LUX-Lung 3, and LUX-Lung 6. Lancet Oncol, 2015, 16(7): 830-8. doi: 10.1016/S1470-2045(15)00026-1
[20] Su KY, Chen HY, Li KC, et al. Pretreatment epidermal growth factor receptor (EGFR) T790M mutation predicts shorter EGFR tyrosine kinase inhibitor response duration in patients with non small cell lung cancer. J Clin Oncol, 2012, 30(4): 433-40. doi: 10.1200/JCO.2011.38.3224
[21] ArcilamE, Nafa K, Chaft JE, et al. EGFR exon 20 insertion mutations in lung adenocarcinomas: prevalence, molecular heterogeneity, and Clinicopathologic characteristics. Mol Cancer Ther. 2013;12(2):220-9. doi: 10.1158/1535-7163.MCT-12-0620
[22] Riess JW, Gandara DR, Frampton GM, et al. Diverse EGFR exon 20 insertions and co-occurring molecular alterations identified by comprehensive genomic profiling of non-small cell lung Cancer. J Thorac Oncol. 2018;13(10):1560-8. doi: 10.1016/j.jtho.2018.06.019
[23] Oxnard GR, Lo PC, Nishinom, et al. Natural history and molecular characteristics of lung cancers harboring EGFR exon 20 insertions. J Thorac Oncol. 2013;8(2):179-84. doi: 10.1097/JTO.0b013e3182779d18
[24] Fang W, Huang Y, Hong S,et al. EGFR exon 20 insertion mutations and response to osimertinib in non-small-cell lung cancer BMC Cancer. 2019;19(1):595. doi: 10.1186/s12885-019-5820-0
[25] Tu HY, Ke EE, Yang JJ, et al. A comprehensive review of uncommon EGFR mutations in patients with non-small cell lung cancer. Lung Cancer. 2017;114:96-102. doi: 10.1016/j.lungcan.2017.11.005
[26] Shen YC, Tseng GC, Tu CY, et al. Comparing the effects of afatinib with gefitinib or Erlotinib in patients with advanced-stage lung adenocarcinoma harboring non-classical epidermal growth factor receptor mutations. Lung Cancer. 2017;110:56-62. doi: 10.1016/j.lungcan.2017.06.007
[27] Naidoo J, Sima CS, Rodriguez K, et al. Cancer. Epidermal growth factor receptor exon 20 insertions in advanced lung adenocarcinomas: Clinical outcomes and response to erlotinib. Cancer. 2015 September 15; 121(18): 3212-20. doi: 10.1002/cncr.29493
[28] B van Veggel. 1450P - Osimertinib treatment for patients with EGFR exon 20 insertion

positive non-small cell lung cancer. 2018 ESMO.
[29] Taemin Kim. 1529P - Phase II study of osimertinib in NSCLC patients with EGFR exon 20 insertion mutation: A multicenter trial of the Korean Cancer Study Group (LU17-19). 2019 ESMO.
[30] Fossella F, Periera JR, von Pawel J, et al. Randomized, multinational, phase III study of docetaxel plus platinum combinations versus vinorelbine plus cisplatin for advanced non-small cell lung cancer: the TAX 326 study group. J Clin Oncol 2003;21(16):3016-24. doi: 10.1200/JCO.2003.12.046
[31] Scagliotti GV, Parikh P, von Pawel J, et al. Phase III study comparing cisplatin plus gemcitabine with cisplatin plus pemetrexed in chemotherapy-naive patients with advanced-stage NSCLC. J Clin Oncol 2008;26:3543-51. doi: 10.1200/JCO.2007.15.037
[32] Danson S, middletonm R, O’Byrne KJ, et al. Phase III trial of gemcitabine and carboplatin versus mitomycin, ifosfamide, and cisplatin ormitomycin, vinblastine, and ciplatin in patients with advanced non-small cell lung carcinoma. Cancer 2003;98:542-53. doi: 10.1002/cncr.11535
[33] Ohe Y, Ohashi Y, Kubota K, et al. Randomized phase III study of cisplatin plus irinotecan versus carboplatin plus paclitaxel, cisplatin plus gemcitabine, and cisplatin plus vinorelbine for advanced non-small-cell lung cancer: Four-Arm Cooperative Study in Japan. Ann Oncol 2007;18:317-23. doi: 10.1093/annonc/mdl377
[34] Vermorken JB, mesia R, Rivera F, et al. Platinum-based chemotherapy plus cetuximab in head and neck cancer. The New England journal of medicine. 2008;359(11):1116-27. doi: 10.1056/NEJMoa0802656
[35] Jonker DJ, O'Callaghan CJ, Karapetis CS, et al. Cetuximab for the treatment of colorectal cancer. The New England journal of medicine. 2007;357(20):2040-8. doi: 10.1056/NEJMoa071834
[36] Van Cutsem E, Peetersm, Siena S, et al. Open-label phase III trial of panitumumab plus best supportive care compared with best supportive care alone in patients with chemotherapy-refractory metastatic colorectal cancer. Journal of clinical oncology: official journal of the American Society of Clinical Oncology. 2007;25(13):1658-64. doi: 10.1200/JCO.2006.08.1620
[37] Lable for ERBITUX® (cetuximab) Injection for intravenous use, SUPPL-265, Action: 10/11/2016.
[38] Lable for VECTIBIX® (panitumumab) Injection for intravenous use, SUPPL-207, Action: 06/29/2017.
[39] Lable for PORTRAZZA (necitumumab) injection, ORIG-1, Action Date: 11/24/2015.
[40] Package Insert of Nimotuzumab lnjection (TaiXinSheng). Approval No.: S20080001, Biotech Pharma Co., Ltd
[41] NCCN Clinical Practice Guidelines in Oncology: Colon cancer. Version 2. 2018. https://www.nccn.org/professionals/physician_gls/default.aspx#site.
[42] Package Insert of Cetuximab Injection (Erbitux). Approval No.: S20171039, Merck KGaA
[43] Hasegawa H et al. Efficacy of afatinib or osimertinib plus cetuximab combination therapy for non-small-cell lung cancer with EGFR exon 20 insertion mutations. Lung Cancer. 2019;127:146-52. doi: 10.1016/j.lungcan.2018.11.039
[44] Riess J, Gandara D R, Frampton G, et al. OA10.01 Comprehensive Genomic Profiling and PDX modeling of EGFR Exon 20 Insertions: Evidence for Osimertinib Based Dual EGFR Blockade[C]// 2017:S279-80. doi: 10.1016/j.jtho.2016.11.282

[45] van Veggel B, de Langen AJ, Hashemi SMS, et al. Afatinib and Cetuximab in Four Patients With EGFR Exon 20 Insertion-Positive Advanced NSCLC. Thorac Oncol. 2018;13(8):1222-6. doi: 10.1016/j.jtho.2018.04.012
[46] Fang Wenfeng, Huang Yihua, Gan Jiadi, Hong Shaodong, Zhang Li. A Patient with EGFR Exon 20 Insertion-Mutant Non-Small Cell Lung Cancer Responded to Osimertinib plus Cetuximab Combination Therapy. J Thorac Oncol. 2019;14(9):e201-2. doi：10.1016/j.jtho.2019.04.013
[47] Hofheinz RD, Deplanque G, Komatsu Y, et al. Recommendations for the Prophylactic management of Skin Reactions Induced by Epidermal Growth Factor Receptor Inhibitors in Patients With Solid Tumors. The oncologist. 2016;21(12):1483-91. doi: 10.1634/theoncologist.2016-0051

Appendix

Appendix - ECOG Performance Status

Grade	Performance status
0	Fully active, able to carry on all pre-disease performance without restriction.
1	Restricted in physically strenuous activity but ambulatory and able to carry out work of a light or sedentary nature, e.g., light housework, office work
2	Ambulatory and capable of all selfcare but unable to carry out any work activities; Up and about more than 50% of waking hours.
3	Capable of only limited selfcare, confined to bed or chair more than 50% of waking hours.
4	Completely disabled; Cannot carry out any selfcare; Totally confined to bed or chair.
5	Dead

Appendix II Heart Function Grade of New York Heart Association (NYHA)

Grade	Patient symptom
Grade I	No limitation of physical activity. Ordinary physical activity does not cause undue fatigue, palpitation, dyspnea (shortness of breath).
Grade II	Slight limitation of physical activity. Comfortable at rest. Ordinary physical activity results in fatigue, palpitation, dyspnea.
Grade III	Marked limitation of physical activity. Comfortable at rest. Less than ordinary activity causes fatigue, palpitation, or dyspnea.
Grade IV	Unable to carry on any physical activity without discomfort. Symptoms of heart failure at rest. If any physical activity is undertaken, discomfort increases.

Appendix III Response Evaluation Criteria in Solid Tumors (RECIST 1.1) Excerpts

1. Background

1.1 History of RECIST criteria

Assessment of the change in tumour burden is an important feature of the clinical evaluation of cancer therapeutics. Both tumour shrinkage (objective response) and time to the development of disease progression are important endpoints in cancer clinical trials. The use of tumour regression as the endpoint for phase II trials screening new agents for evidence of anti-tumour effect is supported by years of evidence suggesting that, for many solid tumours, agents which produce tumour shrinkage in a proportion of patients have a reasonable (albeit imperfect) chance of subsequently demonstrating an improvement in overall survival or other

time to event measures in randomised phase III studies. At the current time objective response carries with it a body of evidence greater than for any other biomarker supporting its utility as a measure of promising treatment effect in phase II screening trials. Furthermore, at both the phase II and phase III stage of drug development, clinical trials in advanced disease settings are increasingly utilising time to progression (or progression-free survival) as an endpoint upon which efficacy conclusions are drawn, which is also based on anatomical

measurement of tumour size.

However, both of these tumour endpoints, objective response and time to disease progression, are useful only if based on widely accepted and readily applied standard criteria

based on anatomical tumour burden. In 1981 the World Health Organisation (WHO) first published tumour response criteria, mainly for use in trials where tumour response was the primary endpoint. The WHO criteria introduced the concept of an overall assessment of tumour burden by summing the products of bidimensional lesion measurements and determined response to therapy by evaluation of change from baseline while on treatment. However, in the decades that followed their publication, cooperative groups and pharmaceutical companies that used the WHO criteria often ‘modified’ them to accommodate new technologies or to address areas that were unclear in the original document. This led to confusion in interpretation of trial results and in fact, the application of varying response criteria was shown to lead to very different conclusions about the efficacy of the same regimen. In response to these problems, an International Working Party was formed in the mid 1990s to standardize and simplify response criteria. New criteria, known as RECIST (Response Evaluation Criteria in Solid Tumours), were published in 2000. Key features of the original RECIST include definitions of minimum size of measurable lesions, instructions on how many lesions to follow (up to 10; a maximum five per organ site), and the use of unidimensional, rather than bidimensional, measures for overall evaluation of tumour burden. These criteria have subsequently been widely adopted by academic institutions, cooperative groups, and industry for trials where the primary endpoints are objective response or progression. In addition, regulatory authorities accept RECIST as an appropriate guideline for these assessments.

2. Purpose of this guideline

This guideline describes a standard approach to solid tumor measurement and definitions for objective assessment of change in tumor size for use in adult and paediatric cancer clinical trials. It is expected these criteria will be useful in all trials where objective response is the primary study endpoint, as well as in trials where assessment of stable disease, tumor progression or time to progression analyses are undertaken, since all of these outcome measures are based on an assessment of anatomical tumor burden and its change on study. There are no assumptions in this paper about the proportion of patients meeting the criteria for any of these endpoints which will signal that an agent or treatment regimen is active: those definitions are dependent on type of cancer in which a trial is being undertaken and the specific agent(s) under study. Protocols must include appropriate statistical sections which define the efficacy parameters upon which the trial sample size and decision criteria are based. In addition to providing definitions and criteria for assessment of tumour response, this guideline also makes recommendations regarding standard reporting of the results of trials that utilize tumor response as an endpoint.

While these guidelines may be applied in malignant brain tumor studies, there are also separate criteria published for response assessment in that setting. This guideline is not intended for use for studies of malignant lymphoma since international guidelines for response assessment in lymphoma are published separately.

Finally, many oncologists in their daily clinical practice follow their patients’ malignant disease by means of repeated imaging studies and make decisions about continued therapy on the basis of both objective and symptomatic criteria. It is not intended that these RECIST guidelines play a role in that decision making, except if determined appropriate by the

treating oncologist.

3. Measurability of tumor at baseline

3.1 Definitions

At baseline, tumor lesions/lymph nodes will be categorized measurable or non-measurable as follows:

3.1.1 Measurable (lesion)

Tumor lesions: Must be accurately measured in at least one dimension (longest diameter in the plane of measurement is to be recorded) with a minimum size of:

- 10 mm by CT scan (CT scan slice thickness no greater than 5 mm).
- 10 mm caliper measurement by clinical exam (lesions which cannot be accurately measured with calipers should be recorded as non-measurable).
- 20 mm by chest X-ray.

Malignant lymph nodes: To be considered pathologically enlarged and measurable, a lymph node must be P15mm in short axis when assessed by CT scan (CT scan slice thickness

recommended to be no greater than 5 mm). At baseline and in follow-up, only the short axis will be measured and followed.

3.1.2 Non-measurable (lesion)

All other lesions, including small lesions (longest diameter <10 mm or pathological lymph nodes with ≥10 to <15 mm short axis) as well as truly non-measurable lesions. Lesions

considered truly non-measurable include: leptomeningeal disease, ascites, pleural or pericardial effusion, inflammatory breast disease, lymphangitic involvement of skin or lung, abdominal masses/abdominal organomegaly identified by physical exam that is not measurable by reproducible imaging techniques.

3.1.3 Special considerations regarding lesion measurability

Bone lesions, cystic lesions, and lesions previously treated with local therapy require particular comment:

Bone lesions:

- Bone scan, PET scan or plain films are not considered adequate imaging techniques to measure bone lesions. However, these techniques can be used to confirm the presence or disappearance of bone lesions.
- Lytic bone lesions or mixed lytic-blastic lesions, with identifiable soft tissue components, that can be evaluated by cross sectional imaging techniques such as CT or MRI can be considered as measurable lesions if the soft tissue component meets the definition of measurability described above.
- Blastic bone lesions are non-measurable.

Cystic lesions:

- Lesions that meet the criteria for radiographically defined simple cysts should not be considered as malignant lesions (neither measurable nor non-measurable) since they are, by definition, simple cysts.
- ‘Cystic lesions’ thought to represent cystic metastases can be considered as measurable lesions, if they meet the definition of measurability described above. However, if noncystic lesions are present in the same patient, these are preferred for selection as target lesions.

Lesions with prior local treatment:

- Tumor lesions situated in a previously irradiated area, or in an area subjected to other

loco-regional therapy, are usually not considered measurable unless there has been demonstrated progression in the lesion. Study protocols should detail the conditions under which such lesions would be considered measurable.

3.2 Specifications by methods of measurements

3.2.1 Measurement of lesions

All measurements should be recorded in metric notation, using calipers if clinically assessed. All baseline evaluations should be performed as close as possible to the treatment start and never more than 4 weeks before the beginning of the treatment.

3.2.2 Method of assessment

The same method of assessment and the same technique should be used to characterise each identified and reported lesion at baseline and during follow-up. Imaging based evaluation should always be done rather than clinical examination unless the lesion(s) being followed cannot be imaged but are assessable by clinical exam.

Clinical lesions: Clinical lesions will only be considered measurable when they are superficial and P10mm diameter as assessed using calipers (e.g. skin nodules). For the case of skin lesions, documentation by colour photography including a ruler to estimate the size of the lesion is suggested. As noted above, when lesions can be evaluated by both clinical exam

and imaging, imaging evaluation should be undertaken since it is more objective and may also be reviewed at the end of the study.

Chest X-ray: Chest CT is preferred over chest X-ray, particularly when progression is an important endpoint, since CT is more sensitive than X-ray, particularly in identifying new lesions. However, lesions on chest X-ray may be considered measurable if they are clearly defined and surrounded by aerated lung.

CT, MRI: CT is the best currently available and reproducible method to measure lesions selected for response assessment. This guideline has defined measurability of lesions on CT scan based on the assumption that CT slice thickness is 5 mm or less. When CT scans have slice thickness greater than 5 mm, the minimum size for a measurable lesion should be twice the slice thickness. MRI is also acceptable in certain situations (e.g. for body scans).

Ultrasound: Ultrasound is not useful in assessment of lesion size and should not be used as a method of measurement. Ultrasound examinations cannot be reproduced in their entirety for independent review at a later date and, because they are operator dependent, it cannot be guaranteed that the same technique and measurements will be taken from one assessment to the next. If new lesions are identified by ultrasound in the course of the study, confirmation by CT or MRI is advised. If there is concern about radiation exposure at CT, MRI may be used instead of CT in selected instances.

Endoscopy, laparoscopy: The utilization of these techniques for objective tumour evaluation is not advised. However, they can be useful to confirm complete pathological response when biopsies are obtained or to determine relapse in trials where recurrence following complete response or surgical resection is an endpoint.

Tumor markers: Tumor markers alone cannot be used to assess objective tumor response. If markers are initially above the upper normal limit, however, they must normalise for a patient to be considered in complete response. Because tumor markers are disease specific, instructions for their measurement should be incorporated into protocols on a disease specific basis. Specific guidelines for both CA-125 response (in recurrent ovarian cancer) and PSA response (in recurrent prostate cancer), have been published. In addition, the Gynecologic Cancer Intergroup has developed CA125 progression criteria which are to be integrated with objective tumor assessment for use in first-line trials in ovarian cancer.

Cytology, histology: These techniques can be used to differentiate between PR and CR in rare cases if required by protocol (for example, residual lesions in tumor types such as germ cell tumors, where known residual benign tumors can remain). When effusions are known to be a potential adverse effect of treatment (e.g. with certain taxane compounds or angiogenesis inhibitors), the cytological confirmation of the neoplastic origin of any effusion that appears or worsens during treatment can be considered if the measurable tumor has met criteria for response or stable disease in order to differentiate between response (or stable disease) and progressive disease.

4. Tumor response evaluation

4.1 Assessment of overall tumor burden and measurable lesion

To assess objective response or future progression, it is necessary to estimate the overall tumor burden at baseline and use this as a comparator for subsequent measurements. Only patients with measurable lesion at baseline should be included in protocols where objective response is the primary endpoint. Measurable lesion is defined by the presence of at least one measurable lesion. In studies where the primary endpoint is tumour progression (either time to progression or proportion with progression at a fixed date), the protocol must specify if entry is restricted to those with measurable disease or whether patients having non-measurable disease only are also eligible.

4.2 Baseline documentation of 'target' and 'non-target' lesions

When more than one measurable lesion is present at baseline, all lesions up to a maximum of five lesions total (and a maximum of two lesions per organ) representative of all involved organs should be identified as target lesions and will be recorded and measured at baseline (this means in instances where patients have only one or two organ sites involved a maximum of two and four lesions respectively will be recorded).

Target lesions should be selected on the basis of their size (lesions with the longest diameter), be representative of all involved organs, but in addition should be those that lend themselves to reproducible repeated measurements. It may be the case that, on occasion, the largest lesion does not lend itself to reproducible measurement in which circumstance the next largest lesion which can be measured reproducibly should be selected.

Lymph nodes merit special mention since they are normal anatomical structures which may be visible by imaging even if not involved by tumor. Pathological nodes which are defined as measurable and may be identified as target lesions must meet the criterion of a short axis of ⩾15 mm by CT scan. Only the short axis of these nodes will contribute to the baseline sum. The short axis of the node is the diameter normally used by radiologists to judge if a node is involved by solid tumor. Nodal size is normally reported as two dimensions in the plane in which the image is obtained (for CT scan this is almost always the axial plane; for MRI the plane of acquisition may be axial, saggital or coronal). The smaller of these measures is the short axis. For example, an abdominal node which is reported as being 20 mm·× 30 mm has a short axis of 20 mm and qualifies as a malignant, measurable node. In this example, 20 mm should be recorded as the node measurement. All other pathological nodes (those with short axis ≥10 mm but <15 mm) should be considered non-target lesions. Nodes that have a short axis <10 mm are considered non-pathological and should not be recorded or followed.

A sum of the diameters (longest for non-nodal lesions, short axis for nodal lesions) for all target lesions will be calculated and reported as the baseline sum diameters. If lymph nodes are to be included in the sum, then as noted above, only the short axis is added into the sum. The baseline sum diameters will be used as reference to further characterise any

objective tumor regression in the measurable dimension of the disease.

All other lesions (or sites of disease) including pathological lymph nodes should be identified as non-target lesions and should also be recorded at baseline. Measurements are not required and these lesions should be followed as ‘present’, ‘absent’, or in rare cases ‘unequivocal progression’. In addition, it is possible to record multiple nontarget lesions involving the same organ as a single item on the case record form (e.g. ‘multiple enlarged pelvic lymph nodes’ or ‘multiple liver metastases’).

4.3 Response criteria

This section provides the definitions of the criteria used to determine objective tumor response for target lesions.

4.3.1 Evaluation of target lesions

Complete Response (CR): Disappearance of all target lesions. Any pathological lymph nodes (whether target or non-target) must have reduction in short axis to <10 mm.

Partial Response (PR): At least a 30% decrease in the sum of diameters of target lesions, taking as reference the baseline sum diameters.

Progressive Disease (PD): At least a 20% increase in the sum of diameters of target lesions, taking as reference the smallest sum on study (this includes the baseline sum if that is the smallest on study). In addition to the relative increase of 20%, the sum must also demonstrate an absolute increase of at least 5 mm. (Note: the appearance of one or more new lesions is also considered progression).

Stable Disease (SD): Neither sufficient shrinkage to qualify for PR nor sufficient increase to qualify for PD, taking as reference the smallest sum diameters while on study.

4.3.2 Special notes on the assessment of target lesions

Lymph nodes. Lymph nodes identified as target lesions should always have the actual short axis measurement recorded (measured in the same anatomical plane as the baseline examination), even if the nodes regress to below 10mm on study. This means that when lymph nodes are included as target lesions, the ‘sum’ of lesions may not be zero even if complete response criteria are met, since a normal lymph node is defined as having a short axis of <10 mm. Case report forms or other data collection methods may therefore be designed to have target nodal lesions recorded in a separate section where, in order to qualify for CR, each node must achieve a short axis <10 mm. For PR, SD and PD, the actual short axis measurement of the nodes is to be included in the sum of target lesions.

Target lesions that become ‘too small to measure’. While on study, all lesions (nodal and non-nodal) recorded at baseline should have their actual measurements recorded at each subsequent evaluation, even when very small (e.g. 2mm). However, sometimes lesions or lymph nodes which are recorded as target lesions at baseline become so faint on CT scan that the radiologist may not feel comfortable assigning an exact measure and may report themas being ‘too small to measure’. When this occurs, it is important that a value be recorded on the case report form. If it is the opinion of the radiologist that the lesion has likely disappeared, the measurement should be recorded as 0 mm. If the lesion is believed to be present and is faintly seen but too small to measure, a default value of 5 mm should be assigned (Note: It is less likely that this rule will be used for lymph nodes since they usually have a definable size when normal and are frequently surrounded by fat such as in the retroperitoneum; however, if a lymph node is believed to be present and is faintly seen but too small to measure, a default value of 5 mm should be assigned in this circumstance as well). This default value is derived from the 5 mm CT slice thickness (but should not be changed with varying CT slice thickness). The measurement of these lesions is potentially non-reproducible, therefore

providing this default value will prevent false responses or progressions based upon measurement error. To reiterate, however, if the radiologist is able to provide an actual measure, that should be recorded, even if it is below 5mm.

Lesions that split or coalesce on treatment. When non-nodal lesions ‘fragment’, the longest diameters of the fragmented portions should be added together to calculate the target lesion sum. Similarly, as lesions coalesce, a plane between them may be maintained that would aid in obtaining maximal diameter measurements of each individual lesion. If the lesions have truly coalesced such that they are no longer separable, the vector of the longest diameter in this instance should be the maximal longest diameter for the ‘coalesced lesion’.

4.3.3 Evaluation of Non-Target Lesions

This section provides the definitions of the criteria used to determine the tumor response for the group of non-target lesions. While some non-target lesions may actually be measurable, they need not be measured and instead should be assessed only qualitatively at the time points specified in the protocol.

Complete Response (CR): Disappearance of all non-target lesions and normalisation of tumor marker level. All the lymph nodes are non-pathological in size (＜10 mm short diameter).

Non-CR/non-PD: Persistence of one or more non-target lesion(s) and/or maintenance of tumor marker level above the normal limits.

Progressive Disease (PD): Unequivocal progression (see comments below) of existing non-target lesions. (Note: the appearance of one or more new lesions is also considered progression).

4.3.4 Special notes on assessment of progression of non-target disease

The concept of progression of non-target disease requires additional explanation as follows: When the patient also has measurable disease. In this setting, to achieve ‘unequivocal progression’ on the basis of the non-target disease, there must be an overall level of substantial worsening in non-target disease such that, even in presence of SD or PR in target disease, the overall tumour burden has increased sufficiently to merit discontinuation of therapy. A modest ‘increase’ in the size of one or more non-target lesions is usually not sufficient to quality for unequivocal progression status. The designation of overall progression solely on the basis of change in non-target disease in the face of SD or PR of target disease will therefore be extremely rare.

When the patient has only non-measurable disease. This circumstance arises in some phase III trials when it is not a criterion of study entry to have measurable disease. The same general concepts apply here as noted above, however, in this instance there is no measurable disease assessment to factor into the interpretation of an increase in non-measurable disease burden. Because worsening in non-target disease cannot be easily quantified (by definition: if all lesions are truly non-measurable) a useful test that can be applied when assessing patients for unequivocal progression is to consider if the increase in overall disease burden based on the change in non-measurable disease is comparable in magnitude to the increase that would be required to declare PD for measurable disease: i.e. an increase in tumor burden representing an additional 73% increase in ‘volume’ (which is equivalent to a 20% increase diameter in a measurable lesion). Examples include an increase in a pleural effusion from ‘trace’ to ‘large’, an increase in lymphangitic disease from localised to widespread, or may be described in protocols as ‘sufficient to require a change in therapy’. If ‘unequivocal progression’ is seen, the patient should be considered to have had overall PD at that point.

While it would be ideal to have objective criteria to apply to non-measurable disease, the very nature of that disease makes it impossible to do so, therefore the increase must be substantial.

4.3.5 New lesions

The appearance of new malignant lesions denotes disease progression; therefore, some comments on detection of new lesions are important. There are no specific criteria for the identification of new radiographic lesions; however, the finding of a new lesion should be unequivocal: i.e. not attributable to differences in scanning technique, change in imaging modality or findings thought to represent something other than tumor (for example, some 'new' bone lesions may be simply healing or flare of pre-existing lesions). This is particularly

important when the patient's baseline lesions show partial or complete response. For example, necrosis of a liver lesion may be reported on a CT scan report as a 'new' cystic lesion, which it is not. A lesion identified on a follow-up study in an anatomical location that was not scanned at baseline is considered a new lesion and will indicate disease progression. An example of this is the patient who has visceral disease at baseline and while on study has a CT or MRI brain ordered which reveals metastases. The patient's brain metastases are considered to be evidence of PD even if he/she did not have brain imaging at baseline. If a new lesion is equivocal, for example because of its small size, continued therapy and follow-up evaluation will clarify if it represents truly new disease. If repeat scans confirm there is definitely a new lesion, then progression should be declared using the date of the initial scan. While FDG-PET response assessments need additional study, it is sometimes reasonable to incorporate the use of FDG-PET scanning to complement CT scanning in assessment of progression (particularly possible 'new' disease). New lesions on the basis of FDG-PET imaging can be identified according to the following algorithm:

a. Negative FDG-PET at baseline, with a positive FDG-PET at follow-up is a sign of PD based on a new lesion.

b. No FDG-PET at baseline and a positive FDG-PET at follow-up:

If the positive FDG-PET at follow-up corresponds to a new site of disease confirmed by CT, this is PD.

If the positive FDG-PET at follow-up is not confirmed as a new site of disease on CT, additional follow-up CT scans are needed to determine if there is truly progression occurring at that site (if so, the date of PD will be the date of the initial abnormal FDG-PET scan).

If the positive FDG-PET at follow-up corresponds to a pre-existing site of disease on CT that is not progressing on the basis of the anatomic images, this is not PD.

4.4 Evaluation of Best Overall Response

The best overall response is the best response recorded from the start of the study treatment until the end of treatment taking into account any requirement for confirmation. On occasion a response may not be documented until after the end of therapy so protocols should be clear if post-treatment assessments are to be considered in determination of best overall response. Protocols must specify how any new therapy introduced before progression will affect best response designation. The patient's best overall response assignment will depend on the findings of both target and non-target disease and will also take into consideration the appearance of new lesions. Furthermore, depending on the nature of the study and the protocol requirements, it may also require confirmatory measurement. Specifically, in non-randomised trials where response is the primary endpoint, confirmation of PR or CR is needed to deem either one the 'best overall response'.

4.4.1 Time point response

It is assumed that at each protocol specified time point, a response assessment occurs. Table 1 on the next page provides a summary of the overall response status calculation at each time point for patients who have measurable disease at baseline.

When patients have non-measurable (therefore non-target) disease only, Table 2 is to be used.

4.4.2 Missing Assessments and inevaluable Designation

When no imaging/measurement is done at a particular time point, the patient is not evaluable (NE) at that time point. If only a subset of lesion measurements are made at an assessment, usually the case is also considered NE at that time point, unless a convincing argument can be made that the contribution of the individual missing lesion(s) would not change the assigned time point response. This would be most likely to happen in the case of PD. For example, if a patient had a baseline sum of 50 mm with three measured lesions and at follow-up only two lesions were assessed, but those gave a sum of 80 mm, the patient will have achieved PD status, regardless of the contribution of the missing lesion.

4.4.3 Best overall response: all time points

The best overall response is determined once all the data for the patient is known.

Best response determination in trials where confirmation of complete or partial response IS NOT required: Best response in these trials is defined as the best response across all time points (for example, a patient who has SD at first assessment, PR at second assessment, and PD on last assessment has a best overall response of PR). When SD is believed to be best response, it must also meet the protocol specified minimum time from baseline. If the minimum time is not met when SD is otherwise the best time point response, the patient's best response depends on the subsequent assessments. For example, a patient who has SD at first assessment, PD at second and does not meet minimum duration for SD, will have a best response of PD. The same patient lost to follow-up after the first SD assessment would be considered inevaluable.

Best response determination in trials where confirmation of complete or partial response IS required: Complete or partial responses may be claimed only if the criteria for each are met at a subsequent time point as specified in the protocol (generally 4 weeks later). In this circumstance, the best overall response can be interpreted as in Table 3. .

4.4.4 Special notes on response assessment

When nodal disease is included in the sum of target lesions and the nodes decrease to 'normal' size (<10 mm), they may still have a measurement reported on scans. This measurement should be recorded even though the nodes are normal in order not to overstate progression should it be based on increase in size of the nodes. As noted earlier, this means that patients with CR may not have a total sum of 'zero' on the case report form (CRF). In trials where confirmation of response is required, repeated 'NE' time point assessments may complicate best response determination. The analysis plan for the trial must address how missing data/assessments will be addressed in determination of response and progression. For example, in most trials it is reasonable to consider a patient with time point responses of PR-NE-PR as a confirmed response. Patients with a global deterioration of health status requiring discontinuation of treatment without objective evidence of disease progression at that time should be reported as 'symptomatic deterioration'. Every effort should be made to document objective progression even after discontinuation of treatment. Symptomatic deterioration is not a descriptor of an objective response: it is a reason for stopping study therapy. The objective response status of such patients is to be determined by evaluation of target and non-

target disease as shown in Tables 1-3.

Conditions that define 'early progression, early death and inevaluability' are study specific and should be clearly described in each protocol (depending on treatment duration, treatment periodicity).

In some circumstances it may be difficult to distinguish residual disease from normal tissue. When the evaluation of complete response depends upon this determination, it is recommended that the residual lesion be investigated (fine needle aspirate/biopsy) before assigning a status of complete response. FDG-PET may be used to upgrade a response to a CR in a manner similar to a biopsy in cases where a residual radiographic abnormality is thought to represent fibrosis or scarring. The use of FDG-PET in this circumstance should be prospectively described in the protocol and supported by disease specific medical literature for the indication. However, it must be acknowledged that both approaches may lead to false positive CR due to limitations of FDG-PET and biopsy resolution/sensitivity.

For equivocal findings of progression (e.g., very small and uncertain new lesions; cystic changes or necrosis in existing lesions), treatment may continue until the next scheduled assessment. If at the next scheduled assessment, progression is confirmed, the date of progression should be the earlier date when progression was suspected.

Table 1 Time point response - patients with target (+/- non-target) disease.

**Target lesions**	**Non-target lesions**	**New lesions**	**Overall response**
CR	CR	No	CR
CR	Non-CR/non-PD	No	PR
CR	Not evaluated	No	PR
PR	Non-PD or not all evaluated	No	PR
SD	Non-PD or not all evaluated	No	SD
Not all evaluated	Non-PD	No	NE
PD	Any	Yes or No	PD
Any	PD	Yes or No	PD
Any	Any	Yes	PD
CR = complete response, PR = partial response, SD = stable disease, PD= progressive disease, and NE = inevaluable.			

The SD cases must meet the SD standard in at least one follow-up measurement after enrollment, and the interval between follow-up and enrollment should be at least 6~8 weeks.

Table 2 Time point response - patients with non-target disease only.

**Non-target lesions**	**New lesions**	**Overall response**
CR	No	CR
Non-CR or non-PD	No	Non-CR / non-PD[a]
Not all evaluated	No	NE
Unequivocal PD	Yes or No	PD
Any	Yes	PD
CR = complete response, PD= progressive disease, and NE = inevaluable.<br>a 'Non-CR/non-PD' is preferred over 'stable disease' for non-target disease since		

SD is increasingly used as endpoint for assessment of efficacy in some trials so to assign this category when no lesions can be measured is not advised.

Table 3 Best overall response when confirmation of CR and PR required.

Overall response first time point	Overall response subsequent time point	Best overall response
CR	CR	CR
CR	PR	SD, PD or PR[a]
CR	SD	SD provided minimum criteria for SD duration met, otherwise, PD
CR	PD	SD provided minimum criteria for SD duration met, otherwise, PD
CR	NE	SD provided minimum criteria for SD duration met, otherwise NE
PR	CR	PR
PR	PR	PR
PR	SD	SD
PR	PD	SD provided t minimum criteria for SD duration met, otherwise, PD
PR	NE	SD provided minimum criteria for SD duration met, otherwise NE
NE	NE	NE

CR = complete response, PR = partial response, SD = stable disease, PD= progressive disease, and NE = inevaluable.
a If a CR is truly met at first time point, then any disease seen at a subsequent time point, even disease meeting PR criteria relative to baseline, makes the disease PD at that point (since disease must have reappeared after CR). Best response would depend on whether minimum duration for SD was met. However, sometimes ‘CR’ may be claimed when subsequent scans suggest small lesions were likely still present and in fact the patient had PR, not CR at the first time point. Under these circumstances, the original CR should be changed to PR and the best response is PR.

4.5 Frequency of tumor re-evaluation

Frequency of tumor re-evaluation while on treatment should be protocol specific and adapted to the type and schedule of treatment. However, in the context of phase II studies where the beneficial effect of therapy is not known, follow-up every 6-8 weeks (timed to coincide with the end of a cycle) is reasonable. Smaller or greater time intervals than these could be justified in specific regimens or circumstances. The protocol should specify which organ sites are to be evaluated at baseline (usually those most likely to be involved with metastatic disease for the tumor type under study) and how often evaluations are repeated. Normally, all target and non-target sites are evaluated at each assessment. In selected circumstances certain non-target organs may be evaluated less frequently. For example, bone scans may need to be repeated only when complete response is identified in target disease or when progression in bone is suspected.

After the end of the treatment, the need for repetitive tumor evaluations depends on

whether the trial has as a goal the response rate or the time to an event (progression/death). If ‘time to an event’ (e.g. time to progression, disease-free survival, progression-free survival) is the main endpoint of the study, then routine scheduled re-evaluation of protocol specified sites of disease is warranted. In randomised comparative trials in particular, the scheduled assessments should be performed as identified on a calendar schedule (for example: every 6-8 weeks on treatment or every 3-4 months after treatment) and should not be affected by delays in therapy, drug holidays or any other events that might lead to imbalance in a treatment arm in the timing of disease assessment.

4.6 Confirmatory measurement/duration of response

4.6.1 Confirmation

In non-randomised trials where response is the primary endpoint, confirmation of PR and CR is required to ensure responses identified are not the result of measurement error. This will also permit appropriate interpretation of results in the context of historical data where response has traditionally required confirmation in such trials. However, in all other circumstances, i.e. in randomised trials (phase II or III) or studies where stable disease or progression are the primary endpoints, confirmation of response is not required since it will not add value to the interpretation of trial results. However, elimination of the requirement for response confirmation may increase the importance of central review to protect against bias, in particular in studies which are not blinded.

In the case of SD, measurements must have met the SD criteria at least once after study entry at a minimum interval (in general not less than 6–8 weeks) that is defined in the study protocol.

4.6.2 Duration of overall response

The duration of overall response is measured from the time measurement criteria are first met for CR/PR (whichever is first recorded) until the first date that recurrent or progressive disease is objectively documented (taking as reference for progressive disease the smallest measurements recorded on study).

The duration of overall complete response is measured from the time measurement criteria are first met for CR until the first date that recurrent disease is objectively documented.

4.6.3 Duration of stable disease

Stable disease is measured from the start of the treatment (in randomised trials, from date of randomisation) until the criteria for progression are met, taking as reference the smallest sum on study (if the baseline sum is the smallest, this is the reference for calculation of PD).

The clinical relevance of the duration of stable disease varies in different studies and diseases. If the proportion of patients achieving stable disease for a minimum period of time is an endpoint of importance in a particular trial, the protocol should specify the minimal time interval required between two measurements for determination of stable disease.

Note: The duration of response and stable disease as well as the progression-free survival are influencedby the frequency of follow-up after baseline evaluation. It is not in the scope of this guideline to define a standard follow-up frequency. The frequency should take into account many parameters including disease types and stages, treatment periodicity and standard practice. However, these limitations of the precision of the measured endpoint should be taken into account if comparisons between trials are to be made.

4.7 Progression-free survival/proportion of progression-free survival (PPF)

4.7.1 Phase II trials

This guideline is focused primarily on the use of objective response endpoints for phase II trials. In some circumstances, ‘response rate’ may not be the optimal method to assess the potential anticancer activity of new agents/regimens. In such cases ‘progression-free survival’ (PFS) or the ‘proportion progression-free’ at landmark time points, might be considered appropriate alternatives to provide an initial signal of biologic effect of new agents. It is clear, however, that in an uncontrolled trial, these measures are subject to criticism since an apparently promising observation may be related to biological factors such as patient selection and not the impact of the intervention. Thus, phase II screening trials utilising these endpoints are best designed with a randomised control. Exceptions may exist where the behaviour patterns of certain cancers are so consistent (and usually consistently poor), that a non-randomised trial is justifiable. However, in these cases it will be essential to document with care the basis for estimating the expected PFS or proportion progression-free in the absence of a treatment effect.

Note: The age unit is year, and the weight unit is kg.

Appendix IV Strong and Moderate Inducers of CYP3A4 and Strong Inducer and Inhibitor of P-gp

Drug type	Drugs
Strong Inducer of CYP3A4	Phenytoin, phenobarbital, rifampicin, carbamazepine, rifabutin, rifapentine and St. John's wort
Moderate Inducer of CYP3A4	Bosentan, efavirenz, etravirine, nafcillin and modafinil
Strong Inducer of P-gp	Phenytoin, rifampicin, carbamazepine, phenobarbital and Hypericum perforatum
Strong Inhibitor of P-gp	Ritonavir, ciclosporin A, ketoconazole, clarithromycin, atazanavir, nefazodone, saquinavir, telithromycin, itraconazole, erythrocin, indinavir, nafenavir, verapamil, quinidine, tacrolimus, nelfinavir and amiodarone

Appendix V Revision 7 of International TNM Classification and Staging of Lung Cancer

Stage T: Tumour

TX: Primary tumour cannot be assessed, or tumour proven by the presence of malignant cells in sputum or bronchial washings but not visualized by imaging or bronchoscopy.

T0: No evidence of primary tumour.

Tis: Carcinoma in situ

T1: Tumour < 3 cm in greatest dimension, surrounded by lung or visceral pleura, without bronchoscopic evidence of invasion more proximal than the lobar bronchus (i.e., not in the main bronchus) .

T1a: Tumour < 2 cm in greatest dimension;

T1b: Tumour > 2 cm but < 3 cm in greatest dimension.

T2: Tumour > 3 cm but < 7 cm or tumour with any of the following features (T2 tumours with these features are classified T2a if < 5 cm): Involves main bronchus, > 2 cm distal to the carina; Invades visceral pleura; Associated with atelectasis or obstructive pneumonitis that extends to the hilar region but does not involve the entire lung.

T2a: Tumour > 3 cm but < 5 cm in greatest dimension;

T2b: Tumour > 5 cm but < 7 cm in greatest dimension.

T3: Tumour > 7 cm or one that directly invades any of the following: Chest wall (including superior sulcus tumours), diaphragm, phrenic nerve, mediastinal pleura, parietal pericardium; Tumour in the main bronchus < 2 cm distal to the carina but without involvement of the carina; Associated atelectasis or obstructive pneumonitis of the entire lung; Separate tumour nodule(s) in the same lobe.

T4: Tumour of any size that invades any of the following: Mediastinum, heart, great vessels, trachea, recurrent laryngeal nerve, esophagus, vertebral body, carina; Separate tumour nodule(s) in a different ipsilateral lobe.

Stage N: Nodes

NX: Regional lymph nodes cannot be assessed;

N0: No regional lymph node metastasis;

N1: Metastasis in ipsilateral peribronchial and/or ipsilateral hilar lymph nodes and intrapulmonary nodes, including involvement by direct extension;

N2: Metastasis in ipsilateral mediastinal and/or subcarinal lymph node(s);

N3: Metastasis in contralateral mediastinal, contralateral hilar, ipsilateral or contralateral scalene, or supraclavicular lymph node(s)

Stage M: Metastases

MX: Distant metastasis cannot be assessed;

M0: No distant metastasis;

M1: Distant metastasis

M1a: Pleural dissemination (malignant pleural effusion, pericardial effusion or pleural nodules) and cancerous node in the contralateral pulmonary lobe (pleural effusions in many lung cancers are caused by tumor, and pleural fluid are negative in repeated cytology tests in a few patients, i.e., they are neither bloody fluid nor effusion. If effusion is unrelated to the tumor as judged by various factors and clinical practices, pleural effusion should not be included in the staging factors, and the patients should still be divided into T1-3).

M1b: Pulmonary and extra-pleural distant metastasis

TNM staging of Edition 7 after revision

	**T**	**N**	**M**
Stage 0	Tis	N0	M0
IA	T1a,b	N0	M0
IB	T2a	N0	M0
IIA	T1a,b	N1	M0
	T2a	N1	M0
	T2b	N0	M0
IIB	T2b	N1	M0
	T3	N0	M0
IIIA	T1,T2	N2	M0
	T3	N1, N2	M0
	T4	N0, N1	M0
IIIB	T4	N2	M0
	Any Stage T	N3	M0
IV	Any Stage T	Any Stage N	M1a, M1b

Statistical Analysis Plan (SAP)

Protocol name:	A Phase Ib Clinical Study to Evaluate the Safety and Efficacy of JMT101 in Combination with Afatinib or Osimertinib in Stage IIIB or IV Non-small Cell Lung Cancer Patients with EGFR Exon 20 Insertion Mutations
Protocol No.:	JMT101-CSP-001
Protocol version/date:	V3.0, December 10, 2020
Sponsor:	Shanghai JMT-Bio Inc.
Statistical Analysis Unit:	Meta Clinical Technology Co, Ltd (Nanjing)
SAP Author:	Gao Wanjun
SAP Version:	V0.3
SAP Date:	March 17, 2021

Signature of Sponsor:

Approved by: ______________________

Signature: ______________________ Date: ______________

Title: ______________________

Unit name: Shanghai JMT-Bio Inc.

Signature of Meta Clinical Technology Co, Ltd (Nanjing):

Written by: Gao Wanjun

Signature: ______________________ Date: ______________

Title: Statistician

Unit name: Meta Clinical Technology Co, Ltd (Nanjing)

Approved by: ______________________

Signature: ______________________ Date: ______________

Title: Statistician

Unit name: Meta Clinical Technology Co, Ltd (Nanjing)

Table of Contents

1. Description of abbreviations and statistics used in this text (in English)..............5
2. Introduction..............6
3. Study Objectives..............6
3.1 Main Objective..............6
3.2 Secondary Objective..............6
4. Study Design..............6
4.1 Overall Study Design..............6
4.2 Definition of Dose-Limiting Toxicity..............9
4.3 Definition of Maximum Tolerated Dose..............10
4.4 Definition of End of Study..............10
4.5 Sample Size..............10
4.6 Blinding and randomization..............11
5. Evaluation Parameters..............11
5.1 Safety Assessment..............11
5.2 Efficacy assessment..............11
5.3 Immunogenicity parameters..............12
5.4 Biomarker parameters..............12
6. Statistical analysis methods..............12
6.1 General Principles..............12
6.2 Statistical Analysis Dataset..............13
6.3 Subject Disposition..............13
6.4 Demographic and Baseline Characteristics..............14
6.5 Previous / concomitant medications and previous / concomitant therapy..............15
6.6 Protocol violation..............16
6.7 Efficacy Evaluation Analysis..............16
6.8 Safety Analysis..............19
6.8.1 Drug exposure..............19
6.8.2 DLT..............20
6.8.3 Adverse event..............20
6.8.4 Laboratory tests..............22
6.8.5 12-lead ECG..............24
6.8.6 Physical examination..............24
6.8.7 Vital sign..............25
6.8.8 ECOG score..............25
6.8.9 Echocardiography..............26
6.8.10 Pregnancy test..............26
6.9 Immunogenicity Study..............26
6.10 Biomarker parameter analysis..............26
6.11 Handling of missing values..............27
6.12 Processing of outlier..............28

7. Interim Analysis....................28
8. About This Plan....................28

1. DESCRIPTION OF ABBREVIATIONS AND STATISTICS USED IN THIS TEXT (IN ENGLISH)

AE	Adverse Event
ATC	Anatomical Therapeutic Chemical
CR	Complete Response
CRF	Case Report Form
CTCAE	Common Terminology Criteria for Adverse Events
DCR	Disease Control Rate
DLT	Dose Limiting Toxicity
eCRF	Electrical Case Report Forms
ECOG	Eastern Cooperative Oncology Group
EGFR	Epidermal Growth Factor Receptor
FAS	Full Analysis Set
Max	Maximum
Mean	Mean
MedDRA	Medical Dictionary for Regulatory Activities
Median	Median
Min	Minimum
MTD	Maximum Tolerated Dose
NSCLC	Non Small Cell Lung Cancer
ORR	Objective Response Rate
PD	Progressive Disease
PFS	Progression-Free-Survival
PPS	Per-protocol Set
PT	Preferred Term
PR	Partial Response
RECIST	Response Evaluation Criteria in Solid Tumors
SAE	Serious Adverse Event
SAP	Statistical Analysis Plan
SD	Standard Deviation
SD	Stable Disease
SOC	System Organ Class
SS	Safety Set
TEAE	Treatment Emergent Adverse Event
TLF	Table Listing Figure

2. INTRODUCTION

This statistical analysis plan (SAP) describes the safety and tolerability, baseline characteristics, preliminary efficacy evaluation plan analysis and reported content of the study (A phase Ib clinical study to evaluate the safety and efficacy of JMT101 in combination with Afatinib or Osimertinib in stage IIIB or IV non-small cell lung cancer patients with EGFR exon 20 insertion mutations). SAP main reference documents:

- Study protocol: V3.0/December 10, 2020;
- eCRF: V3.0/March 3, 2021.

3. STUDY OBJECTIVES

3.1 Main Objective

- To evaluate the safety and tolerability of JMT101 in combination with Afatinib or Osimertinib in stage IIIB or IV non-small cell lung cancer (NSCLC) patients with EGFR exon 20 insertion mutations.

3.2 Secondary Objective

- To evaluate the efficacy of JMT101 in combination with Afatinib or Osimertinib in stage IIIB or IV NSCLC patients with EGFR exon 20 insertion mutations, so as to provide a basis for the recommended dosing regimen for subsequent studies.
- To evaluate the pharmacokinetic profile of JMT101.
- To evaluate the immunogenicity of JMT101.
- To analyze possible correlations between biomarkers and clinical outcomes.

4. STUDY DESIGN

4.1 Overall Study Design

This is a multi-center, open-label, dose-escalation phase Ib clinical study in stage IIIB or IV NSCLC patients, which aims to evaluate the safety, tolerability and efficacy of JMT101 in combination with Afatinib or Osimertinib in patients with stage IIIB or IV NSCLC harboring EGFR exon 20 insertion mutations, to provide a basis for the recommended dosing regimen for subsequent studies, to evaluate the pharmacokinetic profile and immunogenicity of JMT101, and to explore tumor-associated biomarkers.

This study is divided into two stages: the first stage (Stage I) is a dose escalation study and the second stage (Stage II) is a dose expansion study.

Stage I dose escalation study:

This study adopts a combination therapy regimen, which divides the patients into Group A and Group B according to different combined drugs: Group A is treated with JMT101 in combination with Afatinib and Group B is treated with JMT101 in combination with Osimertinib. Each group is divided into two cohorts according to the administered dose, and a total of 4 cohorts are established as follows:

A1 cohort: JMT101 6 mg/kg, intravenous drip, Q2W + Afatinib 30 mg, oral, QD;

A2 cohort: JMT101 6 mg/kg, intravenous drip, Q2W + Afatinib 40 mg, oral, QD;

B1 cohort: JMT101 6 mg/kg, intravenous drip, Q2W + Osimertinib 80 mg, oral, QD;

B2 cohort: JMT101 6 mg/kg, intravenous drip, Q2W + Osimertinib 160 mg, oral, QD;

The dose escalation follows the “3 + 3 principle”. Each cohort includes 3-6 subjects to observe safety and tolerability. Subjects with asymptomatic central nervous system (CNS) metastasis or meningeal metastasis are enrolled in Group A or B at the discretion of the investigator, and the rest of the subjects are enrolled into Group A and B in sequence.

Subjects are first enrolled in A1 and B1 cohorts, and each of the cohorts shall have at least 3 evaluable subjects. Subjects can be enrolled in higher dose cohorts (A2 or B2) only if no dose-limiting toxicity (DLT) is observed in 3 subjects in cohorts A1 or B1 during the DLT observation period(Cycle 1, Day 1 to 28). If DLT occurs in 1 of 3 subjects in one cohort, 3 additional subjects need to be added to the same cohort (the cohort has 6 evaluable subjects). If no DLT occurs in the 3 additional subjects, the subjects will continue to be enrolled in the higher dose cohort; if DLT occurs in 1 or more of the 3 additional subjects or in 2 or more of the 6 subjects in total, the planned higher dose cohort enrollment will not be conducted. If DLT occurs in 2 of 3 subjects in one cohort, the planned higher dose cohort enrollment will not be conducted.

If drug intolerance occurs in the initial dose cohort (A1 or B1) in a group, the investigator and sponsor are required to discuss jointly to decide whether to proceed with a cohort that is one dose level down from JMT101 (i.e., JMT101 4 mg/kg Q2W, with no

change in combined drug dose) or to stay in the initial dose cohort and continue enrolling subjects until excessive toxicity occurs or early discontinuation of the study in the group. If the safety and tolerability of this dose group remains good when the dose is escalated to the predetermined maximum dose (cohort A2 or B2), the decision to explore JMT101 up one dose level cohort (i.e., JMT101 8 mg/kg Q2W with no change in combined drug dose) may be discussed between the investigator and the sponsor..

The previous dose level of the dose limiting toxicity (DLT) dose level is defined as the maximum tolerated dose (MTD). At least 6 subjects are enrolled and evaluated in the MTD dose group.

Stage II dose expansion study:

According to the safety, tolerability and efficacy data obtained in Stage I, the dose expansion study is carried out for the target dose cohort. All cohorts with good safety and tolerability in Stage I (up to 4 cohorts) are selected in which a certain number of additional subjects are includedto further explore safety, tolerability, pharmacokinetic profile and anti-tumor activity. Subjects with asymptomatic central nervous system (CNS) metastasis or meningeal metastasis are enrolled in Group A or B at the discretion of the investigator, and the rest of the subjects are enrolled into each cohort in a certain order. Safety and efficacy data are monitored periodically during the course of the study. Considering the subject benefits, if a cohort shows clear evidence of treatment disadvantage, the enrollment into the cohort should be closed in advance to avoid more subjects receiving ineffective or low effective treatment; if a cohort shows clear evidence of treatment benefits, the recruiting number may be increased in that cohort. A minimum of 12 subjects and a maximum of 200 subjects are expected to be enrolled in the dose escalation study and the dose expansion study. For all the above subjects, every 4 weeks (28 days) is considered as a treatment cycle during the treatment period, and treatment continues until one of the following occurs: progressive disease, unacceptable toxicity, subject's withdrawal request, or absence of further benefit from treatment judged by the investigator, whichever occurs first. The dosing regimen may be adjusted based on the subject's toxicity reaction (the dosing regimen are not allowed to be modified for the subjects during the DLT observation period in Stage I). Safety inspections

are performed during treatment as required by the protocol, and tumor assessments are performed at the end of Cycle 1 and every two cycles thereafter (Cycle 3, cycle 5 ....) (If tumor-associated symptoms worsen and the investigator deems it necessary, the duration of tumor assessment may be shortened). After the last dose of investigational drug, subjects should be followed up for survival every 8 weeks, and for adverse events still present at the end-of-study visit.

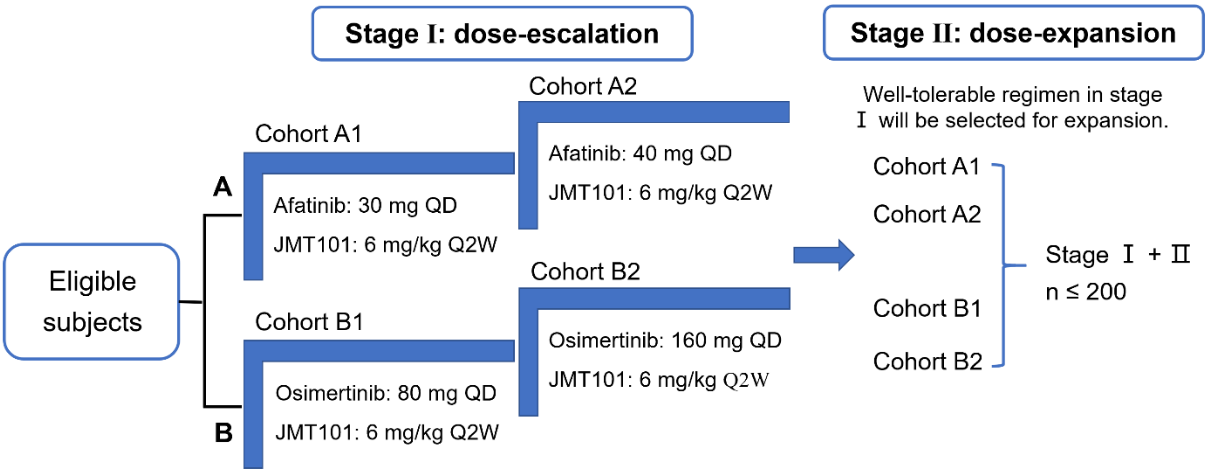

Figure 1 Schema of the overall study process

4.2 Definition of Dose-Limiting Toxicity

DLT is defined as: one or more of the following toxic reactions judged to be reasonably related (related, probably related, and possibly related) to the investigational drug that occurs within Cycle 1 (Day 1 to 28) of study dosing.

(1) Non-hematological toxicity:

· Grade 4 rash or Grade 3 rash that leads to 4 weeks of suspension, or Grade 3 rash with severe infection.

· Grade 4 diarrhea or Grade 3 diarrhea that has not resolved after 2 weeks of suspension, or Grade 3 diarrhea that reappears after appropriate supportive therapy.

· Any non-hematologic toxicity of grade 3 or greater other than rash and diarrhea (nausea/vomiting, constipation and electrolyte imbalance are considered as DLT only if they remain ≥ grade 3 after appropriate supportive treatment).

· Development of interstitial pneumonia or pulmonary fibrosis.

(2) Hematological toxicity:

- Grade 4 neutropenia that persists for more than 5 days.
- Febrile neutropenia (ANC < 1.0 x 10^9/L with temperature of 38.3°C (axillary temperature) in a single measurement or temperature ≥ 38°C (axillary temperature) for more than one hour).
- Grade 3 neutropenia with evidence of infection.
- Grade 3 thrombocytopenia with clinically significant hemorrhage.
- Grade 4 thrombocytopenia.
- Grade 4 anaemia (life threatening).

(3) Other toxic reactions that, in the judgment of the investigator, should result in permanent discontinuation of the investigational drug.

All adverse events will be graded according to the National Cancer Institute Common Terminology Criteria for Adverse Events (NCI-CTCAE) version 5.0.

4.3 Definition of Maximum Tolerated Dose

The previous dose level of the dose-limiting toxicity (DLT) dose level is defined as MTD. At least 6 subjects are enrolled and evaluated in the MTD dose group.

4.4 Definition of End of Study

A subject is considered to have completed the study if he or she has completed all stages of the study, including the last visit or the last planned procedure listed in the schedule of activities.

End of study is defined as the completion of the last visit or the completion of all steps listed in the schedule of activities.

4.5 Sample Size

Based on the defined cohort: Stage I: The dose escalation study is expected to enroll a minimum of 12 subjects. Stage II: According to the existing study results, the expected ORR of the combination therapy cohort is about 40%, and when the sample size is 90 subjects, the probability of observed ORR>35% calculated by normal approximation method is 83.4%; if the expected ORR is lower than 40%, the probability of observed ORR>35% shows a decreasing trend. Together with subjects in the dose-escalation stage, the total number of

subjects enrolled in all the cohorts is not more than 200. Safety and efficacy data are monitored periodically during the course of the study. Considering the subject benefits, if a cohort shows clear evidence of treatment disadvantage, the enrollment into the cohort should be closed in advance to avoid more subjects receiving ineffective or low effective treatment; if a cohort shows clear evidence of treatment benefits, an increase in the number of subjects enrolled in that cohort may be considered.

A minimum of 12 subjects and a maximum of 200 subjects are expected to be enrolled in the whole study.

4.6 Blinding and randomization

Not applicable.

5. EVALUATION PARAMETERS

5.1 Safety Assessment

- Dose-limiting toxicity (DLT) and maximum tolerated dose (MTD).
- The safety assessment will be carried out throughout the study by evaluating the subject's vital signs, weight, physical examination, ECOG score, electrocardiogram and laboratory tests. The severity of adverse events is assessed according to NCI-CTCAE V5.0.

5.2 Efficacy assessment

Objective response rate (ORR): defined as the proportion of patients with complete response (CR) or partial response (PR) (i.e., CR+PR) at the optimal time point from initiation of investigational drug to study withdrawal, as evaluated by the Independent Review Committee (IRC) and the investigator according to the Response Evaluation Criteria in Solid Tumors (RECIST v1.1). The first occurrence of CR and PR needs to be confirmed. Tumor response evaluated after the initiation of new anti-tumor therapy should not be included into the evaluation of the best response.

Duration of remission (DOR): defined as the time from the first documentation of objective response to the first documentation of PD or death from any cause before PD, reflecting the duration of ORR.

Disease control rate (DCR): defined as the proportion of patients with responses of CR,

PR, and stable disease (SD) (i.e., CR+PR+SD) at the optimal time point from initiation of investigational drug to study withdrawal, as evaluated by RECIST 1.1 criteria. Tumor response evaluated after the initiation of new anti-tumor therapy should not be included into the evaluation of response at the optimal time point.

Progression-free survival (PFS): defined as the time from initiation of investigational drug to the date of first recorded progression of disease (PD) or death, depending on which occurs first.

Overall survival (OS): defined as the time from initiation of investigational drug to the date of death from any cause.

5.3 Immunogenicity parameters

Immunogenicity test timeline: Before the 1st dose, 15 days (±1 day) after first dosing (before the 2nd dose), 29 days after first dosing (±1 day) (before the 3rd dose), 57 days after first dosing (±3 days) (before the 5th dose) and the last visit (30 ±3 days after the last dose). Blood is collected within 30 minutes prior to each dose, approximately 3.5 mL per collection. To evaluate the immunogenicity of JMT101, samples are first tested for anti-drug antibodies. Samples that are tested positive for anti-drug antibodies should be further tested for neutralizing antibodies.

5.4 Biomarker parameters

Tumor-associated biomarkers such as serum free DNA are explored to analyze possible correlations between biomarkers and clinical outcomes.

6. STATISTICAL ANALYSIS METHODS

6.1 General Principles

Statistical analysis is performed using SAS 9.4 (or higher) statistical software. The descriptive statistics of the measurement data include mean, standard deviation, median, quartiles, maximum and minimum values. The descriptive statistics of count data or grade data include the number of cases and percentage.

In general, the maximum and minimum values have the same number of decimal places

as the original data; the mean, median and quartiles have 1 more decimal place than the original data; the standard deviation has 2 more decimal places than the original data, and all decimals do not exceed 4 places. When the number of decimal places exceeds 4, 4 significant digits after the decimal point are retained. Percentages are retained to 1 decimal place. Percentages are not displayed when the frequency is 0, and the number of decimal places of the confidence interval is retained to 2. Changes in categorical data from the baseline are described using a shift table. The number of decimal places to be retained can be adjusted according to the actual situation.

6.2 Statistical Analysis Dataset

- Full Analysis Set (FAS): a collection of all subjects that have been successfully enrolled and used at least one dose of investigational drug.
- Per-protocol Set (PPS): a collection of subjects that meet the inclusion criteria, do not meet the exclusion criteria, and complete the treatment regime， the set of all subjects who meet the study protocol, have good compliance, complete the treatment specified in the protocol, and have no major protocol deviation in the process are analyzed.
- Safety analysis set (Safety Set, SS): All enrolled subjects who have used at least one dose of investigational drug and have post-dosing safety records are included in the safety analysis set.
- DLT Analysis Set (DLT Set): The DLT Set includes all subjects in the DLT assessment period (1 to 28 d), subjects who complete the DLT assessment or those who withdraw from the trial early due to adverse events during the DLT assessment period (subjects whose DLT is not evaluable due to intolerance to non-investigational drug dose will not be included in the DLT statistical sample). This analysis set will be used to analyze and summarize DLT events.
- Immunogenicity analysis set: Subjects who have received at least one dose of investigational drug and have at least one sample of anti-drug antibodies collected.
- Biomarker analysis set: Subjects who have received at least one dose of investigational drug and have at least 1 tumor tissue sample or at least one biomarker blood sample collected.

6.3 Subject Disposition

The total number of subjects screened, screening failures and primary reasons for screen

failureare summarized; the number of subjects screened, enrolled, and treated for each cohort and for each analysis data set are summarized by center and centers combined. The treatment received, the end of treatment situation and its reasons, and the end of study situation and its reasons are described. Reasons for end of treatment include adverse events, disease progression, pregnancy, death, poor protocol compliance, lost to follow-up, change of treatment regimen, study termination by the sponsor, investigator’s decision to discontinue, and subject’s unwillingness to continue participation in the study. End of study includes death, lost to follow-up, subject’s unwillingness to continue participation in the study, failure to meet inclusion criteria/exclusion criteria, completion of the entire study, and study termination by the sponsor. A list of data on the inclusion of enrolled subjects in each analysis set is presented, and subjects not included in the analysis set need to be marked with the reason for non-inclusion. The list of end-of-treatment subjects and the list of end-of-study subjects are presented.

6.4 Demographic and Baseline Characteristics

Baseline measurement is defined as the last assessment performed before the first dose. Demographic and baseline characteristics are analyzed based on FAS subcohorts, groups, and all subjects. According to the data type: the descriptive statistics of count data and grade data include number of cases and percentage (%); the descriptive statistics of measurement data include number of cases, mean, standard deviation, median, quartiles, minimum and maximum values etc. In the table, abnormal/- means abnormal but not clinically significant, and abnormal/+ means abnormal and clinically significant. Baseline characteristics include:

- General demographic characteristics of the subjects: gender, age, ethnicity, height, weight, BMI;
- Tumor diagnosis: disease course, histopathological staging, TNM stage of first diagnosis, clinical stage of first diagnosis, current TNM stage, current clinical stage, where disease course = date of signed informed consent - date of initial diagnosis + 1.
- Pre-existing EGFR gene testing: type of sampling, presence of EGFR exon 20 insertion mutation, and whether other mutations are combined.
- Past and present medical history: Past and present medical history is coded according to

System Organ Class (SOC) and Preferred Term (PT) using the Chinese version of the Medical Dictionary for Regulatory Activities (MedDRA) (23.0 or above), and the number and percentage of subjects with at least one past medical history are summarized according to SOC and PT.

- Personal history: history of smoking, alcohol abuse, drug abuse.
- Previous history of anti-tumor treatment: surgical treatment history, drug treatment history, radiotherapy history and other treatment history; The drug treatment history is subdivided to summarize the number and percentage of subjects with prior use of chemotherapeutic agents, anti-angiogenic targeted agents, immunotherapeutic drugs, EGFR-TKIs, TAK-788, and Poziotinib, respectively.
- Virological screening: including 5 markers of hepatitis B (hepatitis B surface antigen (HBsAg), hepatitis B surface antibody (HBsAb), hepatitis B e antigen (HBeAg), hepatitis B e antibody (HBeAb), hepatitis B core antibody (HBcAb)), hepatitis C virus antibody (HCV-Ab), human immunodeficiency virus antibody (HIV-Ab), and treponema pallidum antibody. Among them, subjects with positive HBsAg and HCV-Ab should be tested for HBV-DNA and HCV-RNA virus quantitative detection;
- Total diameter of baseline target lesion.

A list of data on the demographic and baseline characteristics of the subjects is presented.

6.5 Previous / concomitant medications and previous / concomitant therapy

Previous medication/treatment refers to any other non-investigational drugs (including antineoplastic and non-antineoplastic agents)/treatment measures that ended prior to the start of the first investigational drug treatment. Concomitant medication/treatment refers to any non-investigational drug (including antineoplastic and non-antineoplastic agents)/treatment measure used at least once after the start of treatment with the investigational drug until the end of the safety follow-up period.

Previous / concomitant medication is coded using the Chinese version of WHODrug (March 1, 2020), and coded previous / concomitant medication is analyzed based on SS. The number and percentage of subjects with at least one previous / concomitant medication are summarized by cohort, and the number and percentage of subjects with at least one previous /

concomitant medication are summarized according to therapeutic class (ATC2) and preferred drug name by cohort. A list of subjects with previous / concomitant medication is presented, including subject number, cohort, drug name, ATC2, preferred drug name, previous / concomitant medication, reason for medication, dose, unit, dosage form, frequency of administration, route of administration, start date, continuity, and end date.

Previous / combined non-drug treatment is coded using MedDRA (version 23.0 or above). The number and percentage of subjects with at least one previous / combined non-drug treatment are summarized based on SS, and the number and percentage of subjects with at least one previous / combined non-drug treatment are summarized according to SOC and PT by cohort. A list of subjects with previous / combined non-drug treatment is presented, including subject number, cohort, non-drug treatment name, SOC, PT, reason for treatment, start date, continuity, and end date.

A list of subjects for follow-up anti-tumor therapy is presented.

6.6 Protocol deviation

Protocol deviation refers to the deviative actions that may affect the safety, tolerability, efficacy and pharmacokinetic results of the study treatment. It mainly includes the use of non-investigational drugs by subjects, subjects who meet the protocol exclusion criteria but are not excluded, failure to take drugs on time or poor medication compliance, blood collection time point beyond the time window, safety and efficacy parameter test beyond the time window, etc. Based on SS, protocol deviations are summarized by cohort and all subjects according to minor and major protocol deviations and types of deviations that occur, and a list of subjects with protocol deviations is presented.

6.7 Efficacy Evaluation Analysis

Based on the FAS/PPS, statistical analyses of efficacy parameters are performed by cohort, group and all subjects. The efficacy analysis consists of the following:

(1) ORR: Descriptive statistics is used to calculate the number and percentage of subjects whose response is CR or PR (i.e., CR+PR) at the optimal time point in the study as assessed by IRC and the investigator according to RECIST v1.1, respectively. The Clopper-Pearson method is used to calculate 95% CI for their percentages.

(2) DCR: Descriptive statistics is used to calculate the number and percentage of subjects whose response is CR, PR or SD (i.e., CR+PR+SD) at the optimal time point in the study as assessed by IRC and the investigator according to RECIST v1.1, respectively. The Clopper-Pearson method is used to calculate 95% CI for their percentages.

(3) PFS: Time from the first dose of investigational drug to the date of first recorded progression of disease (PD) or death from any cause. Subjects with no disease progression or death at the end of the study (or data cutoff), or subjects lost to follow-up with no confirmed tumor progression will be considered censored. The Kaplan-Meier method is used to estimate the median progression-free survival (PFS) time and its 95% confidence interval, while the number and percentage of censoring are calculated and their Kaplan-Meier survival curves are plotted.

PFS (month) = (date of PFS event or censoring - date of first dose + 1)/30.4375.

PFS censoring rules

**PFS status**	**PFS event and censoring description**	**PFS event and censoring date**
Censoring	No imaging examination after baseline and no death	Date of first dose
Censoring	Withdrawal from the study prior to initiation of new anti-cancer treatment with no evidence of disease progression or death	Date of last imaging examination of measurable lesions
Censoring	No disease progression or death prior to initiation of new anti-cancer treatment or at the time of analytical data cutoff	Date of last imaging examination of measurable lesions
Censoring	Disease progression or death after new anti-cancer treatment	Date of last imaging examination prior to initiation of new anti-cancer treatment
Censoring	If death or disease progression occurs after 2 or more consecutive tumor assessments are missing	Date of last imaging examination of measurable lesions before missing
Event	Progression is confirmed during scheduled or unscheduled follow-ups	Earliest of the following dates:<br>● Date of detection of a new lesion on imaging (if the criterion for progression is a new lesion);<br>Or<br>● Date of imaging examination corresponding to the first assessment of target/non-target lesion as progression (if imaging dates of different lesions are not on the same day, the earliest date is selected).
Event	No record of disease progression prior to death	Date of death

(4) OS: defined as the time from the first dose of investigational drug to the date of death

from any cause. Subjects who do not die at the end of the study (or data cutoff), or subjects lost to follow-up with no confirmed death are considered censored. The Kaplan-Meier method is used to estimate the median overall survival and its 95% confidence interval, while the number and percentage of censoring are calculated and their Kaplan-Meier survival curves are plotted.

OS (month) = (date of death / date of last confirmed survival - date of first dose + 1) / 30.4375

OS censoring rules

OS status:	OS event and censoring description	OS event and censoring date
Event	Death is recorded	Date of death
Censoring	No deaths at the end of the study or at the analysis data cutoff	Date of last confirmed survival
Censoring	Subjects who are lost to follow-up are not confirmed dead	Date of last confirmed survival

(5) DOR: Time from the first assessment of tumor as objective response (CR or PR) to the first assessment as PD or death from any cause before PD. Subjects with no disease progression or death at the end of the study (or data cutoff), or subjects lost to follow-up with no confirmed tumor progression will be considered censored. The Kaplan-Meier method is used to estimate the median duration of response and its 95% confidence interval, while the number and percentage of censoring are calculated and their Kaplan-Meier survival curves are plotted. DOR is evaluated only in subjects who achieved objective response (CR or PR).

DOR (month) = (date of DOR event or censoring - date of first assessment of objective response + 1)/30.4375.

DOR censoring rules

DOR status:	DOR event and censoring description	DOR event and censoring date
Censoring	Withdrawal from the study prior to initiation of new anti-cancer treatment with no evidence of disease progression or death	Date of last imaging examination of measurable lesions
Censoring	No disease progression or death prior to initiation of new anti-cancer treatment or at the time of analytical data cutoff	Date of last imaging examination of measurable lesions
Censoring	Disease progression or death after new anti-cancer treatment	Date of last imaging examination prior to initiation of new anti-cancer treatment
Censoring	If death or disease progression occurs after 2 or more consecutive tumor assessments	Date of last imaging examination of

	are missing	measurable lesions before missing
Event	Progression is confirmed during scheduled or unscheduled follow-ups	Earliest of the following dates:<br>● Date of detection of a new lesion on imaging (if the criterion for progression is a new lesion);<br>Or<br>● Date of imaging examination corresponding to the first assessment of target/non-target lesion as progression (if imaging dates of different lesions are not on the same day, the earliest date is selected).
Event	No record of disease progression prior to death	Date of death

(6) Change in target lesion from baseline: The best percentage of changes from baseline in total target lesion diameter is calculated for each subject, and a waterfall plot of the best response to tumor treatment is drawn in which different cohorts are distinguished by different colors. Best percentage of change in total target lesion diameter from baseline = (minimum total target lesion diameter after treatment - total target lesion diameter at baseline)/total baseline target lesion diameter × 100%.

(7) Subgroup analysis: Subgroup analysis of ORR, DCR, DOR, PFS and OS will be performed for the following selected characteristics by cohort and group to explore the potential impact of these factors.

- Number of lines of therapy (patients on first-line therapy, patients on second-line therapy and above, and patients who have received at least one prior systemic chemotherapy)
- Brain metastasis (Yes, No)

6.8 Safety Analysis

Based on SS, statistical analyses of safety parameters are performed by cohort, group and all subjects. The safety analysis consists of the following:

6.8.1 Drug exposure

Duration of drug exposure (day), cumulative dose (mg), average daily dose (mg/day), and mean, standard deviation, median, maximum and minimum of relative dose intensity of JMT101 are calculated. The relative dose intensity is categorized as <80%, ≥80% - ≤ 120%, and >120%, and the number and percentage of subjects in each category are summarized, where:

Duration of exposure (day) = date of last dose - date of first dose + 1.

Cumulative dose (mg) is the sum of the recorded actual doses administered at each visit;

Average daily intensity (mg/day) = cumulative dose (mg) / duration of exposure (day);

Total planned dose (mg) is the sum of the recorded planned doses administered at each visit;

Relative dose intensity = cumulative dose / total planned dose * 100%.

The changes in dosing regimen such as dose modification and infusion interruption are summarized, and the number and percentage of subjects are calculated.

Duration of drug exposure (day), cumulative dose (mg), and average daily dose (mg/day) are calculated for Afatinib or Osimertinib. Where:

Duration of exposure (day) = date of last dose - date of first dose + 1.

Cumulative dose (mg) = the sum of the actual doses received per day during the treatment period;

Average daily intensity (mg/day) = cumulative dose (mg) / duration of exposure (day).

6.8.2 DLT

The number of DLT cases and subjects and incidence are summarized by cohort and group based on the DLT analysis set, and a list of subjects who have DLT is presented.

6.8.3 Adverse event

All adverse events are coded with SOC and PT according to the Chinese version of the Medical Dictionary for Regulatory Activities (MedDRA) (23.0 or above). The summary of adverse events is based on treatment-emergent adverse event (TEAE). TEAE is defined as any event that is new or worsened from the start of investigational drug administration to the end of the safety follow-up period. When the occurrence date or end date of an AE is missing or partially missing, the adverse event will be considered as a TEAE unless the occurrence date or part of the end date can be determined to have occurred before the first dose. Treatment Related Adverse Event (TRAE) associated with the investigational drug (JMT101 + Afatinib/Osimertinib) is defined as an adverse event that is “definitely related, probably related, possibly related” or undetermined in relation to the investigational drug (JMT101 or Afatinib/Osimertinib).

The coded adverse events are summarized based on SS:

- The number of cases, subjects and incidence of all AEs, all TEAEs/TRAEs, serious TEAEs/TRAEs, TEAEs/TRAEs with a CTCAE grade ≥3, TEAEs related to protocol process and clinical operations, TEAEs leading to JMT101 dose interruption/permanent discontinuation/dose reduction, TEAEs leading to dose interruption/permanent discontinuation/dose reduction for Afatinib/Osimertinib, TEAEs/TRAEs leading to subject discontinuation, TEAEs/TRAEs leading to death, and infusion-related reactions are described by cohort and group.
- The number of cases, subjects and incidence of JMT101-related TEAEs, serious JMT101-related TEAEs, JMT101-related TEAEs with a CTCAE grade ≥3, JMT101-related TEAEs leading to JMT101 dose interruption/permanent discontinuation/dose reduction, JMT101-related TEAEs leading to subject discontinuation, and JMT101-related TEAEs leading to death are described by cohort and group.
- The number of cases, subjects and incidence of TEAEs associated with Afatinib/Osimertinib, serious TEAEs associated with Afatinib/Osimertinib, TEAEs associated with Afatinib/Osimertinib with a CTCAE grade ≥3, TEAEs associated with Afatinib/Osimertinib leading to Afatinib/Osimertinib dose interruption/permanent discontinuation/dose reduction, TEAEs associated with Afatinib/Osimertinib leading to subject discontinuation, and TEAEs associated with Afatinib/Osimertinib leading to death are described by cohort and group.
- The number of cases, subjects and incidence of all TEAEs/TRAEs, serious TEAEs/TRAEs, TEAEs/TRAEs with a CTCAE grade ≥3, TEAEs related to protocol process and clinical operations, TEAEs leading to JMT101 dose interruption/permanent discontinuation/dose reduction, TEAEs leading to dose interruption/permanent discontinuation/dose reduction for Afatinib/Osimertinib, TEAEs/TRAEs leading to subject discontinuation, TEAEs/TRAEs leading to death, and infusion-related reactions are summarized according to SOC and PT by cohort and group.
- The number of cases, subjects and incidence of JMT101-related TEAEs, serious JMT101-related TEAEs, JMT101-related TEAEs with a CTCAE grade ≥3, JMT101-related TEAEs leading to JMT101 dose interruption/permanent discontinuation/dose reduction,

JMT101-related TEAEs leading to subject discontinuation, and JMT101-related TEAEs leading to death are summarized according to SOC and PT by cohort and group.

- The number of cases, subjects and incidence of TEAEs associated with Afatinib/Osimertinib, serious TEAEs associated with Afatinib/Osimertinib, TEAEs associated with Afatinib/Osimertinib with a CTCAE grade ≥3, TEAEs associated with Afatinib/Osimertinib leading to Afatinib/Osimertinib dose interruption/permanent discontinuation/dose reduction, TEAEs associated with Afatinib/Osimertinib leading to subject discontinuation, and TEAEs associated with Afatinib/Osimertinib leading to death are summarized according to SOC and PT by cohort and group.
- The number of cases and incidence of TEAEs/TRAEs, JMT101-related TEAEs, and Afatinib/Osimertinib-related TEAEs are summarized according to SOC, PT, and CTCAE severity by cohort and group. Adverse events with the same term (SOC or PT) in a subject is counted only once at the highest severity.

A list of TEAEs, TRAEs, SAEs, and TEAEs leading to death is presented, including subject number, name of adverse event, SOC, PT, start date, end date, whether it is a TEAE, severity, whether it is a serious adverse event (SAE), correlation with JMT101, correlation with Afatinib/Osimertinib, correlation with protocol process and clinical operations, measures taken for JMT101, measures taken for Afatinib/Osimertinib, other specific treatment for adverse events, AE outcome, end date, and whether it leads to discontinuation.

6.8.4 Laboratory tests

Laboratory tests on the subjects are described. The test results at each post-baseline visit time point and at the last visit time point are classified according to the judgment of clinical significance, i.e., normal, abnormal but not clinically significant, abnormal and clinically significant, and not investigated. The number of cases and percentage are calculated separately using a shift table compared with the laboratory results of the baseline period. The number of cases and percentage of baseline results and worst post-baseline results (normal, abnormal but not clinically significant, abnormal and clinically significant, and not investigated) for laboratory test parameters are shown in a shift table. The worst post-baseline result refers to the worst result that occurs during the treatment period, including scheduled

visits and unscheduled visits. In the table, abnormal/- means abnormal but not clinically significant, and abnormal/+ means abnormal and clinically significant. The results of each parameter of blood routine, blood biochemistry and coagulation tests at each visit are described, including mean, standard deviation, median, quartiles, maximum value, minimum value and changes from baseline. A list of all laboratory tests is presented.

Time points for laboratory tests include: screening phase, C1D15, C2D1, C2D15, C3D1, C3D15, C4D1, C4D15, every 14 days of subsequent cycles, and end-of-treatment visit. Last visit is defined as the last visit of all visits, including scheduled visits, unscheduled visits, and end-of-treatment visit.

Laboratory tests include the following parameters:

Routine blood: red blood cell count, hemoglobin, hematocrit, mean corpuscular volume, mean corpuscular hemoglobin, mean corpuscular hemoglobin concentration, white blood cell, platelet, absolute neutrophil count, absolute lymphocyte count.

Blood biochemistry: alanine aminotransferase, aspartate aminotransferase, total bilirubin, direct bilirubin, alkaline phosphatase, lactate dehydrogenase, creatine kinase, gamma-glutamyl transpeptidase, total protein, albumin, total cholesterol, triglyceride, urea or urea nitrogen, creatinine, creatinine clearance, glucose, potassium, sodium, chloride, calcium, magnesium.

Routine urine: pH, specific gravity, urine glucose, urine protein, urine red blood cell, urine white blood cell, urine ketone.

Coagulation: prothrombin time, activated partial thromboplastin time, thrombin time, fibrinogen.

The following laboratory test results are graded according to NCI-CTCAE Version 5.0: routine blood (decreased/increased hemoglobin, decreased/increased white blood cells, decreased platelet count, decreased neutrophil count, decreased/increased lymphocyte count), blood biochemistry (increased alanine aminotransferase, increased aspartate aminotransferase, increased total bilirubin, increased alkaline phosphatase, increased lactate dehydrogenase, decreased albumin, increased cholesterol, increased triglyceride, increased blood urea nitrogen or urea, increased creatinine, decreased/increased glucose, decreased/increased

potassium, decreased/increased sodium, decreased/increased calcium, decreased/increased magnesium), coagulation (prolonged activated partial thromboplastin time, decreased fibrinogen). The number of cases and percentage of baseline results of the laboratory test parameter CTCAE grade and worst post-baseline results of CTCAE grade are shown in a shift table. The post-baseline worst result visits include scheduled visits and unscheduled visits.

6.8.5 12-lead ECG

The heart rate, PR interval, QRS interval, QT interval, and QTcF interval of the 12-lead ECG at each post-baseline visit time point and at the last visit time point are described, including mean, standard deviation, median, quartiles, maximum value, minimum value and changes from baseline.

The results of the 12-lead ECG at each post-baseline visit time point and at the last visit time point are described and classified as normal, abnormal but not clinically significant, and abnormal and clinically significant. The number of cases and percentage are calculated separately using a shift table compared with the results of 12-lead ECG at the baseline period. The number of cases and percentage of baseline results and worst post-baseline results (including scheduled visits and unscheduled visits) are shown in a shift table.

The maximum values of QTcF interval for each visit (including scheduled visits and unscheduled visits) after subject administration are calculated. The number and percentage of subjects with maximum values >450 ms, >480 ms, and >500 ms, and the number and percentage of subjects with change in maximum values ≤30 ms, >30 and ≤60 ms, >60 ms from baseline are summarized.

Time points for 12-lead ECG examination include: screening phase, C1D15, C2D1, C2D15, C3D1, C3D15, C4D1, C4D15, every 14 days of subsequent cycles, and end-of-treatment visit. Last visit is defined as the last visit of all visits, including scheduled visits, unscheduled visits, and end-of-treatment visit.

A list of data from 12-lead ECG results for each visit is presented.

6.8.6 Physical examination

The clinical assessment results of each physical examination parameter (general condition, skin mucosa, lymph node, head and neck, chest, abdomen, spine, muscles and

skeletons, nervous system, and other parts) at each post-baseline visit time point and at the last visit time point are described and classified as normal, abnormal but not clinically significant, abnormal and clinically significant, and not investigated. The number of cases and percentage are calculated separately using a shift table compared with the results of baseline period. The number of cases and percentage of baseline results and worst post-baseline results for each parameter (including scheduled visits and unscheduled visits) are shown in a shift table.

Time points for physical examination include: screening phase, C1D15, C2D1, C2D15, C3D1, C3D15, C4D1, C4D15, every 14 days of subsequent cycles, and end-of-treatment visit. Last visit is defined as the last visit of all visits, including scheduled visits, unscheduled visits, and end-of-treatment visit.

A list of data from physical examination results for each visit is presented.

6.8.7 Vital sign

The vital signs (body temperature, pulse, systolic blood pressure, diastolic blood pressure) for each visit are described, including mean, standard deviation, median, quartiles, maximum value, minimum value and changes from baseline. Time points for vital sign examination include: screening phase, before C1D1 administration (-30 min) and 2h (±30 min), 4h (±30 min) and 8h (±30 min) after the end of administration; C1D15, C2D1, C2D15, C3D1, C3D15, C4D1, C4D15; every 14 (±2) days of subsequent cycles and end-of-treatment visit. A list of data from vital signs for each visit is presented.

The mean, standard deviation, median, quartiles, maximum value, minimum value and changes from baseline of weight are described. Time points for weight measurement include: screening phase, the end of each treatment cycle, and end-of-treatment visit. A list of data from weight for each visit is presented.

6.8.8 ECOG score

The results of ECOG performance status at each post-baseline visit time point and at the last visit time point are scored according to the criteria and compared with baseline ECOG performance status score with a crossover table calcutlate the number of cases and percentage of baseline results and worst post-baseline results for each parameter (including scheduled visits and unscheduled visits) are shown in a shift table. Time points for ECOG performance

status examination include: screening phase, C1D1, C1D15, C2D1, C2D15, C3D1, C3D15, C4D1, C4D15, every 14 days of subsequent cycles, and end-of-treatment visit. Last visit is defined as the last visit of all visits, including scheduled visits, unscheduled visits, and end-of-treatment visit.

A list of ECOG score for each visit is presented.

6.8.9 Echocardiography

The results of the echocardiography at each post-baseline visit time point and at the last visit time point are described and classified as normal, abnormal but not clinically significant, and abnormal and clinically significant. The number of cases and percentage are calculated separately using a shift table compared with the results. The number of cases and percentage of baseline results and worst post-baseline results (including scheduled visits and unscheduled visits) are shown in a shift table. Time points for echocardiography include: screening phase, the end of every 3 treatment cycles, and end-of-treatment visit. Last visit is defined as the last visit of all visits, including scheduled visits, unscheduled visits, and end-of-treatment visit.

A list of echocardiography for each visit is presented.

6.8.10 Pregnancy test

A list of data from pregnancy test results of women of childbearing age for each visit is presented.

6.9 Immunogenicity Study

The number and incidence of positive detections of anti-drug antibodies (ADA) and neutralizing antibodies (NAbs) (if applicable) are described by each time point based on the immunogenicity analysis set. A list of immunogenicity data of subjects is presented.

6.10 Biomarker parameter analysis

The cut-off value is determined based on the level of free DNA in plasma and the low and high expression groups are identified based on this value. The Kaplan-Meier method is used to estimate the median PFS, median OS and their 95% confidence intervals for all subjects and each dose group based on the biomarker analysis set, and survival curves are plotted.

6.11 Handling of missing values

（1） AE date missing

Start date missing:

· If the "year and month" are known and the "year and month" are equal to the "year and month" when the investigational drug was first administrated, make the start date of AE equal to the date when the investigational drug was first administrated.

· If the "year and month" are known, but the "year and month" are different from the "year and month" when the investigational drug was first administrated, it should be imputed with the first day of that month

· If only the "year" is known and the "year" is equal to the "year" when the investigational drug was first administrated, make the start date of AE equal to the date when the investigational drug was first administrated.

· If only the "year" is known, but the "year" is different from the "year" when the investigational drug was first administrated, it should be imputed with January 1 of that year.

· If the "year, month, and day" are missing, the date when the investigational drug was first administrated is used as the corresponding start date.

· If the AE end date is not missing and the imputed AE start date is after the AE end date, the AE start date is imputed as the AE end date.

End date missing

· If the "year, month, and day" are all missing (e.g., AE is still ongoing), it should be imputed with the earliest of the following dates: data cutoff date, discontinuation date + 30 days, and date of death.

· If the "year" is not missing but the month and day are missing, it should be imputed with the earliest of the following dates: December 31, the data cutoff date, and the date of death.

· If only the "day" is missing, it should be imputed with the earliest of the following dates: the last day of that month, the data cutoff date, and the date of death.

(2) Date of previous / concomitant medications and date of previous / concomitant therapy missing

Start date missing:

- When the “year, month, and day” are all missing, the imputation is not performed.
- When only the “year” exists or only the “year and day” exists, it should be imputed with January 1st;
- When only the “year and month” exists, it should be imputed with the first day of the starting month.

End date missing:

- If the “year and month” are known, it should be imputed with the last day of the month.
- If only the “year” are known, it should be imputed with December 31st.
- If the “year, month and day” are all missing, it should be considered missing.
- If the imputed end date is before the start date, the start date is used as the corresponding end date.

(3) Date of death missing

- If the “year, month and day” are all missing, then (last confirmed date of survival + 1 day) is used to impute.
- If the “day” is missing or the “month and day” are missing, it should be imputed with the earliest of the following dates:

➢ Last confirmed date of survival +1

➢ If only the “day” is missing, it should be imputed with the first day of the non-missing year and month; if both the “month and day” are missing or only the “month” is missing, it should be imputed with January 1st of the non-missing year.

The above imputation rules are only used when exact dates are needed for calculation and analysis, the list is still presented as the actual data entered in the database.

6.12 Processing of outlier

Outliers are not identified and processed in this study (except where specifically stated).

7. INTERIM ANALYSIS

The study is divided into two stages: Stage I dose escalation study and Stage II dose expansion study. Statistical analyses are performed separately by study stage.

8. ABOUT THIS PLAN

This analysis plan is based on the statistic relevant descriptions of the protocol, the

statistical analysis methods are in accordance with the basic characteristics of each parameter in the protocol and the specific requirements of the study. Due to unpredictable changes in the final form of data distribution in clinical studies, the actual statistical analysis may also be changed to some extent.
