# Supplementary material for: Phase 1b trial of anti-EGFR antibody JMT101 and Osimertinib in EGFR exon 20 insertion-positive non-small-cell lung cancer
Source: Nat Commun. 2023 Jun 12;14:3468. doi: 10.1038/s41467-023-39139-4 (PMC10261012; doi:10.1038/s41467-023-39139-4)
Supplement: Supplementary file 2 — Reporting Summary [file 41467_2023_39139_MOESM2_ESM.pdf]

nature portfolio

Corresponding author(s): Fang W, Huang Y, Zhang L, Yang X

Last updated by author(s): May 24, 2023

# Reporting Summary

Nature Portfolio wishes to improve the reproducibility of the work that we publish. This form provides structure for consistency and transparency in reporting. For further information on Nature Portfolio policies, see our Editorial Policies and the Editorial Policy Checklist.

## Statistics

For all statistical analyses, confirm that the following items are present in the figure legend, table legend, main text, or Methods section.

| n/a | Confirmed | |
|---|---|---|
| ☐ | ☒ | The exact sample size (*n*) for each experimental group/condition, given as a discrete number and unit of measurement |
| ☐ | ☒ | A statement on whether measurements were taken from distinct samples or whether the same sample was measured repeatedly |
| ☐ | ☒ | The statistical test(s) used AND whether they are one- or two-sided<br>*Only common tests should be described solely by name; describe more complex techniques in the Methods section.* |
| ☒ | ☐ | A description of all covariates tested |
| ☐ | ☒ | A description of any assumptions or corrections, such as tests of normality and adjustment for multiple comparisons |
| ☐ | ☒ | A full description of the statistical parameters including central tendency (e.g. means) or other basic estimates (e.g. regression coefficient) AND variation (e.g. standard deviation) or associated estimates of uncertainty (e.g. confidence intervals) |
| ☐ | ☒ | For null hypothesis testing, the test statistic (e.g. *F*, *t*, *r*) with confidence intervals, effect sizes, degrees of freedom and *P* value noted<br>*Give P values as exact values whenever suitable.* |
| ☒ | ☐ | For Bayesian analysis, information on the choice of priors and Markov chain Monte Carlo settings |
| ☒ | ☐ | For hierarchical and complex designs, identification of the appropriate level for tests and full reporting of outcomes |
| ☒ | ☐ | Estimates of effect sizes (e.g. Cohen's *d*, Pearson's *r*), indicating how they were calculated |

*Our web collection on statistics for biologists contains articles on many of the points above.*

## Software and code

Policy information about availability of computer code

| | |
|---|---|
| Data collection | Clinical data were collected using the Medidata EDC system (version Rave EDC 2022.2.1). |

| | |
|---|---|
| Data analysis | Statistical analyses were performed using SPSS software (version 24.0.0 for Windows, IBM), SAS software (version 9.4) and R version 3.6.1 (http://cran.r-project.org).<br>IC50 values in cell viability assays were calculated using GraphPad Prism 9. Flow cytometry data were processed using FlowJo v10, BD FACSDiva Software v8.0.1 and CytExpert v2.4.<br>For DNA sequencing and data processing, targeted capture was performed using a custom set of biotinylated DNA probes, which cover 107 cancer-related genes (HapOncoCDx™, Roche). Amplified sample libraries and the SeqCap EZ Library were hybridized according to the manufacturer's protocol. Subsequently, the reactions were pooled and purified using Agencourt AMPure XP beads, and then amplified by PCR. After quantification by quantitative PCR, the library was diluted, denatured with 0.2 N NaOH, and sequenced using PE150 paired-end sequencing on the NovaSeq 6000 system (Illumina, Inc., San Diego, CA, USA). DNA fragments, library purity and concentration were assessed using a Qubit 3.0 Fluorometer and dsDNA HS Assay kit (Invitrogen, Waltham, MA, United States). Fragment length was determined on a 4200 Bioanalyzer (Agilent Technologies, Santa Clara, CA, USA). cfDNA-seq was performed using a targeted next-generation sequencing panel on 107 lung cancer-related genes (HapOnco™ 107 panel) with a mean coverage of 2,000×, and the mean coverage for gDNA is 1,000×.<br>Raw cfDNA sequencing data were pre-processed by fastp v0.12.6 version 0.18.0 (https://github.com/OpenGene/fastp). Clean reads were aligned to the hg19 genome (GRch37) using Burrows-Wheeler Aligner version 0.7.15-r1140. The Gencore version 0.12.0 (https://github.com/OpenGene/gencore) were used to remove duplicate reads. Samtools version 0.1.19 (http://www.htslib.org/) was applied to generate pileup files for properly paired reads with mapping quality ≥60. Somatic variants calling was performed using VarScan2 version 2.3.8 (http://varscan.sourceforge.net/). Artifacts were removed by a manual inspection using GenomeBrowse ® visualization tool (Version 2.x). Somatic mutation calls were annotated using ANNOVAR version 2018-04-16. CNVkit version 0.9.3 (https://github.com/etal/cnvkit) was used for copy number variation detection. GeneFuse version v0.6.1 was used for structural variation detection (https://github.com/OpenGene/GeneFuse). |

For manuscripts utilizing custom algorithms or software that are central to the research but not yet described in published literature, software must be made available to editors and reviewers. We strongly encourage code deposition in a community repository (e.g. GitHub). See the Nature Portfolio guidelines for submitting code & software for further information.

# Data

Policy information about availability of data

All manuscripts must include a data availability statement. This statement should provide the following information, where applicable:

- Accession codes, unique identifiers, or web links for publicly available datasets
- A description of any restrictions on data availability
- For clinical datasets or third party data, please ensure that the statement adheres to our policy

The publicly available databases utilized for the biomarker analyses in this study include OncoKB (https://www.oncokb.org/)54, COSMIC (https://cancer.sanger.ac.uk/cosmic)55 and KEGG pathways (https://www.kegg.jp/kegg/pathway.html)56. The raw DNA-sequencing data generated in the study have been deposited in the China National Center for Bioinformation (http://bigd.big.ac.cn/) under the project number: PRJCA010856 (https://ngdc.cncb.ac.cn/gsa-human/browse/HRA002822). Sequencing or de-identified patient-level data are available under restricted access. Access can be obtained by completing the application form via GSA-Human System (for sequencing data) and/or by contacting fangwf@sysucc.org.cn or zhangli@sysucc.org.cn. All requests will be reviewed by corresponding authors, the SYSUCC institutional review board and CSPC Pharmaceutical Group Co., Ltd.. A signed data access agreement with the sponsors is required before data sharing. The complete protocol and statistical analysis plan are available in the Supplementary Note. The remaining data are available within the Article, Supplementary Information or Source Data file. Source data are deposited into Figshare (https://doi.org/10.6084/m9.figshare.22691635) and are provided with this paper.

# Human research participants

Policy information about studies involving human research participants and Sex and Gender in Research.

| | |
|---|---|
| Reporting on sex and gender | This is not a sex-specific study. Study results were applicable for both sexes. |
| Population characteristics | Eligible patients were aged ≥18 years, had histologically or cytologically confirmed stage IIIB or IV non-small-cell lung cancer (NSCLC) harboring EGFR exon 20 insertions (20ins), had at least one measurable lesion defined by Response Evaluation Criteria in Solid Tumors v.1.1 (RECIST v.1.1) and Eastern Cooperative Oncology Group (EGOG) performance status of 0-1. Patients with asymptomatic brain or leptomeningeal metastasis were allowed. Key exclusion criteria included prior treatment with anti-EGFR monoclonal antibodies, concurrent EGFR mutations that were reported to be responsive to approved EGFR tyrosine kinase inhibitor (TKI) (eg, exon 19 deletion, L858R, T790M, L861Q, G719X, S768I), use of immune checkpoint inhibitors within 3 months (for JMT101+Osimeritnib cohorts) and patients who had derived clinical benefits from previous EGFR-TKI treatments (CR, PR, or SD≥6 months). Complete eligibility criteria are available in the study protocol (supplementary Information). |
| Recruitment | A total of 169 patients were screened for eligibility at 15 participating sites in China. Patients were recruited via competitive enrollment. All patients who met the eligibility requirements stated in the protocol were informed of the study. Finally, a total of 150 patients were eligible and enrolled into the study.<br>Although all centers enrolled patients according to the protocol, there may still be subtle differences in the implementation of individual enrollment criteria by investigators from different centers, resulting in some degree of heterogeneity in patients from different centers. In general, these differences had little impact on the final results of the study. |
| Ethics oversight | This study was conducted in accordance with Good Clinical Practice guidelines and the Declaration of Helsinki. The study protocol and all amendments were approved by institutional review boards at all participating sites, including Sun Yat-sen University Cancer Center, Fujian Cancer Hospital, Shanghai Chest Hospital, Zhejiang Cancer Hospital, Shanxi Provincial Cancer Hospital, West China Hospital, Hunan Cancer Hospital, Chinese PLA General Hospital, Henan Cancer Hospital, Union Hospital of Tongji Medical College, Hebei Tumor Hospital, Nanjing Drum Tower Hospital, Jiangsu Province Hospital of Chinese Medicine, Shanxi Bethune Hospital and Renmin Hospital of Wuhan University. All patients provided written informed consent |

before enrollment.

Note that full information on the approval of the study protocol must also be provided in the manuscript.

# Field-specific reporting

Please select the one below that is the best fit for your research. If you are not sure, read the appropriate sections before making your selection.

[x] Life sciences [ ] Behavioural & social sciences [ ] Ecological, evolutionary & environmental sciences

For a reference copy of the document with all sections, see nature.com/documents/nr-reporting-summary-flat.pdf

# Life sciences study design

All studies must disclose on these points even when the disclosure is negative.

| | |
|---|---|
| Sample size | Stage I: This is the the dose-escalation stage. It followed the conventional 3+3 dose-escalation design and enrolled a total of 12 patients (3 in each cohort). The first 12 patients were enrolled alternatively into the four cohorts. There is no predetermination of sample size.<br>Stage II: This is the dose-expansion stage. For cohorts that were considered tolerable in stage I (no DLT was observed), additional enrollment (≤ 12 patients in total) were allowed in the dose-expansion stage. Safety and efficacy data were monitored periodically. One of the cohorts would be eventually selected for further expansion based on safety, tolerability and efficacy signals.<br>The total sample size required for the efficacy population was determined using the normal approximation method. Given the current treatment landscape of EGFR 20ins, the combination therapy is required to have an ORR≥35% to warrant its further development. Assuming that the expected ORR is 40%, when the sample size reach 120 in the efficacy population, the probability of observed ORR>35% by normal approximation method is above 85% (86.8%). |
| Data exclusions | No data was excluded from the analysis. |
| Replication | For the in vitro study, each experiment was replicated three separate times. For the in vivo study, each treatment group contained eight mice. All attempts at replication were successful. |
| Randomization | For the in vivo study, mice were continuously randomized into treatment groups when their tumors reached the indicated sizes.<br>For the in vitro study, randomization was not relevant because samples were processed in parallel or sequentially.<br>For the clinical study, randomization was not relevant because this was an open-label, phase 1b, dose-escalation and dose-expansion trial. |
| Blinding | Blinding or randomization was not relevant because this was an open-label, phase 1b, dose-escalation and dose-expansion trial.<br>In the dose-escalation stage (stage I), patients were enrolled sequentially into each cohort. For cohorts that were considered tolerable in stage I, additional enrollment were allowed in the dose-expansion stage. Safety and efficacy data were monitored periodically. One of the cohorts would be eventually selected for further expansion based on safety, tolerability and efficacy signals. |

# Reporting for specific materials, systems and methods

We require information from authors about some types of materials, experimental systems and methods used in many studies. Here, indicate whether each material, system or method listed is relevant to your study. If you are not sure if a list item applies to your research, read the appropriate section before selecting a response.

## Materials & experimental systems

| n/a | Involved in the study | |
|---|---|---|
| [ ] | [x] | Antibodies |
| [ ] | [x] | Eukaryotic cell lines |
| [x] | [ ] | Palaeontology and archaeology |
| [ ] | [x] | Animals and other organisms |
| [ ] | [x] | Clinical data |
| [x] | [ ] | Dual use research of concern |

## Methods

| n/a | Involved in the study | |
|---|---|---|
| [x] | [ ] | ChIP-seq |
| [ ] | [x] | Flow cytometry |
| [x] | [ ] | MRI-based neuroimaging |

## Antibodies

| | |
|---|---|
| Antibodies used | Rabbit anti-pEGFR (1:1000; Cell Signaling Technology, #3777S, Rabbit monoclonal [D7A5], lot:16)<br>Rabbit anti-EGFR (1:1000; Cell Signaling Technology, #4267S, Rabbit monoclonal [D38B1], lot:24)<br>Rabbit anti-pAKT (1:1000; Cell Signaling Technology, #9271S, lot:15)<br>Rabbit anti-AKT(1:1000; Cell Signaling Technology, #4691S, Rabbit monoclonal [C67E7], lot:28)<br>Rabbit anti-pERK (1:2000; Cell Signaling Technology, #4370S, Rabbit monoclonal [D13.14.4E], lot:24)<br>Rabbit anti-ERK (1:1000; Cell Signaling Technology, #4695S, Rabbit monoclonal [137F5], lot:28)]<br>Rabbit anti-GAPDH (1:10000; Proteintech, #10494-1-AP) |

| | |
|---|---|
| | Alexa Fluor 488 anti-human EGFR (5ul/test; BioLegend, #352908, lot: B332782)<br>Alexa Fluor 488 Mouse IgG1, κ Isotype Ctrl (FC) Antibody (5ul/test; BioLegend, #400129, lot: B354284)<br>Human IgG1, kappa Isotype Control (SinoBiological, #HG1K, R1 clone, lot: MA16MY1804, dilution details are provided in the Source data file for Fig s1f) |
| Validation | Rabbit anti-pEGFR, #3777S, Cell Signaling, 1:1000, Validated: https://www.cellsignal.cn/products/primary-antibodies/phospho-egf-receptor-tyr1068-d7a5-xp-rabbit-mab/3777<br>Rabbit anti-EGFR, #4267S, Cell Signaling, 1:1000, Validated: https://www.cellsignal.cn/products/primary-antibodies/egf-receptor-d38b1-xp-rabbit-mab/4267<br>Rabbit anti-pAKT, #9271S, Cell Signaling, 1:1000, Validated: https://www.cellsignal.cn/products/primary-antibodies/phospho-akt-ser473-antibody/9271<br>Rabbit anti-AKT, #4691S, Cell Signaling, 1:1000, Validated: https://www.cellsignal.cn/products/primary-antibodies/akt-pan-c67e7-rabbit-mab/4691<br>Rabbit anti-pERK, #4370S, Cell Signaling, 1:2000, Validated: https://www.cellsignal.cn/products/primary-antibodies/phospho-p44-42-mapk-erk1-2-thr202-tyr204-d13-14-4e-xp-rabbit-mab/4370<br>Rabbit anti-ERK, #4695S, Cell Signaling, 1:1000, Validated: https://www.cellsignal.cn/browse/?N=4294956287&Ntk=Products&Ntt=4695&site-search-type=Products<br>Rabbit anti-GAPDH, #10494-1-AP, Proteintech, 1:10000, Validated: https://www.ptglab.com/products/GAPDH-Antibody-10494-1-AP.htm<br>Alexa Fluor 488 anti-human EGFR, #352908, BioLegend, Validated: https://www.biolegend.com/en-us/products/alexa-fluor-488-anti-human-egfr-antibody-7715<br>Alexa Fluor 488 Mouse IgG1, κ Isotype Ctrl (FC) Antibody, #400129, BioLegend, Validated: https://www.biolegend.com/en-us/products/alexa-fluor-488-mouse-igg1-kappa-isotype-ctrl-fc-2687<br>Human IgG1, kappa Isotype Control Antibody, #HG1K, SinoBiological, Validated: https://cn.sinobiological.com/antibodies/human-igg1-kappa-isotype-control-hg1k |

## Eukaryotic cell lines

Policy information about cell lines and Sex and Gender in Research

| | |
|---|---|
| Cell line source(s) | All mutant Ba/F3 cell lines were obtained from the KYinno BIOTECHNOLOGY (https://www.kyinno.com/, #KC1050, #KC1025, #KC1024). Mutant Ba/F3 cell lines were generated using retrovirus vector expressing specific EGFR sequence. |
| Authentication | For EGFR mutant Ba/F3 cell lines, to confirm the presence of the EGFR mutation, RNA was extracted from cells using the TaKaRa MiniBEST Universal (TaKaRa, #9767) according to manufacturer's instructions. cDNA was synthesized using the TaKaRa PrimeScript™ RT reagent Kit (TaKaRa, #RR047A) and used as a template to amplify EGFR. PCR product was sequenced by Sanger sequencing using the following primers: CTGTGCCATCCAAACTGCAC and GAGGGAGGCGTTCTCCTTTC. |
| Mycoplasma contamination | Cell lines were all tested and were found to be free of mycoplasma. |
| Commonly misidentified lines (See ICLAC register) | No commonly misidentified cell lines were used |

## Animals and other research organisms

Policy information about studies involving animals; ARRIVE guidelines recommended for reporting animal research, and Sex and Gender in Research

| | |
|---|---|
| Laboratory animals | Female BALB/c nude mice at 6 to 8 weeks of age were obtained from Shanghai Lingchang Biotechnology Co. Ltd.. The mice were housed in SPF-class independent ventilation cage (4 animals per cage). They were reared at 20-26°C with a humidity of 40-70%, 12/12 dark/light cycles, and had free access to food and water ad libitum. Ba/F3 cells expressing EGFR A767_V769dup (5×10^5 cells) were injected subcutaneously into the BALB/c nude mice. |
| Wild animals | The study did not involve wild animals. |
| Reporting on sex | This is not a sex-specific study. We believed that study results were applicable for both sexes. |
| Field-collected samples | The study did not involve field-collected samples. |
| Ethics oversight | The animal experiment was approved by the Sun Yat-sen University Cancer Center Animal Ethics Committee and handled in accordance with Good Animal Practices. |

Note that full information on the approval of the study protocol must also be provided in the manuscript.

## Clinical data

Policy information about clinical studies

All manuscripts should comply with the ICMJEguidelines for publication of clinical research and a completedCONSORT checklist must be included with all submissions.

| | |
|---|---|
| Clinical trial registration | NCT04448379 |

| | |
|---|---|
| Study protocol | The complete protocol and statistical analysis plan were submitted as the Supplementary Information |
| Data collection | Data were collected at individual participating sites by study site staffs using the Medidata EDC system. Patients were enrolled from 15 sites in China between June 29, 2020, and December 28, 2021. The date of data cutoff for this study was May 31, 2022. Data collection ran from enrollment of the first patient (June 2020) to data cutoff (May 2022). |
| Outcomes | The primary objective was to evaluate safety and tolerability of JMT101 plus afatinib or osimertinib in advanced or metastatic NSCLC harboring EGFR 20ins. Secondary objectives included anti-tumor activity measured by tumor responses (ORR, DCR), duration of response (DOR), progression-free survival (PFS) and overall survival (OS), pharmacokinetics, immunogenicity, and biomarkers potentially associated with clinical outcomes.<br>Adverse events were monitored throughout the study until 30 days after the last dose, and were graded according to the National Cancer Institute Common Terminology Criteria for Adverse Events (NCI CTCAE), version 5.0. Disease assessment by radiologic imaging was conducted at screening, 4 weeks of study treatment and every 8 weeks thereafter. Tumor response was determined by a central independent review committee (IRC) and investigators per RECIST v1.131. After disease progression, patients were followed up for survival every 8 weeks. |

# Flow Cytometry

## Plots

Confirm that:

- [x] The axis labels state the marker and fluorochrome used (e.g. CD4-FITC).
- [x] The axis scales are clearly visible. Include numbers along axes only for bottom left plot of group (a 'group' is an analysis of identical markers).
- [x] All plots are contour plots with outliers or pseudocolor plots.
- [x] A numerical value for number of cells or percentage (with statistics) is provided.

## Methodology

| | |
|---|---|
| Sample preparation | For Extended Data Fig 1f: Primary NK cells were isolated from PBMC; Target cells were collected and stained with Celltrace-Violet. Then target cells were incubated with the indicated agents for 30min; Target cells were co-cultured with NK cells at 37℃; Cells were then collected at 4 hours for analysis.<br>For Extended Data Fig 3: Ba/F3 cells were treated with the indicated agents for 6 hours or 24hours and collected for analysis. |
| Instrument | For Extended Data Fig 1f: BD LSRFortessa™ X-20 Cell Analyzer; For Extended Data Fig 3: CytoFLEX LX |
| Software | FlowJo v10 and BD FACSDiva Software v8.0.1 (Extended Data Fig 1f); CytExpert v2.4 and FlowJo v10 (Extended Data Fig 3) |
| Cell population abundance | For Extended Data Fig 1f: NK cells were detected. Purity was evaluated using flow cytometry and was>98%.<br>For Extended Data Fig 3: Ba/F3 cells expressing EGFR were detected. Purity was evaluated using flow cytometry and was >98%. |
| Gating strategy | For Extended data Figure 1f:<br>FFC x SSC gate on total cells--><br>FSC-A x FSC-H gated on single cells--><br>Celltrace-BV421 x SSC-A gated on tumor cells--><br>PI-PE gated on dead tumor cells.<br>For Extended Data Figure 3:<br>FSCxSSC gated on live Ba/F3 cells --><br>SSC-H x SSC-A gated on single cells --><br>EGFR-FITC gated on live EGFR+ cells. |

- [x] Tick this box to confirm that a figure exemplifying the gating strategy is provided in the Supplementary Information.
